# Spatial confinement promotes germinal center diversity and tempers immunodominance

**DOI:** 10.1038/s41590-026-02698-x

**Published:** 2026-10-21

**Authors:** Kevin Y. Chen, Kenneth H. Hu, Nikhita Kirthivasan, Anshul Rao, Niklas Schwan, Jinping An, Ying Xu, Vincent Peng, Mélia Magnen, Sijin Huang, Qian Yin, Debraj Ghose, Caroline Alexander, Jack Smerczynski, Michael Meyer-Hermann, Matthew F. Krummel, Vamsee Mallajosyula, Daniel Lingwood, Jason G. Cyster

**Affiliations:** 1 Howard Hughes Medical Institute and Department of Microbiology and Immunology, University of California San Francisco, San Francisco, CA 94143, USA; 2 Department of Immunology, University of Texas MD Anderson Cancer Center, Houston, TX 77054, USA; 3 Department of Pathology, University of California, San Francisco, San Francisco, CA 94143, USA.; 4 Department of Systems Immunology, Helmholtz Centre for Infection Research (HZI), Braunschweig, Germany.; 5 Department of Gastroenterology, University of California San Francisco, San Francisco, CA 94143, USA; 6 Université de Reims Champagne-Ardenne, INSERM, P3Cell UMR-S1250, SFR CAP-SANTE, Reims 51097, France; 7 Department of Biomedical Engineering, The University of Texas at Austin, Austin, TX 78712 USA; 8 Wyss Institute for Biologically Inspired Engineering, Harvard University, Boston, MA 02215, USA; 9 The Ragon Institute of Massachusetts General Hospital, The Massachusetts Institute of Technology and Harvard University, 400 Technology Square, Cambridge, MA 02139, USA; 10 Institute for Biochemistry, Biotechnology and Bioinformatics, Technische Universität Braunschweig, Braunschweig, Germany; 11 Lower Saxony Center for Artificial Intelligence and Causal Methods in Medicine (CAIMed), Hannover, Germany.; 12 Institute for Immunity, Transplantation, and Infection, Stanford University School of Medicine, Stanford, CA 94304, USA

## Abstract

Germinal centers (GCs) form as confined clusters of motile cells within individual lymphoid follicles. The role of confinement in GC B cell evolutionary events is unknown. Using mice with reduced GC confinement due to deficiency in Gα13-dependent G-protein-coupled receptor signaling, we observed GC-to-GC trafficking in confinement-defective lymph nodes (LNs). In multi-color clonal lineage tracing mice, de-confined GCs minimally diversified their color palettes with respect to one another. Spatial B cell receptor sequencing revealed greater clonal crossover between neighboring GCs in the setting of dispersal. The total GC B cell repertoire of LNs with inter-GC exchange also decreased in overall diversity due to increased skewing towards top clones. As an outcome, LNs with de-confined GCs more strongly favored clones responding to accessible epitopes while losing clones binding to other portions of antigen. Together, our findings suggest that confinement supports antibody breadth by moderating the spread of dominant clones and preserving unique trajectories in individual GC responses.

## Introduction

Germinal centers (GCs) emerge as clusters of proliferating B cells within lymphoid follicles. In humans and macaques, responding LNs can contain over 100 GCs while in mice they range in number from several to 30 or more^1–5^. Individual GCs are seeded by 10’s to 100’s of B cells^6^. Although mature GCs appear as tightly confined clusters of a few thousand cells, the GC B cells are highly motile and active mechanisms are involved in confining them^7,8^. While the mechanisms of confinement have been elucidated to some degree, whether spatial restriction of GC B cells benefits the humoral response remains unknown. One important aspect is the impact of confinement on the evolving GC repertoire. If the system were alternately designed to allow exchange between GCs, this could promote faster refinement of the antibody response. Conversely, selection may fail if exchange disrupts successful positive selection through clonal bursting. Thus, the function and trade-offs of the observed anatomy remain unclear.

The best-defined mechanism of GC spatial confinement involves the G-protein coupled receptor (GPCR)-Gα13-signaling pathway. In mice, sphingosine-1-phosphate receptor-2 (S1PR2) is the dominant Gα13-coupled receptor expressed in GC B cells^9^. S1PR2 signals via Gα13 and Arhgef1 to activate RhoA and this antagonizes GC B cell migration to locations containing S1P^10–12^. S1P is abundant in the outer follicle and sparse in the center^8,10,13^. Imaging studies established that S1PR2-deficient GC B cells organize in GCs, but they are less completely confined than wildtype (WT) cells, with this phenotype being most studied in mesenteric LNs (mLNs)^10^. Deficiency in Gα13 or Arhgef1 led to greater mLN GC B cell deconfinement than S1PR2 deficiency, suggesting that this pathway functions downstream of additional GPCRs to promote GC confinement^14^.

Each GC is organized into a so-called light zone (LZ) and dark zone (DZ), with B cell antigen recognition and T cell help predominantly occurring in the LZ, and mutation and proliferation in the DZ. High expression of activation-induced cytidine deaminase (AID) allows DZ GC B cells to undergo somatic hypermutation (SHM) of their rearranged immunoglobulin V genes. Stringent dependence on positive signals from T follicular helper (Tfh) cells in the LZ facilitates selection of mutated cells. GC B cells presenting greater amounts of MHCII-peptide receive greater T cell help and undergo more cell cycles than other GC B cells^15^.

While this model readily explains antibody affinity maturation, there is less clarity on how the GC preserves breadth in antigen recognition, which is important for enabling the generation of antibodies that can protect against rapidly evolving pathogens^6,16–22^. Studies of antigens with accessible versus poorly accessible epitopes show that most antigen-specific B cells involved in primary GC responses are specific for accessible epitopes^16,17,23–25^. However, over time, epitope masking by secreted antibody can cause shifts in epitope accessibility and GC B cell clonal representation^26,27^. Importantly, B cells recognizing poorly accessible epitopes can often be detected during late GC responses and various mechanisms may operate to sustain these cells, though these mechanisms remain poorly understood.

Here, using recently developed tools, we perturb GC organization to investigate the consequences of relaxing physiological GC confinement. We find that GC clones can expand in other GC ‘islands’ when dispersed, and that the overall LN GC repertoire becomes less diverse in the setting of clonal spreading. However, the GC repertoire was also able to focus on an accessible epitope more effectively, thus presenting a trade-off between breadth and specificity in the tuning of GC dispersal.

## Results

### Confinement-pathway mutant GC B cells migrate between GCs

Utilizing recent advances in understanding of the Gα13 confinement pathway, we hypothesized that B cell loss of Gα13 in peripheral LN GCs would enable us to study impacts of GC de-confinement while minimizing the nutrient-driven proliferation effects specific to the mLN^28^. Following subcutaneous immunization with NP29-KLH, a strong GC-inducing immunogen, *Gna13*f/f mice harboring a B cell specific Cre (Gα13^ΔB^ mice) had popliteal LN GCs of similar size and shape to WT but GL7^+^ GC B cells could be observed dispersed in the LN (Fig. 1a and Extended Data Fig. 1a–d). GC borders became less neatly defined, with more GL7^+^ B cells spilling into the T zone and medulla in Gα13^ΔB^ LNs. GC B cells also reached across interfollicular zones towards neighboring GCs (Fig. 1a and Extended Data Fig. 1a, b).

However, static snapshots do not reveal whether de-confined GC B cells can exchange between GCs. To observe exchange directly, we used multiphoton-based LN explant imaging^29^. Here we combined GC-specific conditional (S1pr2-CreERT2 mediated) *Gna13* deletion with tdTomato reporter activation to selectively label Gα13-deficient (Gα13^ΔGCB^) or control GC B cells (Supplementary Fig. 1a–d). Mice were immunized with NP29-KLH and pulse-treated with tamoxifen at day 15 and 17 followed by imaging at day 19. At this early time point after tamoxifen treatment the fraction of labeled cells that had a memory or plasma cell (PC) phenotype was low (Supplementary Fig. 1c). During multiphoton imaging, GCs were identifiable as tight clusters of tdTomato^+^ cells (Fig. 1b). In control mice, small numbers of tdTomato^+^ cells were visible in between GCs, and these cells moved in random directions with none exchanging between GCs during the imaging period (Fig. 1b, c and Supplementary Videos 1–3). Examples could also be observed of GC B cells making arc-like paths as they left the main GC aggregate and wandered before turning back into the same GC (Fig. 1c). While limits in imaging volume and duration prevented full tracking of the same cell for extended periods of time, tracking analysis of 45 min time-lapses with 65um stacks revealed some cells that traversed the gap between adjacent GCs in Gα13^ΔGCB^ LNs but not control LNs (Fig. 1c–e and Supplementary Videos 4–6). In LNs from Gα13^ΔGCB^ mice, greater numbers of dispersed cells could be detected (Fig. 1e). The migration speeds of the extra-GC cells was 8-9 um/min (Fig. 1f), matching GC B cells and slower than Tfh cells, which typically migrate at twice the speed (15-20 um/min^29^). The Gα13^ΔGCB^ cells had a slightly increased migration speed (Fig. 1f) and increased net track displacement (Fig. 1g), both of which could contribute to the elevated likelihood of inter-GC crossing events.

To corroborate our findings in S1pr2-tdTomato mice, we utilized another labeling system involving Aicda-CreERT2 combined with the Confetti reporter palette^6^. Short-term pulse tamoxifen labelling of immunized mice in this system also led to GC-specific labelling (Extended Data Fig. 2a, Supplementary Fig. 1e–h and Supplementary Videos 7–9), although less efficiently than S1pr2-tdTomato. Instead of *Gna13* deletion, we crossed this reporter to mice deficient in Arhgef1, the downstream effector for Gα13^9^. For *Arhgef1*−/− Confetti mice, GC borders were not as well demarcated as in the Gα13^ΔGCB^ tdTomato mice, likely due to the combined effects of dispersal, lower labelling efficiency and multicolor reporting in the Confetti mice. However, GCs were still identifiable (Extended Data Fig. 2b). Amongst the Confetti colors, we used the longest wavelength emission RFP for quantification. Although track durations were still limited by cells leaving the imaging volume, trafficking events from one GC to another could be observed, but only in the *Arhgef1*−/− LNs (Extended Data Fig. 2b–d and Movies S10–S12). GC B track speeds were between 6-8 um/min, and slightly higher for *Arhgef1*−/− than +/− (Extended Data Fig. 2e). Net track displacements were also shifted higher for *Arhgef1*−/− mice (Extended Data Fig. 2f). To confirm that reporter positive cells outside GCs were GC B cell phenotype, we compared their size and morphology to naïve B cells, which are similar to quiescent memory B cells in size^30^ (Extended Data Fig. 2g). Adoptively transferred naïve lymphocytes segregated well from Confetti^+^ endogenous cells in control mice (Supplementary Videos 13–14), and in *Arhgef1*−/− mice, we measured the dispersed Confetti^+^ cells, as well as inter-GC crossing cells, to be larger in volume (~12um diameter) and less spherical compared to transferred naïve B cells (~8uM, Supplementary Videos 15–17). This was consistent with the cells outside GCs being GC B cell phenotype (Extended Data Fig. 2h). Overall, these data show that loss of the Gα13-confinement pathway disperses GC B cells into extra-GC spaces, and that some GC B cells can traffic from one GC to another in this permissive setting.

### De-confined GCs minimally diversify Confetti color palettes

We next took advantage of the Confetti multicolor reporter approach to study GC diversity^6^ in normal and de-confined LNs. When Cre is induced in cells expressing the R26R-Confetti allele, stochastic recombination commits cells to expressing one of four possible fluorescent proteins (nuclear GFP, cytoplasmic YFP, cytoplasmic RFP or membrane CFP); this generates up to 10 color combinations when two alleles are recombined in homozygous mice, though the GFP recombines less efficiently than the other FP-encoding genes^6^. We administered tamoxifen after GC formation (day 5 and 7 post immunization) to induce stochastic color choice in each cell and harvested LNs ~2 weeks later. Having Cre active across the initial days of the GC response is expected to label early expanded clones with multiple colors but preferential expansion of a given lineage can lead to later color dominance. Initial experiments with S1pr2-CreERT2 R26R-Confetti *Arhgef1*−/− mice were unsuccessful likely due to inadequate Cre activity to achieve the recombination events needed to generate the full color palette (Supplementary Fig. 2a). Using Aicda-CreERT2 as in past studies^6,31–33^ we could achieve robust multicolor labeling of GC B cells (Supplementary Fig. 2b). Moreover, using the germline *Arhgef1* null allele rather than a conditional Gα13 allele ensured all GC B cells were knockout. The mice were immunized with non-haptenated protein (chicken gamma globulin) in these experiments to promote a diverse B cell response^6^. We distinguished cells based on strong GFP, YFP, RFP or CFP expression. In control *Arhgef1*+/− Confetti mice, some GCs were dominated by a single color while others retained a mixed color palette (Fig. 2a, b and Supplementary Fig. 3a), in agreement with previous findings showing about one third of GCs undergo clonal bursting in this period^6^. Quantification of color representation and visual inspection across multiple GCs and LNs showed that many GCs in control mice shifted away from the baseline distribution of Confetti colors, which was assessed by flow cytometry in chronic GCs of mLNs^33^ (Fig. 2c, d). Neighboring GCs in control mice also often exhibited distinct color distributions (Fig. 2a). The same analysis on Arhgef1-deficient Confetti reporter mice showed minimal shift of GCs from the baseline color palette (Fig. 2c, d), and all remained poly-colored (Supplementary Fig. 3b). Thus, in LNs with GC dispersal, the color bursting behavior seen in control GCs was lost, and GCs did not develop the distinct color distributions.

To assess whether this result could be consistent with the GC exchange observed by live imaging, we modeled color evolution in Confetti GCs using stochastic simulations with and without exchange (Supplementary Fig. 4a). In Confetti reporter live-imaging, we observed ~1% of GC B cells crossing to other GCs per hour after normalizing for number of cells inside GCs and imaging duration (Extended Data Fig. 2d). This is likely an underestimate due to most crossing cells not being scored because of limitations in live imaging. Thus, we modeled 1-4% exchange per hour as conservative estimates. In the model, GCs peaked at ~day 7 post-onset (corresponding to about day 10-12 post-immunization) and contracted by day 20 post-onset, consistent with GC kinetics after protein immunization^34^ (Supplementary Fig. 4b). B cells exchanged at a speed of 10 um/min between GCs at least 150 um apart. After 15 days of evolution post-onset, simulated GCs displayed similar characteristics as experimental data, with color bursting occurring in the no exchange condition but little divergence from baseline with exchange (Fig. 2e, f). Simulated GCs were observed becoming dominated by a single color over time in the confined setting, and this process was interrupted in LNs with exchange (Fig. 2g and Supplementary Fig. 4c). Overall, the data and modelling suggest that GC exchange disrupts the development of distinct trajectories during B cell clonal evolution.

### GC-dispersed LNs have elevated clonal overlap between poles

Next, we utilized VDJ sequencing to further evaluate GC diversity and exchange. To start, we immunized Gα13^ΔB^ and control mice with NP-KLH and vibratome sectioned live LNs at 300um intervals to obtain pairs of end slices that represented opposite poles of a LN. We reasoned that the ~900um distance separating two end slices would ensure anatomically separate GCs (Fig. 3a). We used bulk heavy chain (Hc) CDR3 sequencing with unique molecular identifiers (UMIs) and initially grouped sequences with identical V, D, and J gene usage and CDR3 amino acid sequence. Repertoire similarity between ends was then computed using Morisita’s index^35,36^,. where higher values represent greater overlap. For control LNs, there was little overlap between LN ends, while Gα13^ΔB^ LNs displayed elevated overlap (Fig. 3b, c and Supplementary Fig. 5). In some Gα13^ΔB^ LNs, large bursts of 1-2 shared CDR3 clones comprised >10% of each LN end (Fig. 3b and Supplementary Fig. 5). To confirm these results, we re-sequenced the samples with full-length VDJ sequencing and constructed clonal lineage trees across LN endpieces using V and J gene assignment, CDR3 length, and junction sequence identity^37,38^ (Fig. 3d and Supplementary Fig. 6). For each tree, we quantified the number of switch events between left and right LN ends among neighboring tree tips, normalized to the number of tree tips. Most clonal trees were confined to one LN end (switch to tip ratio of 0), but a significantly greater fraction of trees were shared across LN ends in Gα13^ΔB^ mice (Fig. 3e).

In a second approach, we generated mixed bone marrow (BM) chimeras with WT BM paired with either CD23Cre+ (WT:control) or *Gna13*f/f CD23Cre+ BM (WT:Gα13^ΔB^). Using fluorescence stereomicroscope guided dissection, we split vibratome sections from immunized LNs at interfollicular regions to generate paired segments with largely separate GCs (Extended Data Fig. 3a). Mixed BM chimeras were used since we reasoned an internal reference would help control for imperfect microdissection. We sorted and sequenced WT and control or Gα13^ΔB^ GC B cells from separated segments and generated clonal phylogenetic trees, similar to the LN endpiece experiment (Extended Data Fig. 3b,c and Supplementary Fig. 7). When using the Morisita index for clonal overlap between LN halves, we found a trend towards higher overlap for Gα13^ΔB^ cells compared to internal WT cells in WT:Gα13^ΔB^ chimeras, but no trend between WT and control cells in WT:control chimeras (Extended Data Fig. 3d). We also conducted a clonal lineage tree switch to tip ratio analysis and found a similar fraction of trees confined to one LN half in WT:control mixed chimeras, but an elevated degree of exchange for trees from Gα13^ΔB^ cells compared to WT in WT:Gα13^ΔB^ chimeras (Extended Data Fig. 3e). Overall, the findings suggest that de-confined GC B cells can transfer to other GCs in a LN and participate in their evolution.

### Spatial VDJ sequencing reveals dissemination of GC clones

To more precisely analyze clone sharing between GCs, we employed ZipSeq spatial sequencing^39^. In this method, vibratome sections are stained with a pan-CD45 antibody conjugated to a caged anchor oligonucleotide, followed by uncaging a region of interest with patterned UV illumination and hybridization of a unique Cy5^+^ ‘zipcode’ oligonucleotide to uncaged anchors. After washing, this process is repeated for other regions. Sections are then dissociated and Cy5^+^ (zipcoded) GC B cells sorted for single-cell VDJ and 5’ RNA sequencing (Extended Data Fig. 4a). We used LN sections from mice immunized with NP-KLH, identifying GC regions by low IgD and reticular CR1/2 (Fig. 4a), which guided local UV illumination.

When overlaying 5’ gene expression on a UMAP representation of cells, almost all cells were of GC B cell phenotype, with a minor offshoot of *sdc1*^+^ (pre-)plasma cells that were filtered from analyses (Extended Data Fig. 4b). Gα13^ΔB^ and control GC B cells were highly similar and did not separate on the Seurat UMAP representation of cells (Extended Data Fig. 4c). Some GC B cells separated from the rest of the population due to *Ighv* genes, and the UMAP clusters collapsed into only one population when *Ighv* genes were removed from consideration (Extended Data Fig. 4d). When analyzing for genes differentially expressed between Gα13^ΔB^ and control cells, there were few that were consistent across three experiments (Extended Data Fig. 4e), consistent with the UMAP overlaps.

For B cell receptor (BCR) VDJ analysis, cells with paired heavy and light chain sequences with unambiguous zipcodes were clonally grouped using the 10x enclone toolkit for lineage tree construction. Trees from clones in control sections often were limited to one anatomic GC, although sharing between adjacent GCs was not infrequent when looking at clonal families across sections (Fig. 4b, c), consistent with previous studies^6,40,41^. In Gα13^ΔB^ mice, trees showed more ‘hopping’ between neighboring GCs (Fig. 4b), and the largest clone was often spread across several GCs (Fig. 4c and Fig. S8a–d). When comparing pairs of GCs with similar separating distances, Gα13^ΔB^ LNs exhibited significantly more clonal families containing cells from both GCs (Fig. 4c and Supplementary Fig. 8a–d). To formally normalize for inter-GC distance in clonal overlap quantification, we computed an overlap enrichment index for all pairs of GCs on consecutive LN sections and plotted against distance (Fig. 4d). A value of 1 for the index indicates the degree of overlap expected by chance. Unless closer than ~250-300 um, most GC pairs had index <1, likely reflecting a biological drive for isolating lineages into separate GCs. However, the decay of the overlap index versus distance was significantly slower for Gα13^ΔB^ compared to control mice (Fig. 4d), indicating a relaxation of the GC confinement constraints on lineage separation. Furthermore, analysis of individual lineage trees from all sections using the switch to tip ratio measure showed greater inter-GC ‘hopping’ along tree topologies for Gα13^ΔB^ mice (Fig. 4e). This was true when looking at number of hops or inter-GC distance-weighted hops (Fig. 4e).

As a further analysis, we measured the Levenshtein distance (LD) from root for each hopping event in a clonal tree (Extended Data Fig. 4f). LD provides a probabilistic estimate of the minimum number of edits from germline required to reach an inferred node or observed sequence (excluding regions of recombination) along different possible mutation paths. Although LD only approximates the number of SHMs, we reasoned that hopping events occurring at nodes with LD>1 would have at least 1 mutation and be more likely to occur at the GC rather than precursor B cell stage. With this filter, the number of hops per node was still significantly greater in Gα13^ΔB^ mice (Extended Data Fig. 4g). The median LD for filtered hops, which was >2, was not significantly different between control and Gα13^ΔB^ mice (Extended Data Fig. 4h). Finally, we also analyzed the position of shared SHMs among clonally related B cells from different GCs (Extended Data Fig. 4i). Among all the pairs of adjacent B cells on lineage trees originating from different GCs and sharing at least one SHM (same nucleotide position and resulting base pair), ~85% and 72% in control and Gα13^ΔB^ mice, respectively, shared at least one SHM in a framework region or at least 2 SHMs in CDR regions (Extended Data Fig. 4j). The higher proportion in control LNs is likely consistent with less frequent and delayed jumping events in the confined setting. Since sharing a framework region SHM or ≥2 CDR SHMs is highly unlikely by chance^42^, this supported most B cell relations across GCs being true clonal groupings. Taken together, these results support the GC hopping behavior suggested by the bulk sequencing data.

### Less diverse B cell repertoire in LNs with de-confined GCs

Another observation from the ZipSeq spatial sequencing was that Gα13^ΔB^ cells displayed a greater fraction of lambda light chain (Lc) clones, which is associated with NP hapten binding^43^, and lower diversity by Simpson’s index, a measure of the likelihood two cells come from the same clone (Extended Data Fig. 4k, l). This suggested that clonal spreading may lead to increased domination by cells binding certain epitopes, such as the accessible NP hapten on NP-KLH. To determine the effects of GC B cell dispersal on diversity, we performed bulk Hc sequencing on NP+ and NP− LN GC B cells from total LNs at day 12 after NP-KLH immunization (Supplementary Fig. 9a). Initially, we sequenced both Hc and Lc and this demonstrated the expected strong skewing of NP+ B cells to lambda Lc usage (Supplementary Fig. 9b). However, the more efficient amplification of Lc versus Hc led to fewer Hc sequence reads (Supplementary Fig. 9b), and we therefore focused on Hc bulk sequencing in subsequent experiments. Hc isotype analysis showed strong enrichment for IgG over IgM in NP+ cells (Supplementary Fig. 9b). Consistent with the ZipSeq data, there was also a somewhat elevated lambda Lc fraction among NP+ GC B cells in de-confined *Arhgef1*−/− LNs versus control (Supplementary Fig. 9b). In control mice, the expected dominant expansion of Vh1-72 with several J elements^44,45^ was observed, with Vh1-53, Vh3-6, and Vh1-64 also being expanded (Fig. 5A). However, in Ga13^ΔB^ mice, larger Vh1-72 expansions were detected and several additional Vh elements (Vh1-64; Vh1-36; Vh10-3; Vh3-6) underwent strong expansions (Fig. 5a). These expanded Vh elements also occupied greater fractions of the total LN repertoire compared to their counterparts in control LNs.

We next clonally grouped Hc sequences as was done for lineage tree construction and analyzed clonal distribution using rank-abundance curves^46^. We observed a significant increase in the fraction of GC B cells dominated by the top several clones, and this effect was seen in both the NPȒ and NP+ populations (Fig. 5b). To compare diversity between LNs, we plotted Hill diversity curves^47^, a unified quantification of diversity where diversity order q=0 measures the number of different species and q>2 measures how dominant the most common species are. Interestingly, the Hill diversity curves showed similar species richness (q=0) between control and Gα13^ΔB^ LNs but significantly lower diversity in Gα13^ΔB^ mice for measures of clonal dominance (q>2) (Fig. 5c). This was consistent with the rank abundance curves showing a disproportionate bias towards top clones. Furthermore, these results were recapitulated for Hc in bulk whole LN GC B cell sequencing of *Arhgef1*−/− versus +/− LNs (Supplementary Fig. 9c, d), despite the lower sequencing depth in these samples due to Lc amplification bias and initial library preparation optimization.

In addition to repertoire distribution, we analyzed SHMs known to promote affinity maturation toward NP. The CDR1 W33L mutation in Vh1-72 sequences is a standalone mutation that confers a 10-fold improvement in NP-binding affinity^44^ and it was enriched in the NP+ sorted GC B fraction compared to the NP− fraction (Fig. 5d). Importantly, this mutation was observed more frequently in Gα13-deficient NP+ Vh1-72 GC B cells compared to control (Fig. 5d), potentially reflecting the imbalanced outgrowth of top NP-binding clones. Another characteristic Vh1-72 mutation is K59R, which typically only improves NP affinity when accompanied by a G99 V-D junction variant and other Hc and Lc mutations. W33L, in contrast, requires Y99 at the first CDR3 junctional residue and does not require additional mutations. As a result, K59R tends to be mutually exclusive with W33L and increases in dominance later during the GC response due to a more complex mutational path^48–51^. At day 12 post-immunization, we observed enrichment for K59R in NP+ over NP− GC B cells in control mice, indicating successful usage of this alternative pathway in a normal response (Fig. 5d). However, opposite to the W33L elevation, K59R was barely enriched and rather suppressed in Gα13^ΔB^ NP+ cells compared to control (Fig. 5d). Similarly, the combination of K59R with the Y99G junction variant was lost from the NP+ population in Gα13^ΔB^ mice (Fig. 5D). We speculate that a greater early dominance by Gα13-deficient W33L mutant cells across GCs hinders the emergence of K59R mutant cells. These results reveal a greater imbalance of clonal representation in GC-dispersed LNs, and this potentially results in early sweeps subduing later appearing clones.

### LNs with dispersed GCs favor immunodominant cells

The total LN repertoire sequencing indicated outgrowth of certain clones, so we sought to characterize the impact of GC mixing on dominant and sub-dominant clones via more direct BCR binding assays. In connection with our NP-KLH sequencing data, we quantitated the frequency of NP binding GC B cells at day 12 post NP-KLH immunization. In highly NP-haptenated protein antigens, NP is the dominant epitope^52^. This likely reflects the ready accessibility of the surface coupled NP groups, the effect of epitope valency on binding strength, and the high (~1/4000) NP-binding B cell precursor frequency^23,53–55^. Gα13^ΔB^ mice had GCs of similar size to WT (Extended Data Fig. 1c) but there was a significant increase in the proportion of GC B cells specific for NP (Fig. 6a). Similar findings were made using a second B cell-specific Cre line (Extended Data Fig. 5a). These data suggested that GC B cell dispersal favored the response to the accessible NP hapten and potentially reduced the frequency of B cells binding the underlying KLH protein carrier.

To assess the protein carrier GC B cell response, we took advantage of the finding that immunization with intermediate NP-haptenated OVA induces GC responses involving NP-binding and OVA-binding B cells^52^, but with bias towards the NP response intact (Fig. 6b). At day 12 following immunization with NP9-OVA, there was a shift in the GC response towards the dominant NP epitope with concomitant further suppression of the subdominant OVA in Gα13^ΔB^ mice (Fig. 6c). Similar findings were made in mice with a different B cell Cre line and in mice lacking the Gα13 effector, Arhgef1 (Extended Data Fig. 5b–d). Using the intensity of NP-PE binding as a proxy for affinity^56,57^, the GC dispersed setting also led to higher affinity NP-binding GC cells (Fig. 6d). Overall GC magnitude as well as memory B cell and PC frequencies after NP-OVA immunization were not altered by Gα13-deficiency (Extended Data Fig. 5e). In accord with prior findings^12,28^, a slight increase in the proportion of LZ GC B cells was observed in Gα13^ΔB^ mice (Extended Data Fig. 5f). However, this shift was similar for NP-binding and OVA-binding GC B cells, suggesting it does not underly the shift in epitope response dominance. Since lambda Lc are associated with NP binding clones, we also analyzed lambda and kappa Lc proportions in the naïve B cell repertoire but found no difference between control and Gα13^ΔB^ mice (Extended Data Fig. 5g). In connection to Gα13-mediated confinement, we additionally analyzed S1pr2-deficient mice, but loss of this GPCR was not sufficient to disperse peripheral LN GC B cells (Extended Data Fig. 6a). S1PR2 deficiency did not cause a shift in the frequency of NP+ versus OVA+ GC B cells (Extended Data Fig. 6b), though it did cause a similar increase in the proportion of LZ cells as observed in Gα13^ΔB^ mice (Extended Data Fig. 6c). This suggested that a slight LZ bias in antigen-specific GC B cells is not sufficient to drive the shift in immunodominance.

Looking across time, we saw that the increased dominance of NP+ cells in Gα13^ΔB^ mice persisted from day 12 to day 21 and 31 after NP9-OVA immunization (Extended Data Fig. 7a, b). However, the NP+ proportion dropped over time, likely reflecting antibody negative feedback from NP-specific antibodies. Interestingly, the suppression of OVA+ GC B cells in dispersed LNs continued through day 21 but disappeared by day 31 (Extended Data Fig. 7a, b). Concurrently, the OVA proportion increased from day 12 to 21, again likely reflecting antibody feedback, but contracture of the GC by day 31 potentially contributes to weaker effect sizes in both the NP and OVA populations (Extended Data Fig. 7c). Serum titers of NP-specific antibody, measured with NP20-BSA as bait, was not different between control and Gα13^ΔB^ mice at day 12 or 21 (Extended Data Fig. 7d). These data indicate that differences in antibody feedback are unlikely to contribute to the differences in day 12 GC B cell diversity. However, affinity to NP, measured using a ratio of binding to NP2-BSA versus NP20-BSA, did increase from day 12 to day 21 and trended towards higher values in Gα13^ΔB^ mice by day 21, but not at day 12 (Extended Data Fig. 7e). OVA antibody titers had a similar lag response, with no difference at day 12 but a reduction in Gα13^ΔB^ mice at day 21 as anti-OVA titers increased (Extended Data Fig. 7f).

We also considered whether the elevated NP+ B cell expansions could occur due to intrinsic effects of Gα13, namely increased proliferation and resistance to apoptosis, as observed in mucosal lymphoid tissue^10,12,28^. For proliferation, we analyzed 2 hour EdU incorporation in popliteal LN GC B cells. EdU+ fractions slightly decreased in Gα13^ΔB^ compared to control (Extended Data Fig. 8a). While contrary to the role of Gα13 in suppressing GC proliferation, this was consistent with elevated LZ (and reduced DZ) in Gα13^ΔB^, as GC B cells primarily proliferate in DZ^15^. It is also consistent with Gα13 proliferation effects being absent in peripheral LN compared to mLN^28^. For cell death, we incubated LN cells with a caspase-3/7 peptide (DEVD) conjugated to a nucleic acid binding dye that can enter the nucleus when liberated by active caspase-3/7. Contrary to prior work^10,12,28^, we found increased caspase activity in Gα13^ΔB^ GC B cells compared to control (Extended Data Fig. 8b). We attribute this difference to previous literature to the shorter incubation time (15 min) needed for our assay, as staining with active caspase-3 antibody after incubation for 3 hours, used in previous work, reproduced the protective apoptosis effect of Gα13 deficiency (Extended Data Fig. 8c). Importantly, both S1pr2 and Gα13 deficiency produced similar increases in caspase activity, making it unlikely to explain the NP selection effects (Extended Data Fig. 8b). MLNs from Gα13^ΔB^ and *S1pr2*^−/−^ mice showed no change in LZ/DZ polarity compared to control, consistent with previous data^28^, and no change in caspase activity (Extended Data Fig. 8d, e).

As another test of GC dispersal effects on selection, we assessed antigen specificities in Gα13^ΔGCB^ and control mice after NP-KLH and NP9-OVA immunization and induced Gα13 deletion at the onset of GC formation. Cre-ERT2^S1pr2^ mediated deletion of Gα13 phenocopied the rise in NP+ B cell populations and trend towards higher LZ in NP+ B cells after NP-KLH immunization (Extended Data Fig. 9a, b). Because the LZ effect was more variable in Gα13^ΔGCB^ mice, we plotted LZ percent among NP+ B cells against NP+ fraction for the Gα13^ΔGCB^ LNs but found no correlation (Extended Data Fig. 9b). In NP9-OVA immunized Gα13^ΔGCB^ mice, we observed concomitant expansion and suppression of NP+ and OVA+ GC B cells similar to Gα13^ΔB^ mice and no correlation between degree of LZ bias and NP+ bias (Extended Data Fig. 9c, d). Gα13^ΔGCB^ mice displayed dispersal of GC B cells to interfollicular regions while tight confinement was maintained in tamoxifen treated control mice (Extended Data Fig. 9e). These results suggest that Gα13 loss at the time of GC formation is sufficient to disseminate GC B cells and skew the response to favor dominant over subdominant clones.

### Shifts in epitope preferences for a complex antigen

For larger protein antigens without a stereotyped epitope such as NP, immunodominance hierarchies and outcomes after GC exchange may be more complex. To investigate this, we immunized mice with influenza hemagglutinin (HA) trimer of the New Caledonia 1999 strain (NC/99). To probe antigen specificity, we utilized ferritin-based nanoparticles displaying either the head or stem portion of NC/99 HA (Fig. 6e)^58,59^. Fluorescent dyes were conjugated to the nanoparticles to distinguish head vs stem specific GC B cells. We expected the head-specific B cells to be immunodominant over stem-specific, which have the potential to produce broadly neutralizing antibody binding conserved sites on the stem^58–60^. Indeed, in control mice, head-binding GC B cells dominated the response at day 12 (Fig. 6f). However, in Gα13^ΔB^ mice, stem-binding B cells unexpectedly expanded while head-binding B cells decreased in frequency (Fig. 6f). In the serum as well, the stem-specific antibody trended toward higher titer at day 12 while head-specific antibody was decreased (Extended Data Fig. 10a). We could not quantitatively compare our head and stem ELISA readings, but by flow cytometry, the PC response at day 12 was mostly head specific in control and Gα13^ΔB^ mice, though with lower head-binding PCs in Gα13^ΔB^ mice (Extended Data Fig. 10b).

While the mechanism of this shift in epitope binding requires further investigation, it may involve antibody feedback. Another possibility is greater access of stem^+^ clones to Tfh cell help due to fewer stem^+^ than head^+^ B cells in the early GC pool^61^, resulting in less competition from clones presenting similar MHCII peptides^62^. In the setting of de-confinement, such effects may allow stem^+^ clones to burst in multiple GCs more effectively than head^+^ clones. Sweeping by a single or handful of stem^+^ clones might also occur more readily in a GC response that does not recruit as many high-affinity precursors as NP does. Compared to NP-OVA, equal accessibility of the head and stem on the elongated soluble HA trimer could aid bursting of stem^+^ B cells, whereas the NP-over-OVA configuration imposes steric barriers to OVA in the presence of NP-specific BCRs or antibodies. Thus, differences in antigen geometry and precursor frequency, both important determinants of immunodominance^61^, likely contribute to different outcomes in responses to HA versus NP-OVA. However, the HA stem^+^ B cell expansion, which appears advantageous in dispersed LNs, may still be detrimental to diversity. Within the stem^+^ B cell population, the percent of cells also binding PR/34 HA tetramer, a related H1 variant, was reduced in Gα13^ΔB^ mice (Fig. 6g), suggesting that more broadly reactive stem-specific B cells are reduced. Like the NP-OVA response, early sweeping clones, which may bind NC/99 specific epitopes higher on the immunodominance hierarchy, could suppress cross-reactive cells on more complex affinity maturation pathways.

## Discussion

Sequencing analysis of GCs in mouse and human has provided evidence for distinct clonal trees in different GCs^40,63,64^. Analyses of clonal bursting provided further evidence of GC B cell confinement^6,31–33^. In this study, we establish that the B cell deconfinement from GCs that occurs with Gα13-pathway deficiency leads to increased exchange between GCs. Under these conditions, there is increased clonal sharing between GCs, a reduced diversity of GC B cells in the responding LN, and greater focusing of the response on an accessible epitope. Piecing these findings together, we believe that clonal sharing allows immunodominant clones, such as NP hapten-specific clones, to outcompete other lineages in multiple arenas, leading to a “winner-takes-all”-type phenomenon. However, one lineage alone cannot sweep the LN, as multiple colors were still observed in the de-confined Confetti reporter mice, and the top clones vying for dominance by sequencing were not limited to the canonical NP-binding Vh1-72 cells. In the setting of more complex protein antigens such as influenza HA, dispersed LNs may allow a single clone to dominate due to less stereotyped precursor binding, leading to a stronger but narrower response to the least masked parts of the antigen. Thus, these findings suggest that GC B cell confinement in separately evolving islands contributes to immune response breadth by tamping a premature spread of winner B cells.

While prior work has provided extensive evidence for independent evolution of B cell clones in separate GCs^6,63–65^, several studies had detected clonal sharing. This has included analysis of cells sorted from paired GCs in responding mouse LNs^6,32^, laser capture microscopy of GCs in reactive human LNs^40^, and spatial sequencing of responding mouse LNs and human tonsil^41,66,67^. Collectively, these studies have provided evidence that ~5-20% of clones are present in multiple GCs. Recently, it was suggested that a ‘goldilocks’ level of GC B cell exchange may enable selection of clones at larger population sizes without hyper-focusing, which could allow ‘winner’ clones to sample more mutational space^41^. Our data provide evidence that increasing the exchange may benefit the organism by quickly expanding high affinity clones, such as W33L bearing Vh1-72 cells. However, this short-term benefit may lead to longer-term disadvantages, as binding to less accessible, conserved parts of an evolving pathogen depends to some degree on the survival of later expanding, sub-dominant clones^16,17,19^. Even affinity for the dominant epitope could flatten out earlier in dispersed LNs. The K59R pathway, for example, has the potential to exceed W33L for NP affinity if allowed to accumulate additional mutations over time^48,49^. However, this evolutionary pathway is stifled in GC-dispersed LNs.

In humans, LNs are often in chains, and several LNs may be involved in the response to an infection or vaccination. Deconfinement and traffic to downstream LNs could allow winner clones to sweep through several hundred GCs, having an even greater negative impact on response diversity. In humans but not mice, P2RY8 cooperates with S1PR2 in promoting GC confinement^14^. It is unclear whether this leads to a stronger confinement effect in human GCs, though spatial sequencing has suggested a similar extent of exchange in mouse and human GCs^41,66,67^. While rodents lack P2RY8, an additional confinement receptor is inferred to operate in mouse GC B cells to explain the stronger deconfinement phenotype observed in Gα13-versus S1PR2-deficient GC B cells.

An alternative explanation for increased Vh clone sharing between GCs could be greater clonal expansion at the activated B cell stage and broader participation across GCs at the time of seeding. While not definitive, our finding of closely related branches in clonal trees originating from different GCs or section segments suggests sharing occurred at a later stage, as also inferred in other studies^6,32,40,41,66,67^. Moreover, the similar phenotypes between S1pr2-CreERT2 and Mb1/Cd23-Cre mediated Gα13 deletion supports GC-stage spreading. Increased representation of the W33L mutant among Vh1-72 sequences is also less likely to result from pre-seeding expansion. Another alternative explanation for increased clone sharing between GCs could be increased generation of memory cells that might occasionally be induced to re-enter GCs soon after exit. Arguing against this notion, we did not detect an increase in memory B cells in Gα13-deficient mice. Lastly, previous work did not observe Gα13-deficient cells outgrowing WT cells in peripheral LNs^28^, consistent with our measurements of GC size. Despite these lines of evidence, we cannot fully exclude that there may be other Gα13-pathway signaling effects not yet tested influencing clonal dynamics.

While the Gα13 and Arhgef1 mutations reduce confinement, they do not abolish it. Increased exchange due to more substantial GC disruption might lead to even further reduction in responding B cell diversity. Certain infections induce disorganized GCs in some settings, including malaria and HIV^68–70^. Moreover, GCs in people with follicular hyperplasia can be very close and in some cases may have fused^71^. It will be of interest to explore how pathogens or other disease processes that affect GC organization impact B cell response diversity.

Overall, we conclude that GC confinement is likely a critical factor in maintaining GC B cell repertoire diversity over the course of a weeks-long response. As antibody feedback exerts pressure on the response to accessible epitopes, clones that were initially less competitive but protected due to confinement may serve as sources of diversity in later stages^26^. However, immunodominant clonal spreading appears to limit the participation of these cells. Furthermore, GC confinement may promote diversity in other ways. For example, maintaining confinement of GCs in bi-lobed LNs draining multiple sites could facilitate responses to several antigens simultaneously. Confinement may also optimize GC B cell encounter with cognate Tfh, which have been observed exchanging between follicles^15^, and simultaneous exchange of both partners could reduce encounter probability. Thus, actively confining B cell evolution to archipelagos within LNs likely presents multiple benefits for the humoral response.

## Materials and Methods

### Animals

BoyJ (CD45.1) and C57BL/6JxBoyJ (CD45.1 and CD45.2) mice were bred in an internal colony and 7–12 week-old mice were used as donor mice for mixed bone marrow (BM) chimeras. *Gna13*^fl/fl 72^, *Arhgef1*^+/− 73^ and Cre^Mb1 74^ were from an internal colony and had been backcrossed to C57BL/6J for at least 10 generations. Cre^Cd23^ mice^75^ and Cre-ERT2^Aid^ *Rosa26*^Confetti/Confetti 6,76^ were kindly provided by G. Victora (Rockefeller University). Cre-ERT2^S1pr2^ mice^57^ were a kind gift from T. Okada (RIKEN). *Rosa26*^Ai14/+^ (Gt(ROSA)26Sor^tm14(CAG-tdTomato)Hze^) were from Jax. *S1pr2*^+/−^ mice^77^ were kindly provided by R. Proia (NIH). To produce mixed chimeras, CD45.1 congenic Boy/J mice were lethally X-ray irradiated with 900 cGy in split doses and reconstituted with 5 × 10^6^ BM cells of a mix of CD45.1/.1 Cre^Cd23^ or CD45.2/.2 *Gna13*^fl/fl^ Cre^Cd23^ and CD45.1/.2 C57BL/6JxBoyJ. Mice were analyzed 6–7 weeks later. Mice were sex matched if possible per experiment, and both male and female mice were used for all experiments. Based on mice availability, approximately equal numbers of mice from each genotype were selected for experiments. Mice were randomly weaned into vivariums before genotyping. Mice were immunized and data were acquired in alternating control versus KO order when possible. All mouse lines used were bred and maintained in our laboratory. Animals were housed in a specific pathogen-free environment maintained at 23 °C with 40–60% humidity on a 12-h light/12-h dark cycle in the Laboratory Animal Resource Center at the University of California, San Francisco, and all experiments conformed to ethical principles and guidelines that were approved by the Institutional Animal Care and Use Committee.

For immunizations, mice were immunized with 20 μg of NP9-OVA or NP29-KLH or CGG precipitated in 50 μL of aluminum hydrogel and PBS (1/3 volume alum) in both hind footpads. For tamoxifen treatments, mice were orally gavaged with tamoxifen dissolved in 9:1 sunflower oil:ethanol. *Rosa26*^Ai14/+^ Cre-ERT2^S1pr2^ mice were treated with 7-10 mg of tamoxifen at 35 mg/mL and Cre-ERT2^Aicda^ *Rosa26*^Confetti/Confetti^ mice were treated with 10-12.5 mg of tamoxifen at 50 mg/mL.

### Flow cytometry and cell sorting

LNs and spleen were processed via macerating through a 100 μm cell strainer in FACS buffer (PBS+2% NBCS+2mM EDTA). Spleens were incubated with ACK lysing buffer for 2 min before staining. For mLNs, samples were collected in complete RPMI (10% FBS, 10 mM HEPES, 2 mM glutamine, 55 μM 2-mercaptoethanol and 50 IU/mL penicillin/streptomycin) on ice and additionally washed with FACS buffer before staining. For flow cytometry staining, cells were placed in a 96-well round bottom plate and washed with staining buffer (PBS containing 2% NBCS and 0.5 mM EDTA), and 25-40 μl of antibody cocktail was added to stain cells at ~10^7^ cells/ml and incubated for 20-45 min on ice. All antibodies were used at 1-2 μg/ml in staining buffer. Anti-mouse CD16/CD32 at 5 μg/ml was included in staining volumes. NP20-PE was used at 5 μg/ml. To stain OVA-specific GC B cells, OVA-tetramer (~3 μM) was made fresh by incubating OVA-biotin with streptavidin-APC at a 4.1:1 molar ratio for 2 hours on ice. OVA-tetramer was then used at a 1:200 dilution for surface BCR staining. CXCR4 was stained using biotinylated clone 2B11 followed by a wash with staining buffer and incubation with fluorophore-conjugated streptavidin at 0.5 μg/ml for 20 min. After incubation, cells were washed twice with staining buffer. Data were acquired using a BD LSR II flow cytometer or a Cytek Aurora. A BD FACSAria II was used to sort GC B cells with a 70 μm or 85 μm nozzle using purity precision mode. Aliquots of post-sorted samples were used to assess purity (>96% per sample). Flow cytometry data were analyzed using Flowjo (v.10.6.2).

### Immunofluorescence microscopy

LNs were fixed in 1.6% paraformaldehyde in saline for 2 hours at 4°C, after which tissues were washed in saline and placed in 30% sucrose at 4°C overnight until LNs were dense enough to sink to the bottom of the sucrose solution. Tissues were washed, dried, and oriented in OCT to acquire both medulla and follicle views with each section of the LN. Cryosections of 7μm were dried for 30 min and then blocked with blocking buffer containing PBS+5% NBCS+0.5% Tween-20+5 μg/mL anti-CD16/32 (BioXCell) for 1 hr at 25°C. Sections were washed by incubating in staining buffer (PBS+1% NBCS+0.1% Tween-20+2.5 μg/mL anti-CD16/32) for 5 min, followed by 2-3 hr incubation with primary antibody (1-2 μg/mL) cocktail at 25°C. Sections were washed in staining buffer and mounted in Fluoromount-G with no.1.5 coverslips. Images were captured with an inverted Zeiss AxioObserver Z1 microscope and 10x air objective. Images were analyzed in Fiji and brightness and contrast settings set to similar levels for all images from the same experiment.

### Explant lymph node two-photon imaging

Explanted immunized popliteal LNs were prepared for imaging via Stereo Discovery V8 microscope (Zeiss) guided dissection of LNs to remove fat without disruption of the capsule. LNs were maintained in RPMI + 2 mM L-glutamine at 37°C bubbled with 95% O_2_/5%CO_2_ (carbogen) till saturation until placement in perfusion chamber. A thin layer of Vetbond was used to affix LNs to the bottom glass surface of a JG-23W/HP Ultra Quiet Imaging Chamber (Warner Instruments), after which the chamber was heated to 37°C and perfusion started. LNs were oriented to have both cortex and medulla in optical cross-sections to avoid having the cortex or medulla fully facing flat downward on the glass. Carbogen-bubbled RPMI + 2 mM L-glutamine was pumped through a 37°C inline heater (Warner Instruments) using a mini-variable flow pump (VWR) before reaching the chamber. A dual-channel temperature controller (Warner Instruments) was used to monitor and adjust the temperature of the fluid near the LNs to ensure an environment of 36-37°C. Flow rates were measured to ensure at least 1.5 ml/min of media perfusion. Vacuum suction strengths were set to maintain enough fluid overlying the LN while maximizing removal of waste metabolites. The LN was left to settle for ~30 min to reduce drift during imaging.

LNs were then imaged with an upright STELLARIS 8 multiphoton microscope (Leica) equipped with a Chameleon Ultra Ti:Sapphire laser (tuneable line), a Chameleon Discovery laser (1040 nm fixed and tuneable line), and a HC IRAPO 25x/1.00 water dipping long working distance objective with NA 1 (Leica). The objective was first positioned over an area of LN with multiple GCs using epifluorescent and brightfield imaging and visualization through the eyepiece. Time-lapse z-stacks were then acquired using bidirectional scanning at 400Hz to cover an XY region of 590x590 μm^2^ with pixel size 0.58x0.58 μm^2^ and 1.04 μs dwell time per pixel. Objective correction collar was manually adjusted to maximize fluorescent signal before beginning each acquisition. For Cre-ERT2^S1pr2^ *Rosa26*^Ai14/+^ mice, 65 μm stacks were acquired with 2.5 μm step size for 45 min with ~38s between stacks and 1040 nm excitation (~0.70 Watts output for about 70 mWatts at sample). Tuneable emission filters were set to 480-525 nM to capture 1040 nm second harmonic and 545-700 nm for tdTomato emission. Both non-descanned detectors (NDDs) were set to digital mode with minimum gain setting. For Cre-ERT2^Aicda^ *Rosa26*^Confetti/Confetti^ mice, similar imaging settings were applied except 3 excitation lines were used, 1040 nm, 850 nm, and 920 nm, to maximize signal from the 4 fluorescent proteins. Powers were set so each laser delivered ~70-100 mW to the sample with the 4 NDDs set to 438-497 nm (CFP and 920 nm second harmonic), 497-526 nm (GFP and 1040 nm second harmonic), 526-573 nm (YFP), and 574-650 nm (RFP). Z-stacks were acquired at 2.5 μm steps for 60 or 65 μm with imaging durations of 45 min or 120 min. All detectors were set to digital mode with minimum gain setting. Imaging analysis is described in the Supplementary Methods.

### Multiphoton imaging of Confetti thick sections

Popliteal LNs from Cre-ERT2^Aicda^ *Rosa26*^Confetti/Confetti^ mice were harvested into PBS+2% 0.22 μm filtered FCS without EDTA on ice and embedded in 2% low melting point agarose in PBS. LNs were oriented in the agarose to include both cortex and medulla with each section. Excess agarose was trimmed with Leica VT1000 S vibratome and LNs sectioned at 200 μm intervals. Each immunized LN yielded 7-9 sections, and sections were washed with 0.22 μm filtered sterile PBS to minimize NDD overloading during imaging from debris on sections. Sections were immobilized onto glass slides with Vetbond on the agarose rim and a ring of vacuum grease was laid around the section and filled with filtered PBS for imaging with an upright water dipping 2-photon objective as detailed above. Micro-bubbles on sections sometimes obscured GC regions, and if possible, were carefully removed with P10 tips before imaging.

Thick sections were imaged with 10% overlapping z-stacked tiles with the multiphoton microscope used for explant LN imaging. Each tile was 521x521 μm^2^ with each pixel 0.51x0.51 μm^2^. Z-stacks were ~190-200 μm thick with 2.5 μm step size. Images were acquired with bidirectional scanning at 400 Hz, 1.04 μs dwell time/pixel, line average of 2, and correctional collar adjusted as above. Three excitation laser lines, 1040 nm, 850 nm, and 920 nm, were used with about 90-120 mW of power per line on the sample. Tuneable emission filters were set to 453-495 nm (photon counting mode), 498-510 nm (photon counting mode), 521-560 nm (digital mode, minimum gain), 590-650 nm (digital mode, minimum gain). Tiled z-stacks were merged in Leica software with smooth setting for overlap blending before analysis in Imaris. Imaging analysis is described in the Supplementary Methods.

### In-silico Confetti GC simulations

We extended a previously established GC model (DisseD) ^78^ to permit exchange of individual B cells between GC instances. A full description of the model is provided in the Supplementary Information “Germinal Centre Simulation Model”. Briefly, GCs are seeded by a pool of founder cells that, after completing an initial proliferative program in the DZ , migrate to the LZ , where they compete for antigen capture from follicular dendritic cells (FDCs) and for Tfh cell help. The time integrated signal accumulated during a LZ passage determines cell fate. Cells committed to recycling undergo additional iterative rounds of division, mutation, and selection. Somatic hypermutations shift the cell’s position in shape space, thereby altering its affinity. Mutation rates have been previously calibrated assuming an average BCR length of ~700 nucleotides^79^. Transmigration events to a different instance of the GC simulation are triggered probabilistically every 54 s, according to the assumed per cell exchange rate, for B cells that are not undergoing apoptosis and are not engaged with a FDC or Tfh cell. Transmigration is restricted to GCs within the same LN, with each LN comprising eight GCs. Spatially, GCs within a LN are arranged on a spherical shell of radius 500 μm, with a minimum inter-GC distance of 150 μm between GC borders (GC radius = 160 μm). The destination GC for each transmigrating B cell is selected uniformly at random, and migration is assumed to proceed along the shortest path between GC borders at a speed of 10 μm/min^80^. Cellular state variables are assumed to remain unchanged during transmigration. Upon arrival, transmigrated B cells are placed uniformly at random on border grid points of the corresponding target zone. For the in-silico Confetti experiment^81^, each GC B cell is assigned one of four colors (or left unlabelled) using empirically estimated staining probabilities. Labels are maintained throughout the simulation, including during transmigration, and are passed on to daughter cells upon cell division. Color contributions per GC were quantified on various timepoints after GC initiation.

### Isolation of opposite lymph node poles and section halves

For sequencing of LN poles, popliteal LNs were harvested into PBS+2% FCS without EDTA on ice and embedded in 2% agarose in PBS. LNs were oriented in the agarose with a short axis aimed as a normal vector to the sectioning plane such that each section contained both cortex and medulla. Excess agarose was trimmed in the Leica VT1000 S vibratome until the microtome blade was near the LN, at which point the blade position step size was lowered to 80-100 μm until a portion of the LN capsule was sliced. The LN was then sectioned at 300 μm intervals until the last slice, which usually contained a quarter to a third portion of agarose underneath the end of the LN. Each immunized LN yielded 5-7 sections, and the first and last one to two sections were processed for sorting and sequencing in case an endpiece yielded insufficient GC B cells for analysis. Sections were carefully dissected away from surrounding agarose before dissociating through 100 μm cell strainers in FACS buffer. Stained samples were resuspended in 200 μL of FACS buffer for sorting and for all samples the entire volume was sorted at ~1000 events/s with a 70 μm nozzle (>97% sort efficiency) until event rates dropped. Cells were centrifuged at 500 r.c.f. for 5 min at 4°C in a fixed-angle centrifuge before aspiration of supernatant and snap freezing in liquid nitrogen. During analysis steps, all pairs of sections chosen had 3 intervening slices, or 900 μm, between them for consistency.

For sequencing of microdissected section halves, LNs were vibratome sectioned as described above but at 350 μm thickness. After sectioning, slices were stained with 2 μg/ml anti-IgD AF488 and anti-CR1/2 (CD35/21) PE for 1 hr on ice in PBS+2% FCS without EDTA and washed three times with PBS. Sections were then imaged on a Leica MZ16 F fluorescence stereomicroscope with a DFC310 FX digital color fluorescence camera on the high sensitivity binning mode. Brightfield with GFP (470/40 nm excitation filter and 525/50 nm emission filter) and PE (545/30 nm excitation filter and 620/60 nm emission filter) fluorescence overlay was used to guide manual dissection of sections into two pieces with 1-3 GCs per half. A feather steel razor blade was used to cut sections in half, and images were taken before and after cutting to check minimal disturbance of cells in the section. Left and right halves of sections were processed separately, stained for GC and CD45 congenic markers, and sorted as described above to maximize cell yield for small cell number samples.

### Bulk GC B cell BCR library construction and sequencing

For *Arhgef1* and some *Gna13* mouse samples, total RNA from sorted NP+ and NP− GC B cells from whole popliteal LNs was extracted using a RNeasy kit (Qiagen). NEBNext Immune Sequencing kit was then used to reverse transcribe full-length mRNA with a biotin-labeled poly-dT RT primer and tag molecules with a template switching oligo and UMI barcodes, which facilitate PCR error correction and clonotype quantification during analysis. Libraries were purified with streptavidin magnetic beads and primary amplification of Ig light and heavy chain (*Arhgef1*) or heavy chain only (*Gna13*) constant and VDJ regions was performed. Due to lower cell numbers from popliteal LNs and potentially lower BCR transcript in GC compared to follicular B cells, cycle numbers for the BCR-specific first PCR (20-22 cycles used) and Illumina adaptor-specific second PCR (14-16 cycles used) were modified from manufacturer protocol. Cycle numbers were optimized by testing a series of cycle numbers on aliquots from additional samples and visualizing each aliquot on a Bioanalyzer to maximize specific product over background. SPRIselect bead purified libraries were then analyzed on Bioanalyzer DNA High Sensitivity chips for 650-680 bp peaks and quantified using qPCR-based KAPA Library Quantification kits. Sequencing of equimolar pooled samples was performed on an Illumina MiSeq using 600-cycle V3 MiSeq reagent kis with 20% PhiX spike-in and 325 bp for Read 1 and 275 bp for Read 2 and 8 bp for both index reads. Loading concentration and cluster density was optimized based on previous runs with KAPA-quantified additional test samples.

For other *Gna13* mouse samples, Takara SMART-seq mouse BCR with UMIs kit was used to amplify heavy chains only from total RNA extracted with RNeasy. UMI oligos were attached during first-strand RT cDNA synthesis using a template switching oligo followed by two rounds of VDJ and constant region specific amplifications that also add the Illumina adapters and dual indices. Cycle numbers for the first and second PCR were optimized as described above using Bioanalyzer visualization of a series of aliquots from test samples. For total popliteal LN GC B cell samples, 18 and 20 cycles were used for PCR 1 and 2, respectively, instead of the recommended 16 and 20. For LN end slices, samples were grouped into low (< 5,000 cells), medium (5,000-10,000 cells), and high (>10,000 cells) GC cell number samples. Cycle numbers were 22 and 26, 17 and 23, and 17 and 21, for those groups, respectively. For samples from microdissected halves of LN sections, samples were grouped similarly into low (500-1,500 cells), medium (2,000-8,000 cells), and high (>10,000 cells) categories and cycle numbers used were 28 and 28, 22-24 and 26, and 18 and 22, respectively. Amplified libraries were then purified by two rounds of immobilization on NucleoMag NGS Clean-up and Size Select beads. All libraries were analyzed on Bioanalyzer DNA High Sensitivity chips for sufficient signal to background of the ~680-690 bp peaks, especially for low cell number samples, and quantified with KAPA Library Quantification kits before equimolar pooling. Whole LN *Gna13* samples were run on Illumina MiSeq as described above. LN end slices were run on NovaSeq X (libraries quantified with digital qPCR for NovaSeq X runs) with 20% PhiX spike-in using a 10B 300 cycle reagent kit (using one lane of 8-lane flow cell) and 150 bp paired-end and 8bp index reads. To capture full-length VDJ for lineage tree analysis, the LN end slices were also re-run with a NovaSeq X 1.5B 600 cycle kit for 300 bp paired-end reads. One experiment of microdissected LN halves samples was run on the Illumina MiSeq i100 with 20% PhiX with 25M 600 cycle reagent kit and 300 bp paired-end reads, and another experiment was run on NovaSeq X with 20% PhiX and 150 bp paired-end reads. Sequencing analysis is described in the Supplementary Methods.

### Zipcode spatial VDJ sequencing

Popliteal LNs were harvested into cold PBS+2% FCS and embedded while live in 2% agarose. Using a Leica Vibratome or Precisionary VF 310-0Z Vibratome, the LNs were sectioned into 180 μm thick slices and affixed to a LabTek chamber slide using Vetbond applied to the agarose rim. Sections were then incubated with anti-mouse IgD FITC and anti-mouse CR1/2 PE along with the anti-CD45 (30-F11) conjugated to an anchor strand hybridized to a NPOM-caged overhang strand as described in ^39^, with the exception of the internal Cy5 fluorophore, in PBS+2% FCS for 1 h at 4 °C. Following three washes with phenol red-free RPMI+1 mM EDTA+1% FCS, the sections were imaged on a customized widefield microscope and ROI delineated and illuminated with pulses of 365-nm light for a 1 second duration. Zipcode sequences were based on ^39^ and were modified for 5’ capture through a TSO complementary strand and a 3’ Cy5 modificiation. The first zipcode was added at a 1 μM concentration in RPMI and allowed to incubate 10 min. Following three washes, sections were incubated for 5 min with a blocking strand at 0.25 μM in RPMI to prevent residual zipcode strand from binding in the following cycle. Following a series of three washes, the process was repeated for the second zipcode and the cycle repeated until six zipcodes were applied per batch of sections. Following a final blocking step, the section was carefully dissected away from the supporting agarose and dissociated through a 100-μm nylon strainer. Cells were stained for GC markers and sorted for live-dead negative, Cy5+ GC B cells. Sorted cells were washed with cold PBS + 0.04% BSA before encapsulation.

Imaging and photo-uncaging was performed with a customized Zeiss Axiovert 200M inverted microscope fitted with a Mosaic digital micromirror device (DMD) from Andor. Spatially directed photo-uncaging is accomplished through directing light from a mercury arc lamp onto a Mosaic DMD with an 800 × 600 micromirror array in plane with the sample. The sample can be simultaneously imaged using epi-fluorescent excitation. In the imaging software, a user-defined ROI is converted into a mask, which is reflected in the micromirror array. This spatially patterned light is then directed through the microscope and objective onto the sample with ~5 μm resolution in our system. Illumination for widefield imaging was provided by a Lambda DG-4. For photo-uncaging, light from a mercury arc lamp was passed through a 365-nm bandpass filter directed into the DMD. Imaging was performed with a 20x Zeiss objective (Plan-Neofluar) and Photometrics Evolve 512 EMCCD camera. MetaMorph (Molecular Devices) and a custom Visual Basics program was used to control the microscope and to provide a user interface for manual ROI delineation for illumination by the Mosaic DMD across multiple fields of view.

### Zipcode single-cell library construction

After sorting of Cy5+ zipcode labeled GC B cells, the cells were encapsulated following 10× Genomics guidelines using a 5’ v2 dual index kit with a target cell number of ~3000-8000 depending on sort yield. Single cell cDNA and VDJ library construction was then performed as directed by 10× Genomics using v.2 chemistry. Three separate libraries were constructed for each sample: a gene expression, VDJ, and zipcode library. Following the ECCITE-seq protocol^82^, an additive primer (partial Read 2 small RNA) was spiked into the cDNA amplification reaction. During the post-cDNA amplification SPRI cleanup step, given that zipcode reads are significantly shorter (<200 bp), these reads were separated from cDNA and VDJ reads by decanting the supernatant. cDNA reads bound to SPRI beads were processed for VDJ amplification and gene expression library construction as recommended in the 10× Genomics v.2 user guide. Meanwhile the supernatant containing zipcode reads was saved and underwent two successive 3× SPRI cleanup steps. This library was then amplified using primers TruSeq small RNA REV with i7 sequence and SI-PCR primer with i5 sequence (see Supplementary Table 1). Following fragment analysis on the BioAnalyzer and library quantification by quantitative PCR, the zipcode library (175 bp average size) was mixed with the associated BCR VDJ (680 bp) and cDNA (450 bp) libraries at a 2:5:10 molar ratio, respectively, and sequenced on Illumina NovaSeqX with a 10B 100 cycle reagent kit (using one lane of 8-lane flow cell), aiming for a sequencing depth of 2000, 5000, and 10000 reads per cell for ADT, BCR, GEX libraries respectively, using 26 bp Read 1 (captures cell-associated barcode and UMI), 90 bp Read2 (captures VDJ, 5’ transcript ends, or zipcode), and 10 bp each i7 and i5 indices. De novo assembly is used later in analysis to stitch together VDJ read fragments into contigs for each observed gel bead-in-emulsion barcode. Sequencing analysis is described in the Supplementary Methods and Supplementary Table 2.

### Nitrophenyl haptenation and biotinylation of ovalbumin

NP was haptenated to OVA as previously described ^52^. Briefly, NP-OSu linker (LGC Biosearch) dissolved in DMSO (100mM) was mixed at various molar ratios (1:1 to 10:1 of linker:OVA) with OVA dissolved in carbonate buffer (pH 8.0, 10mg/mL). Linker and OVA were rotated overnight at 25°C followed by 3 rounds of dialysis with stirring against PBS with 10 kDa SnakeSkin dialysis membrane (Thermo) in a 4°C cold room to remove excess linker. Hapten ratio was measured by determining protein concentration with Pierce BCA Protein Assay (Thermo) and NP concentration using absorbance at 430nm and extinction coefficient 1.38 M^−1^cm^−1^ and path length 0.5 cm in the plate reader.

OVA was biotinylated to a ratio of 1 biotin molecule per OVA as previously described ^52^. Sulfo-NHS-spacer-biotin linker (Thermo) prepared in ultrapure water was mixed with OVA dissolved in PBS to 5 mg/mL at a 6:1 linker to OVA protein molar ratio on ice for 2 hrs without agitation. The reaction volume was then dialyzed with stirring 3 times against PBS with 10 kDa SnakeSkin dialysis membrane (Thermo) at 25°C to remove unconjugated biotin. The final OVA concentration was measured by Pierce BCA Protein Assay and biotin incorporation using the Pierce Biotin Quantification Kit microplate method. Conjugates with a 1-1.1 biotin per OVA ratio were used for tetramer preparation with streptavidin.

### EdU incorporation and caspase activity measurement

EdU (Sigma) was resuspended in 10 mg/mL PBS with 37C incubation and rotation. Mice were injected retro-orbitally with 2 mg of EdU (or PBS vehicle) 2 hrs prior to harvest. LN cells were processed and surface stained as described above in buffers without EDTA, followed by fixation, permeabilization, and Click-iT labelling of EdU with A647-picolyl azide according to manufacturer instructions (Thermo). Naïve B cells and GC B cells from vehicle injected mice were used as negative controls. For caspase activity measurement, cells were surface stained before incubation with 500 nM CellEvent Caspase-3/7 Green Detection Reagent (Thermo) at 1-10 million cells/mL in PBS + 2% BSA at 37C with periodic mixing. Incubation time was optimized to achieve a clear active caspase+ population without excess in-vitro perturbation cell phenotypes and surface staining. Minimal caspase activity signal was observed with 25°C incubation. During the final 5 mins of staining, 1 μM of SYTOX AADvanced Dead Cell Stain was added to stain necrotic cells, which were gated out of the apoptotic cell measurements. Samples were analyzed without washing or fixing. For direct antibody staining of active caspase-3, harvested cells were incubated for 3 hrs at 37C in complete RPMI in a 5% CO_2_ cell culture incubator. After incubation, cells were surface stained and fixed and permeabilized according to manufacturer instructions with BD Cytofix/Cytoperm and BD Perm/Wash. Cells were stained with anti-caspase-3 clone C92-605 (BD Biosciences) for 30 mins at 25°C before washing and analysis.

### Construction of HA nanoparticle probes

The ferritin nanoparticles used to present the trimeric head or stem domains from NC99 (often referred to as RBD-np and H1ssF, respectively) were generated according to established protocols^58,59,83,84^. Expression vectors encoding the constructs were transfected into Expi293 cells using the ExpiFectamine^™^ 293 Transfection Kit (Thermo Fisher Scientific). Following five days of culture the supernatants were obtained, filtered (VacuCap 8/0.2 μm filters, Pall Corporation) and then exchanged to PBS via tangential flow filtration [Pall Corporation; 10kDa filter (Cytiva, OS010T12)]. The HA particles were bound to Erythrina cristagalli Gel-ECA-Immobilized Lectin (EY Laboratories) and then eluted in 0.2M lactose after a 1x PBS wash. The naked ferritin particles were his-tagged and therefore purified by Ni Sepharose affinity chromatography (20 mM imidazole wash followed by 500 mM imidazole elution). All particles were subjected to further purification size exclusion chromatography (SEC) using a Superose 6 10/300 column in PBS. The B cell probes were generated by amine-reactive labeling (AF594, AF488 or AF647) and then repurified by SEC.

### Influenza HA antigen-specific B cell staining

5 μg of influenza NC/99 trimer precipitated in aluminum hydrogel as described above was injected per footpad 12 days prior to harvest. After surface marker staining, antigen-specific B cells were stained using naked ferritin particles AF594 (as specificity control to exclude background), NC/99 head nanoparticles AF488, and NC/99 stem nanoparticles AF647 with 0.25 μg of probe / 100 μl staining mix, in addition to 20 nM PR/34 HA streptavidin tetramer, on ice. HA tetramers were made by incubating biotinylated HA trimer (Y98F mutant in sialic acid binding site) with streptavidin at a ratio of 4:1 at 25°C, with streptavidin added in quarter fractions every 15 mins. Before use, free streptavidin binding sites were quenched with 5-fold molar excess of D-biotin.

### Plasma ELISA analysis

Mouse plasma was harvested at various timepoints post-immunization using retro-orbital bleeds with heparin coated capillary tubes (Fisher) into EDTA treated tubes (Fisher). Blood was spun at 2,000 x g for 10 min at 4C and supernatant used for analysis. High binding standard ELISA microplates (Grenier Bio-One) were incubated with 5 μg/mL of antigen in carbonate buffer, pH 9.5 overnight at 4C. Plates were washed 3 times with wash buffer (PBS + 0.05% Tween-20) before incubation with blocking buffer (PBS + 2.5% BSA) for 30 min at 25°C. Plasma samples were diluted with blocking buffer at 3 different dilutions. A mix of all samples using 4 μL per sample and total volume 1000 μL blocking buffer was serially diluted two-fold 23 times to create a pseudo-standard curve. 100 μL of diluted plasma was incubated in antigen-coated wells for 2 hrs at 37C, followed by washing 3 times with wash buffer. 100 μl of goat anti-mouse IgG-HRP (Southern Biotech) diluted in blocking buffer (1:5000) was then added and incubated at 25°C for 1 hr. Plates were then washed 3 times with wash buffer, and 75 ul of Ultra TMB substrate (Thermo) was added per well. Plates were agitated at 25°C, and the reaction was quenched with 2 N sulfuric acid when blue color developed after several mins. The plate was immediately read for OD at 450nm, and the 2x serial dilution of the sample mix was used to determine the linear range of OD values and which dilution to use for each sample.

### Quantification and statistical analysis

Prism software (GraphPad 9.0.1) was used for all statistical analyses. The statistical tests used are specified in the figure legends. Two-tailed unpaired t-tests were performed when comparing only two groups, or a log-normal Welch’s t-test if data was sampled from a reciprocal normal distribution, and paired t-tests were used to compare internally controlled groups. The standard deviation, or geometric standard deviation for log-normal Welch’s t-test, was assumed to be equal between the two groups. Two-tailed Kolmogorov-Smirnov tests were used to compare distributions. P value < 0.05 was considered significant. No statistical methods were used to predetermine sample sizes, but our sample sizes are similar to those reported in previous publications^85,86^. Animals that died before the experimental endpoint were excluded from analyses. Data distributions were assumed to be normal, but this was not formally tested. Data collection and analyses were not performed blind to the conditions of the experiments. In summary graphs, horizontal lines are means and all error bars are standard deviation unless indicated otherwise.

## Extended Data

**Extended Data Figure 1.**
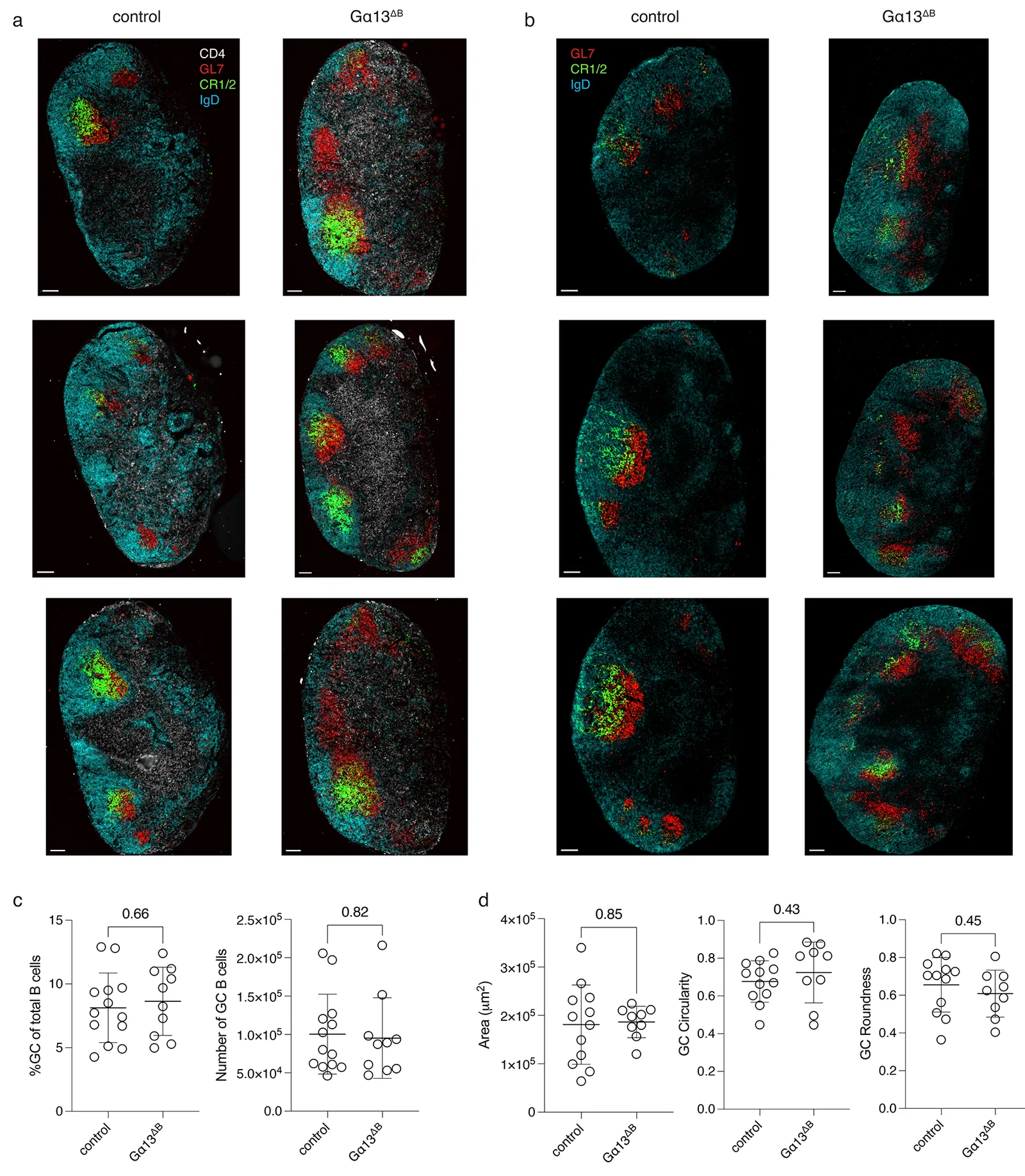
WT and Gα13^ΔB^ LNs display similar size GCs. (A) Additional thin IF cryosections (7 μm) of popliteal LNs day 12 post NP-KLH in alum immunization from *Gna13*^fl/+^ Cre^Cd23^ or *Gna13*^fl/fl^ Cre^Cd23^ mice. Scale bars 100 μm. (B) Representative sections from another pair of *Gna13*^fl/+^ Cre^Cd23^ or *Gna13*^fl/fl^ Cre^Cd23^ mice. Scale bars 100 μm. (C) GC percent of total B cells and absolute number of GC B cells for control (*Gna13*^fl/+^ Cre^Cd23^) and Gα13^ΔB^ mice (*Gna13*^fl/fl^ Cre^Cd23^) at day 12 post NP-KLH in alum immunization. n = 7 control, 5 Gα13^ΔB^ mice; three independent experiments. (D) Quantification of GC area (left), circularity (middle), and roundness (right) for GCs from cryosections of control and Gα13^ΔB^ mice. Largest cross-section was taken from serial slices of each unique GC. Circularity (regularity of edges) and roundness (degree of elongation) range from 0 to 1, with 1 being a perfect circle. n = 4 control, 3 Gα13^ΔB^ mice; three independent experiments. Each symbol represents a LN in (C) or GC in (D). Statistical analysis was performed using two-tailed unpaired student’s t-test. All graphs display mean ± s.d.

**Extended Data Figure 2.**
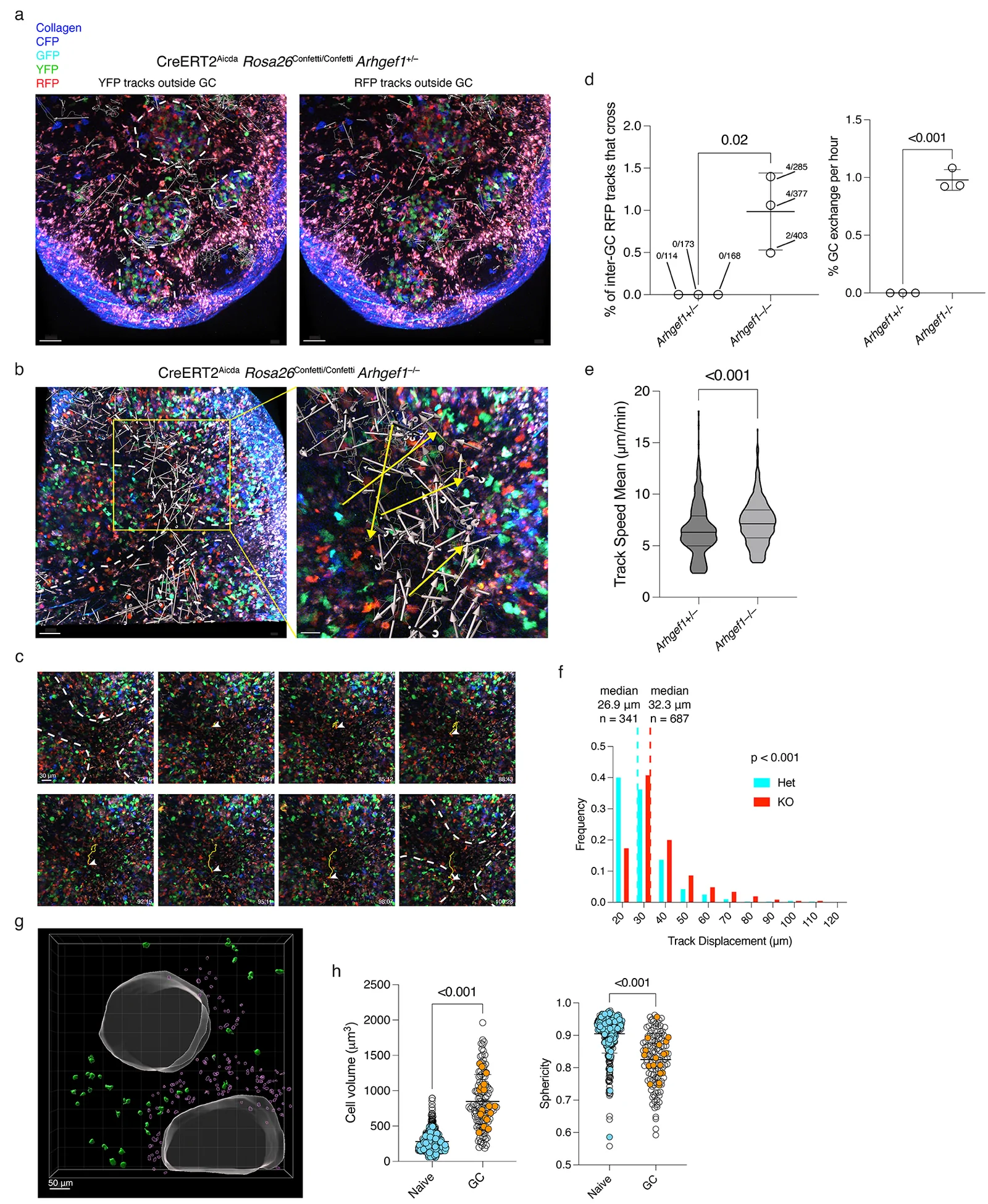
GC B cells can migrate between GCs in the setting of Arhgef1 deficiency. (A) Overview two-photon stack of an explanted popliteal LN from an *Arhgef1*^+/−^ Cre-ERT2^Aicda^ *Rosa26*^Confetti/Confetti^ mouse treated with tamoxifen at day 10 post-immunization and imaged at day 12. Displacement vectors in gray show the start and end positions of YFP+ (left) or RFP+ (right) GC B cells tracked outside the main GC aggregates. Thin gray lines show track paths. White dashed borders highlight GC regions. Additional GC B cell tracking in Movies S7–S12. Scale bar 50 μm. (B) Overview stack (left) as in (A) for RFP+ cell tracks from an *Arhgef1*^−/−^ Cre-ERT2^Aicda^ *Rosa26*^Confetti/Confetti^ mouse. Yellow box highlights inset (right) in which four tracks (yellow displacement vectors) were identified as crossing from center main GC to two GCs on the right. Scale bar 50 μm (left) or 20 μm (right). (C) Time course snapshots of one of the crossing tracks in (B). Time in min:sec indicated on bottom right. Trailing track in yellow displays the last 820 sec**.** White arrowhead indicates cell body. White dashed borders highlight GC regions. Scale bar 30 μm. (D) Quantification of the number of RFP+ inter-GC crossing tracks out of total RFP+ cell tracks outside the GCs (left), displayed as in Fig. 1E. Raw fraction values indicated for each datapoint. The number of crossing tracks was divided by the number of RFP+ GC B cells inside GC aggregates, with percent exchange normalized for imaging duration (45 min or 116 min) to estimate percent of GC B cells crossing per hr (right). Each datapoint represents a LN. Graphs display mean ± s.d. (E) Track speed means for RFP+ cell tracks outside the GCs from LNs of *Arhgef1*^+/−^ or *Arhgef1*^−/−^ confetti reporter mice. Violin plot lines indicate median and quartiles. (F) Histogram of net displacement distances of tracks from (E). Histogram bin centers indicated on x-axis. Median displacement values indicated with dashed lines. In (D-F), n = 3 *Arhgef1*^+/−^, 3 *Arhgef1*^−/−^ mice; three independent experiments. (G) Example plot from Movie S16 of surface contours of Confetti reporter+ cells (green) and transferred CellTracker Deep Red labelled naïve lymphocytes (purple) outside GCs (gray) in an *Arhgef1*^−/−^ confetti reporter mouse (day 13 post immunization, tamoxifen treated day 10, naïve cell transfer day 12). (H) Quantified cell volumes (left) and sphericities (right) of transferred naïve lymphocytes (305 cells) and endogenous Confetti reporter+ B cells (113 cells) outside the main GC clusters (white datapoints) using contours as in (G). Data pooled from two experiments with *Arhgef1*^−/−^ confetti reporter mice. Blue datapoints indicate volumes of dye labelled purified naïve B cells (142 cells) from a separate experiment (Movie S17) for reference. Orange datapoints are crossing GC B cells from Ai14 and Confetti reporter imaging (Movies S1–S12). Graphs display mean ± s.d. n = 2 *Arhgef1*^+/−^, 2 *Arhgef1*^−/−^ mice; two independent experiments. Statistical analysis was performed using two-tailed unpaired student’s t-test (D) or two-tailed Kolmogorov-Smirnov test (E-F, H).

**Extended Data Figure 3.**
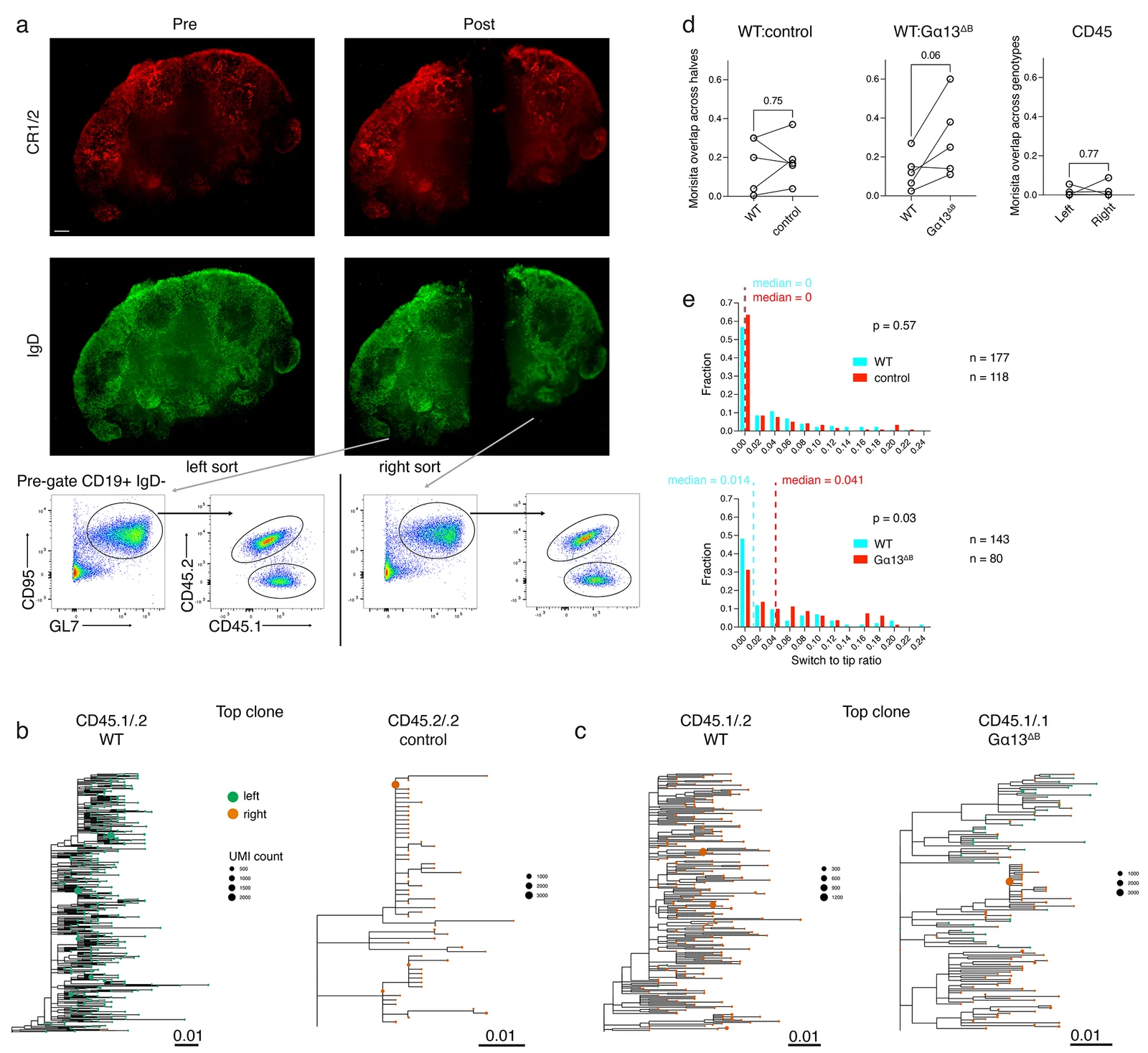
GC B cell clonal relation across adjacent LN halves increases with GC dispersal. (A) Example 350 μm popliteal LN vibratome slice from a WT:*Gna13*^fl/fl^ Cre^Cd23^ mixed chimera at day 20 post NP-KLH immunization that was used for stereomicroscope-guided dissection into two halves containing largely separate GCs. Sections were stained with CR1/2 (red) and IgD (green) to identify FDC networks and GC regions. Pre- and post-cut images of the section shown. Each half of the micro-dissected section was sorted for GC B cells and split into WT or Gα13^ΔB^ populations based on CD45 congenic markers. Scale bar 100 μm. (B) Phylogenetic tree of the largest clonotype from the CD45.1/.2 WT or CD45.2/.2 *Gna13*^+/+^ Cre^Cd23^ group of a WT:control mixed chimera LN section. Observed sequences at tree tips color coded by side of origin (left or right half) and scaled by UMI count. Scale bar of branch lengths indicates number of substitutions per nucleotide. (C) Phylogenetic tree as in (B) for a WT:Gα13^ΔB^ mixed chimera LN section. Additional trees in Supplementary Fig. 7. (D) Morisita’s overlap of repertoire similarity between adjacent halves of each LN section from WT:control (left) and WT:Gα13^ΔB^ (middle) mixed chimeras and, as a further control, between genotypes (defined by the CD45 alleles) in left and right half sections of WT:Gα13^ΔB^ chimeras (right). CDR3 amino acid sequences and IGHV gene usage were used to determine repertoire overlap. Each pair of connected points represents a LN section. Data pooled from two independent experiments. For data using 300 bp paired-end sequencing, 0.5-10×10^3^ cells per genotype per section half were sequenced with 1-2×10^6^ read pairs per sample, and each genotype per section (left and right halves combined) yielded 100-600 clonal groups with 2-12 groups accounting for 50% of the population. For data using 150 bp paired-end sequencing, 0.5-10×10^3^ cells per genotype per section half were sequenced with 20-30×10^6^ read pairs per sample, and each genotype per section yielded 300-500 clonal groups with 4-10 groups accounting for 50% of the population. Statistical analysis was performed using two-tailed paired student’s t-test. (E) Quantification of switch to tip ratio, similar to Fig. 3E, for all clonotype families from sections. Median ratio indicated with dotted lines and number of clonotype families analyzed indicated on the right. Statistical analysis was performed with two-tailed Kolmogorov-Smirnov test. In (D-E), n = 2 WT:control mice, 2 WT:Gα13^ΔB^ mice; two independent experiments.

**Extended Data Figure 4.**
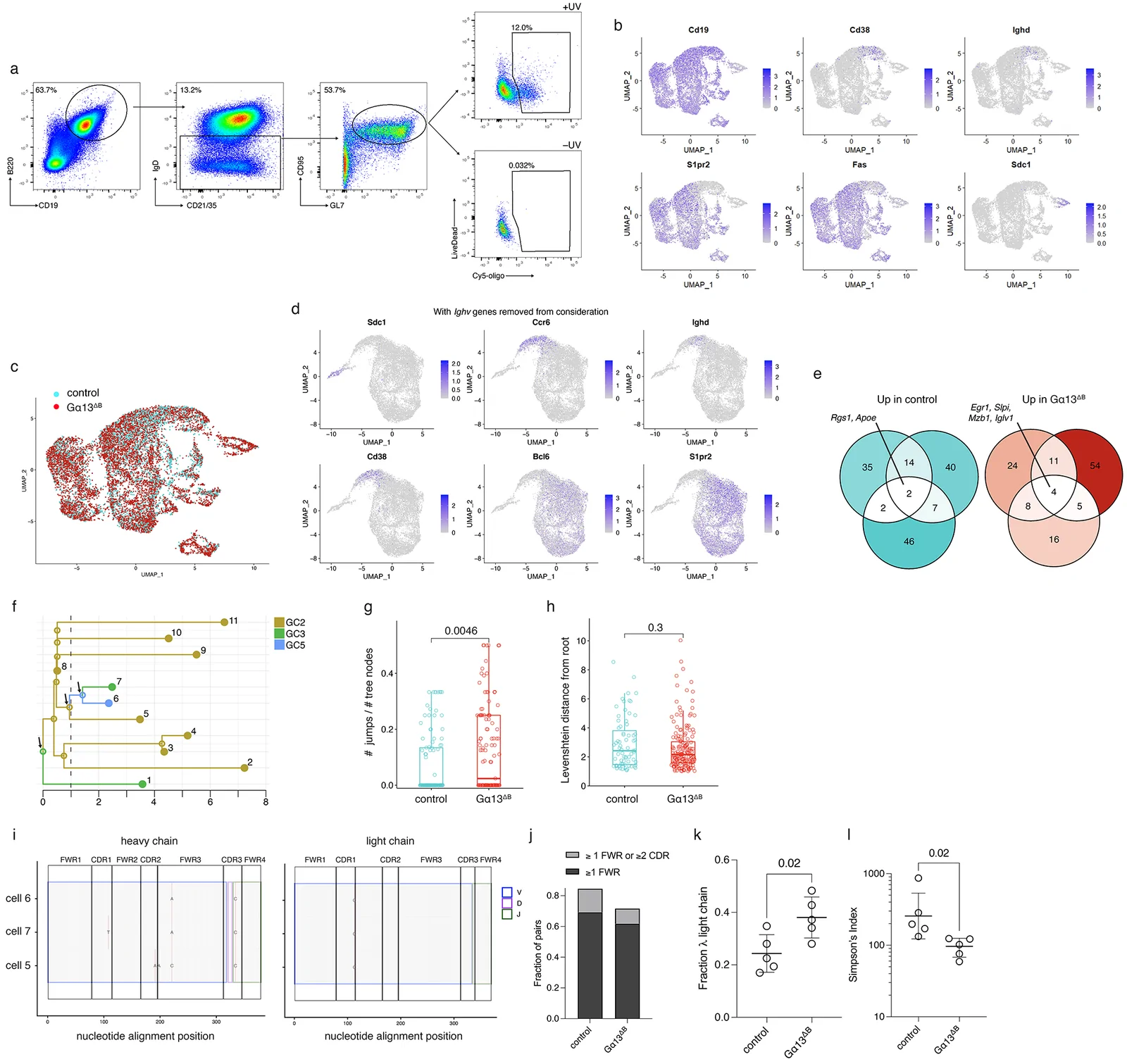
Additional analyses of single-cell ZipSeq BCR sequences and gene expression. (A) Example sort gating from a LN vibratome section after imaging and GC barcoding. GC B cells that were successfully tagged after spatial UV exposure are positive for a Cy5-labelled zipcode oligo. No UV exposed sample shown as negative control. (B) Expression of select GC B cell relevant genes overlaid on UMAP plot of cells sorted for single-cell ZipSeq. A minor population of T cells and myeloid cells were filtered out before clustering. An offshoot of *Sdc1* expressing cells, likely plasma cells and pre-plasma cells, was present and filtered out for ZipSeq analyses. (C) Overlay of genotype information on UMAP plots from (B). (D) Re-clustering of cells in (B) with *Ighv* genes removed from consideration. (E) Overlap of genes significantly upregulated in WT or Gα13^ΔB^ GC B cells from 3 independent ZipSeq experiments. The genes overlapping for all 3 experiments are indicated. (F) Example ZipSeq lineage tree from a WT clonotype that had GC sharing. Inferred nodes are open circles and observed sequences are filled circles labelled with arbitrary numbers (1, 2, etc.). Inferred jumping events indicated by black arrows. Colors indicate GC origin. For jump distance per node (Fig. 4E), the distance between GCs for each jump event was summed and divided by the total number of inferred nodes in the tree. X-axis is Levenshtein distance from root. Jump events that occurred before LD = 1 (dashed line) were filtered out for analysis in (G). (G) Number of jumps normalized to the number of tree nodes for each clonotype as in Fig. 4E, but with jumps at LD < 1 filtered out (tree nodes not filtered). (H) The LD from root for each jump event from all clonotype trees, with jumps at LD < 1 filtered out. In (G-H), box plot horizontal lines indicate median and quartiles, with Tukey whiskers outside box. (I) Somatic hypermutations (SHMs) for cells 6, 7, and 5 from tree in (F). Red lines indicate SHMs compared to inferred germline, and nucleotide that the position was mutated to is indicated. Junction nucleotides (germline sequence unknown) not shown. (J) For each clonotype tree, neighboring pairs of cells that had different GC origins and shared at least one SHM (same position and nucleotide change) were tabulated, and the fraction of pairs with at least one shared SHM in a framework region (dark grey) was calculated. The fraction of pairs with at least one shared framework SHM or at least two shared CDR SHMs shown in light grey. 104 pairs analyzed for WT, 212 pairs analyzed for Gα13^ΔB^. (K) Fraction λ light chain among GC B cells that were zipcoded with photo-uncaging and sorted for single-cell ZipSeq. (L) Reciprocal of Simpson’s diversity index computed for ZipSeq-sorted cells. Lower values correspond to less diversity. Each symbol in (K, L) represents a set of zipcoded sections from Fig. 4 and Supplementary Fig. 8. Number of cells used for analysis ranged from ~500-1500 productively rearranged cells per set. In (G-H, J-L), n = 3 control, 3 Gα13^ΔB^ mice; three independent experiments. Statistical analysis was performed using two-tailed unpaired student’s t-test in (G-H, K) and two-tailed log-normal Welch’s t-test in (L). Box plot indicates median and quartiles in (G, H). Lines indicate mean and SD in (K) and geometric mean and geometric SD in (L).

**Extended Data Figure 5.**
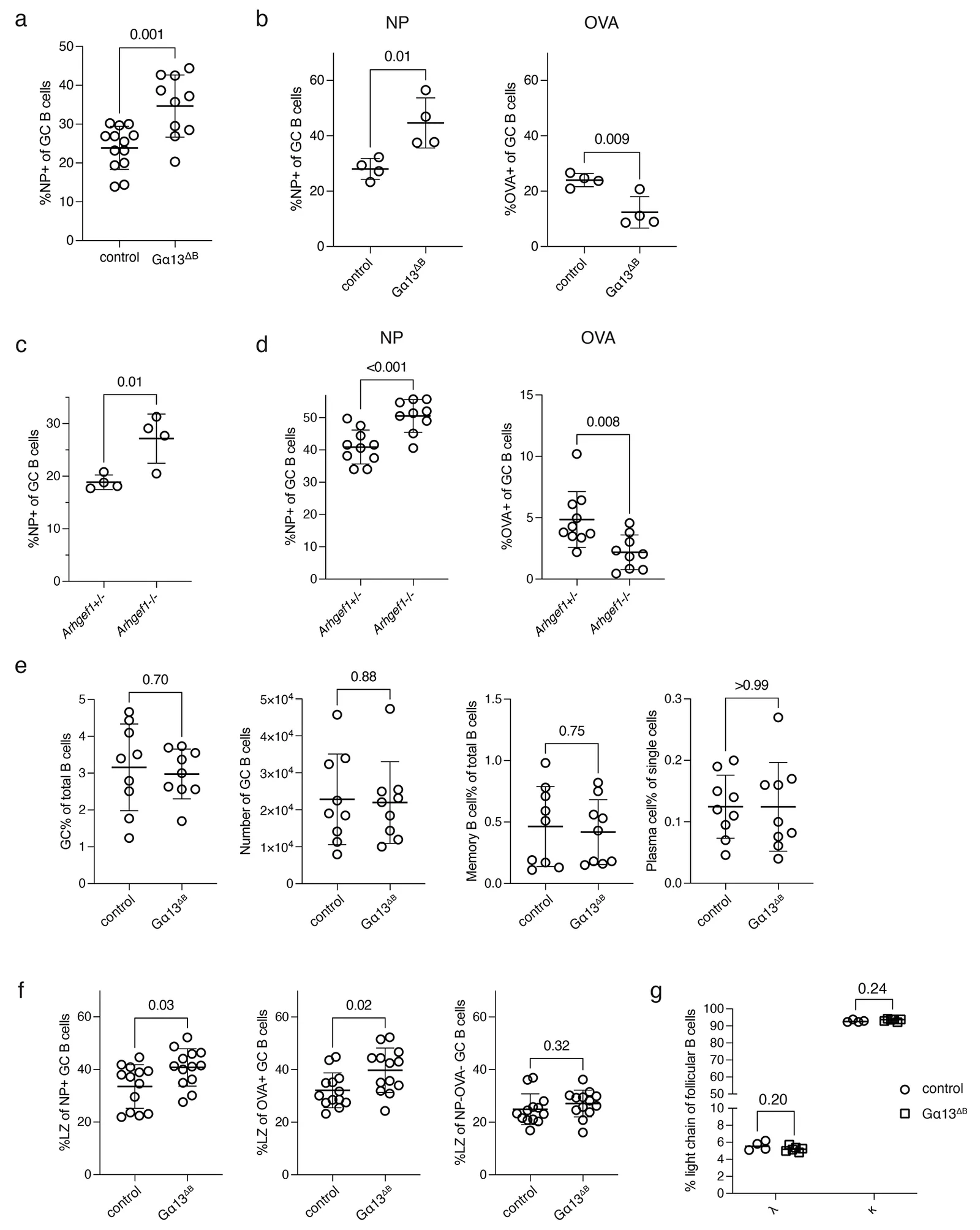
Quantification of GC magnitude, output, and polarity in Gα13^ΔB^ mice. (A) NP+ fraction of GC B cells from popliteal LNs as in Fig. 6A (day 12 NP-KLH in alum), but with *Gna13*^fl/+^ Cre^Cd23^ and *Gna13*^fl/fl^ Cre^Cd23^ mice. n = 7 control, 5 Gα13^ΔB^ mice; four independent experiments. (B) NP+ and OVA+ fraction of GC B cells from popliteal LNs as in Fig. 6C (day 12 NP9-OVA in alum), but with *Gna13*^fl/+^ Cre^Mb1^ and *Gna13*^fl/fl^ Cre^Mb1^ mice. n = 2 control, 2 Gα13^ΔB^ mice; two independent experiments. (C) NP+ fraction of GC B cells from popliteal LNs day 12 post footpad NP-KLH in alum for *Arhgef1*^+/−^ and *Arhgef1*^−/−^ mice. n = 2 *Arhgef1*^+/−^, 2 *Arhgef1*^−/−^ mice; two independent experiments. (D) NP+ and OVA+ fraction of GC B cells from popliteal LNs day 10 post footpad NP9-OVA in alum for *Arhgef1*^+/−^ and *Arhgef1*^−/−^ mice. n = 5 *Arhgef1*^+/−^, 5 *Arhgef1*^−/−^ mice; three independent experiments. (E) GC percent of total B cells, absolute number of GC B cells, memory B cell CD19+ CD38+ IgD− GL7- percent of total B cells, and plasma cell B220low CD138high percent of single cells for control (Cre^Mb1^ or Cre^Cd23^) and Gα13^ΔB^ mice (*Gna13*^fl/fl^ Cre^Mb1^ or *Gna13*^fl/fl^ Cre^Cd23^) at day 12 post NP9-OVA in alum immunization. n = 5 control, 5 Gα13^ΔB^ mice; four independent experiments. (F) Percent LZ (CD86hi, CXCR4low) of NP+, OVA+, and NP− OVA− double negative GC B cells from control (Cre^Mb1^ or Cre^Cd23^) and Gα13^ΔB^ mice (*Gna13*^fl/fl^ Cre^Mb1^ or *Gna13*^fl/fl^ Cre^Cd23^) at day 12 post NP9-OVA in alum immunization. n = 7 control, 7 Gα13^ΔB^ mice; four independent experiments. (G) Percent of naïve splenic follicular B cells that are κ or λ light chain from *Gna13*^fl/+^ Cre^Mb1^ or *Gna13*^fl/fl^ Cre^Mb1^ mice. n = 2 control, 3 Gα13^ΔB^ mice; two independent experiments. Each datapoint represents a LN in (A-F) or spleen (G). Statistical analysis was performed using two-tailed unpaired student’s t-test (A-G). All graphs display mean ± s.d.

**Extended Data Figure 6.**
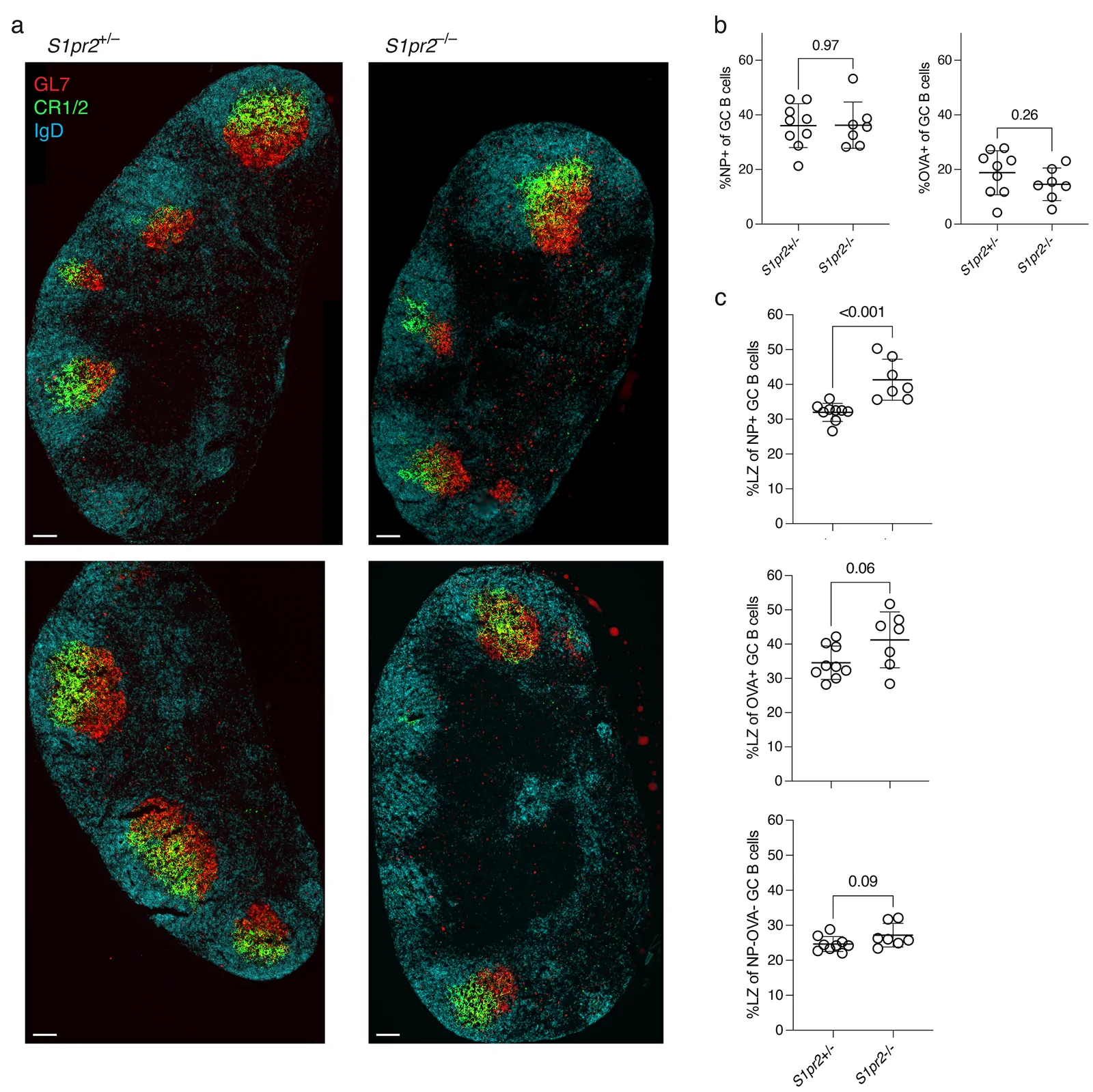
S1pr2 deficiency is not sufficient to disperse popliteal LN GCs or favor NP immunodominance. (A) IF sections of popliteal LNs day 12 post NP9-OVA in alum immunization from *S1pr2*^+/−^ and *S1pr2*^−/−^ mice. Sections representative of 2 mice each. Scale bars 100 μm. (B) NP+ and OVA+ fraction of GC B cells from *S1pr2*^+/−^ and *S1pr2*^−/−^ popliteal LNs day 12 post NP9-OVA in alum immunization. n = 5 *S1pr2*^+/−^, 4 *S1pr2*^−/−^ mice; three independent experiments. (C) Percent LZ of NP+, OVA+, and NP− OVA− double negative GC B cells from *S1pr2*^+/−^ and *S1pr2*^−/−^ LNs. n = 5 *S1pr2*^+/−^, 4 *S1pr2*^-/−^ mice; three independent experiments. Each datapoint represents a LN in (B-C). Statistical analysis was performed using two-tailed unpaired student’s t-test in (B-C). All graphs display mean ± s.d.

**Extended Data Figure 7.**
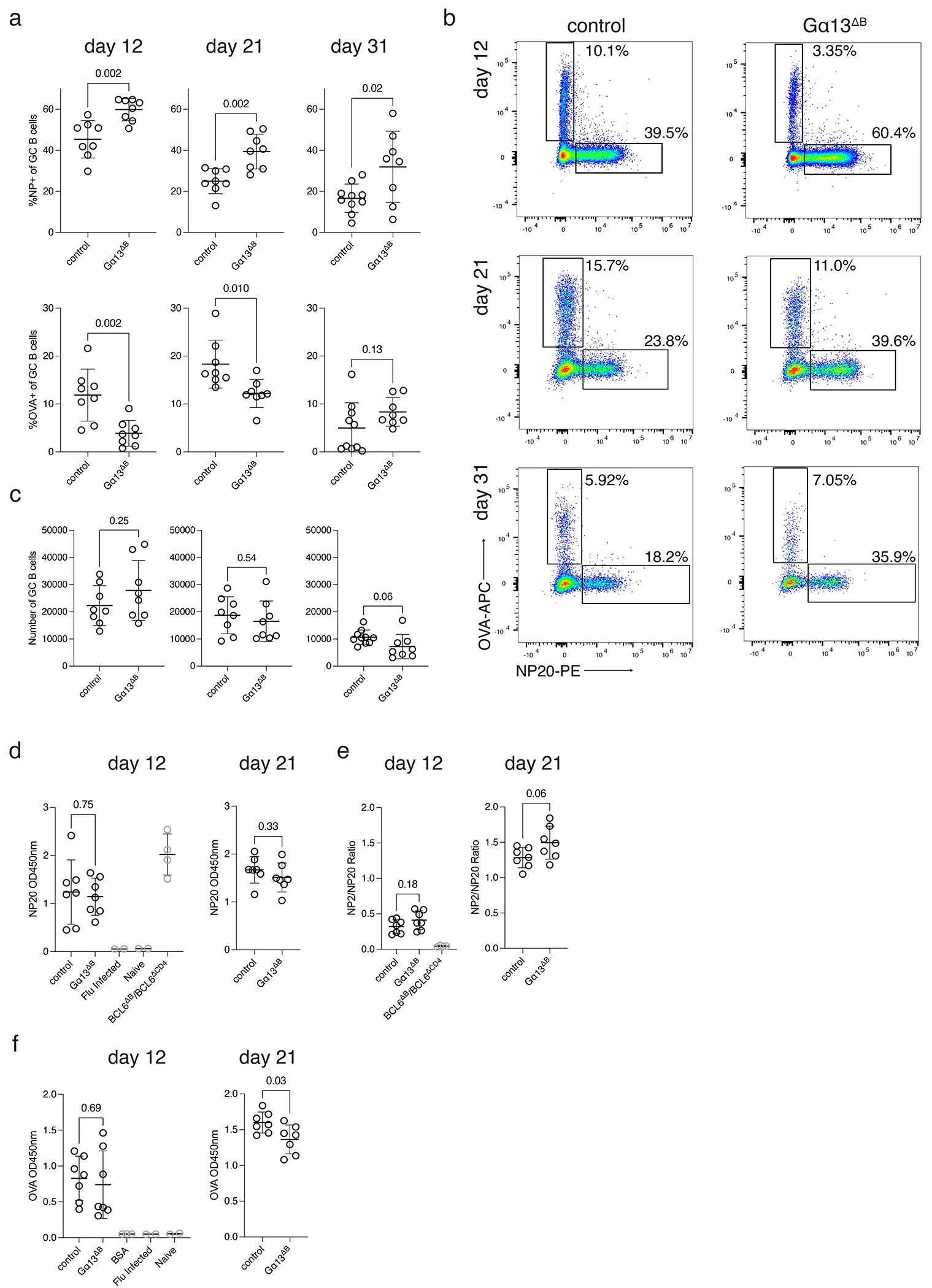
Increased NP dominance over OVA in LNs with dispersed GCs is maintained over time. (A) Percent NP+ (top row) and OVA+ (bottom row) of GC B cells at day 12, 21 and 31 post NP9-OVA in alum immunization for control (Cre^Mb1^ or Cre^Cd23^) and Gα13^ΔB^ mice (*Gna13*^fl/fl^ Cre^Mb1^ or *Gna13*^fl/fl^ Cre^Cd23^). (B) Example flow cytometry plots of NP20-PE and OVA-tetramer staining in GC at day 12, 21, and 31 post-immunization. (C) Quantification of number of GC B cells over time at day 12, 21, and 31. Each datapoint represents a LN in (A, C). In (A, C), n = 4-5 control mice per timepoint, 4 Gα13^ΔB^ mice per timepoint; three independent experiments. (D) Plasma ELISA analysis of total anti-NP antibody (1:4500 dilution) using NP20-BSA coated plates at day 12 and 21. Plasma from PR/34 influenza infected mice and naïve mice shown as negative control. Day 12 NP9-OVA immunized *Bcl6*^l/fl^ Cre^Cd4^ or *Bcl6*^l/fl^ Cre^Cd23^ mice used as control in (E). (E) Ratio of antibody titer from NP2-BSA (1:1500 dilution) and NP20-BSA coated plates at day 12 and 21. Day 12 NP9-OVA immunized *Bcl6*^l/fl^ Cre^Cd4^ or *Bcl6*^l/fl^ Cre^Cd23^ mice used as negative control. In (D-E), n = 7 control mice, 7 Gα13^ΔB^ mice, 2 flu infected WT mice, 2 naïve WT mice, 4 BCL6^ΔB^/BCL6^ΔCD4^ mice; three independent experiments. (F) Plasma ELISA analysis of OVA binding antibody (1:500 dilution) at day and 21, with day 12 NP9-OVA plasma incubated in BSA coated wells as negative control. Plasma from PR/34 infected and naïve mice on OVA coated wells also shown. Each datapoint represents a mouse plasma in (D-F). n = 7 control mice, 7 Gα13^ΔB^ mice, 3 WT mice on BSA coated wells, 2 flu infected WT mice, 2 naïve WT mice; three independent experiments. Statistical analysis was performed using two-tailed unpaired student’s t-test. All graphs display mean ± s.d.

**Extended Data Figure 8.**
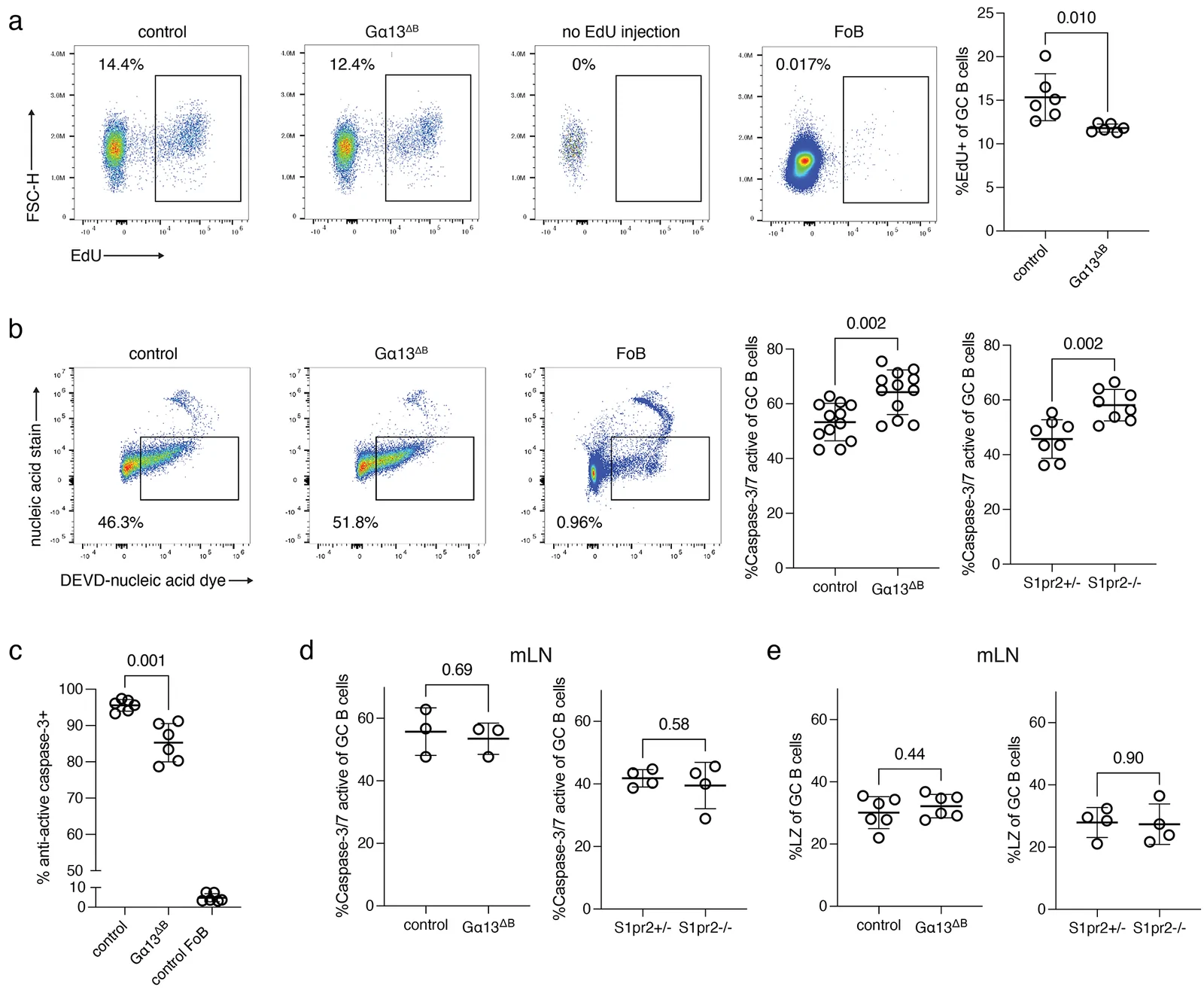
GC B cells from Gα13^ΔB^ popliteal LNs do not over-proliferate or show increased resistance to apoptosis. (A) Example flow cytometry gating of in-vivo EdU incorporation in control (Cre^Mb1^→WT chimeras) and Gα13^ΔB^ (*Gna13*^fl/fl^ Cre^Mb1^→WT chimeras) GC B cells 2 hrs post-injection, from popliteal LNs day 13 post NP-KLH in alum. CD19+ IgD+ follicular B cells and GC B cells from a mouse with only vehicle injection shown as negative controls. Quantification to the right. n = 3 control mice, 3 Gα13^ΔB^ mice; two independent experiments. (B) Example flow cytometry gating of caspase-3/7 enzymatic activity signal from control and Gα13^ΔB^ GC B cells from popliteal LNs day 12 post NP-KLH in alum after 15 min of 37°C incubation with DEVD peptide bait conjugated to nucleic acid dye. CD19+ IgD+ follicular B cells (FoB) with the same incubation shown as control. Quantification of Gα13^ΔB^ and *S1pr2*^™/™^ mice shown to the right. n = 6 control mice, 6 Gα13^ΔB^ mice; two independent experiments; 4 *S1pr2*^+/™^ mice, 4 *S1pr2*^™/™^ mice; two independent experiments. (C) Direct antibody staining of the active form of caspase-3 in GC or follicular B cells incubated at 37°C for 3 hrs from popliteal LNs 12 days post NP-KLH in alum. n = 3 control mice, 3 Gα13^ΔB^ mice; two independent experiments. (D) Caspase-3/7 enzymatic activity, similar to (B), in GC B cells from mLN of Gα13^ΔB^ and *S1pr2*^™/™^ mice. n = 3 control mice, 3 Gα13^ΔB^ mice; two independent experiments; 4 *S1pr2*^+/™^ mice, 4 *S1pr2*^™/™^ mice; two independent experiments. (E) GC B cell LZ percentages from mLN of Gα13^ΔB^ and *S1pr2*^™/™^ mice. n = 6 control mice, 6 Gα13^ΔB^ mice; two independent experiments; 4 *S1pr2*^+/™^ mice, 4 *S1pr2*^™/™^ mice; two independent experiments. Each datapoint represents a LN in (A-E). Statistical analysis was performed using two-tailed unpaired student’s t-test. All graphs display mean ± s.d.

**Extended Data Figure 9.**
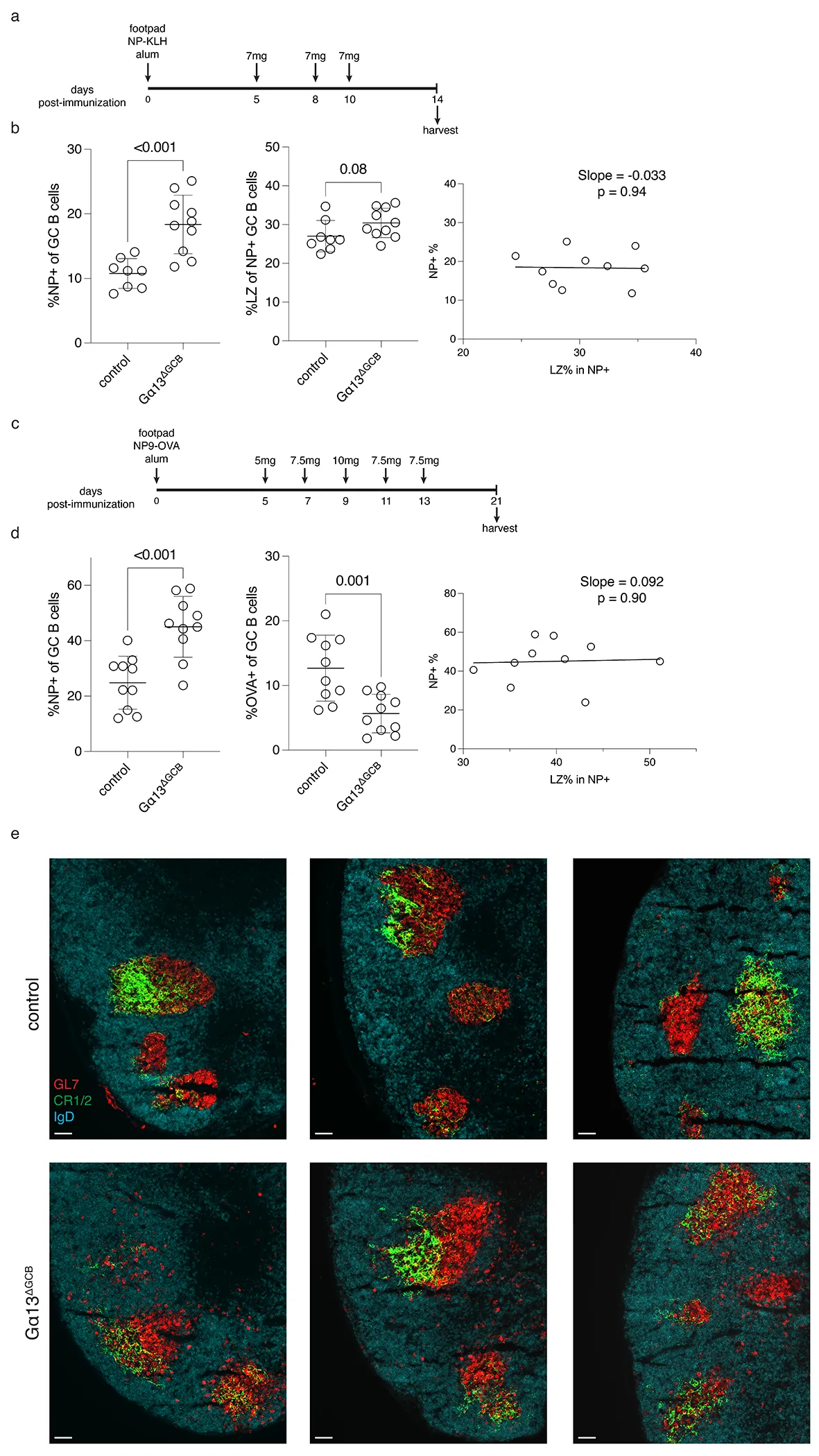
Inducible deletion of Gα13 in GC B cells reproduces the NP and OVA shifts in representation. (A) Schematic of NP-KLH immunization experiment in Gα13^ΔGCB^ mice. Schedule and amount of tamoxifen treatment indicated. (B) Percent NP+ (left) and LZ (middle) cells among popliteal LN GC B cells in control and Gα13^ΔGCB^ mice. Correlation between LZ and NP+ fractions among Gα13^ΔGCB^ LNs shown to right. n = 4 control mice, 5 Gα13^ΔGCB^ mice; three independent experiments. (C) Schematic of NP9-OVA immunization experiment in Gα13^ΔGCB^ mice. Schedule and amount of tamoxifen treatment indicated. (D) Percent NP+ (left) or OVA+ (middle) cells among popliteal LN GC B cells in control and Gα13^ΔGCB^ mice. Correlation between LZ and NP+ fractions among Gα13^ΔGCB^ LNs shown to right. n = 5 control mice, 5 Gα13^ΔGCB^ mice; three independent experiments. (E) Thin IF cryosections of neighboring GCs from popliteal LNs of control and Gα13^ΔGCB^ mice day 21 post NP-KLH immunization with tamoxifen treatment schedule as in (C). Scale bar 50 μm. Sections representative of 3 mice each. Each datapoint represents a LN in (B, D). Statistical analysis was performed using two-tailed unpaired student’s t-test or simple linear regression. Graphs display mean ± s.d.

**Extended Data Figure 10.**
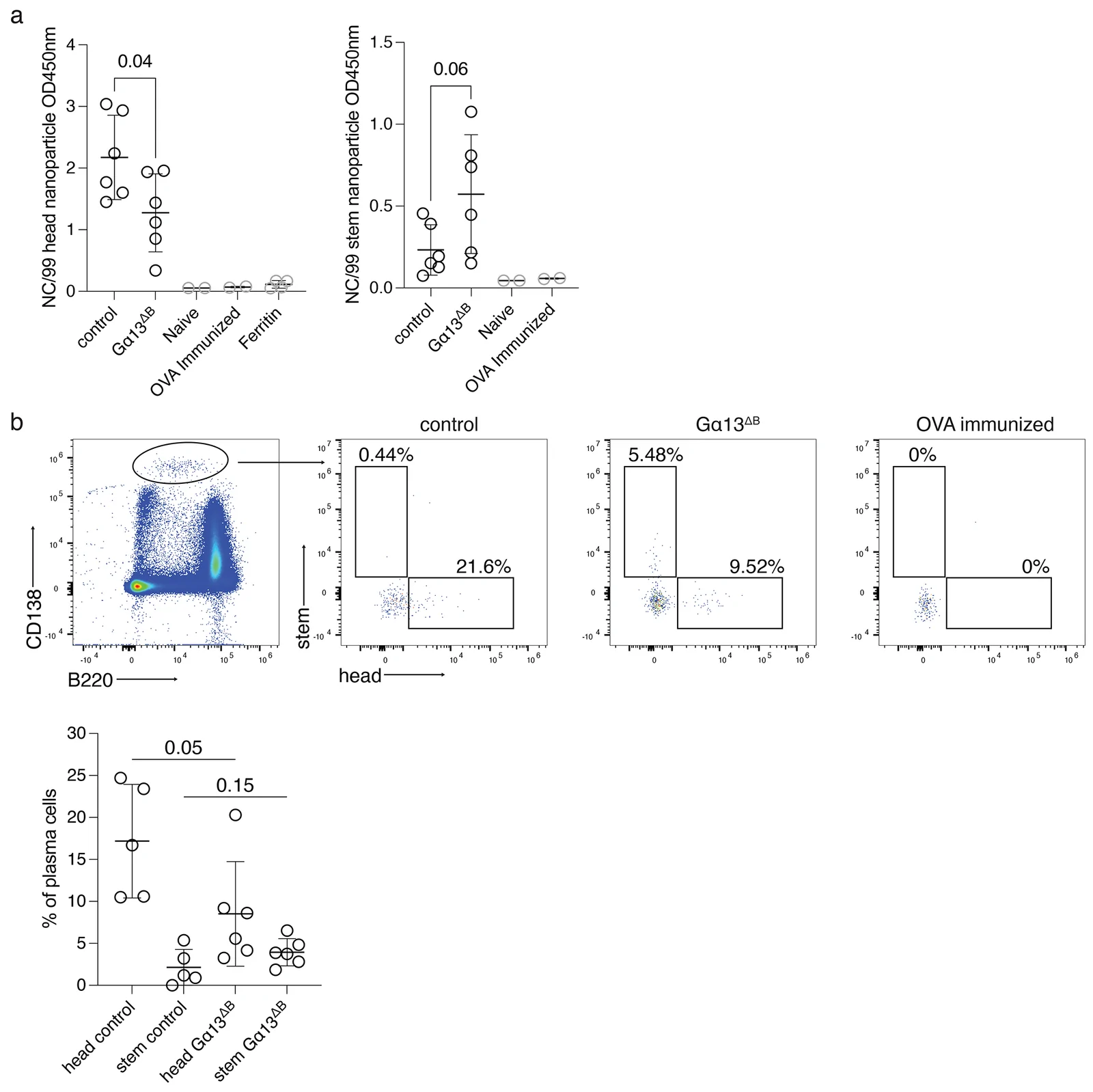
PC and circulating antibody changes in influenza HA immunized Gα13^ΔB^ mice. (A) Plasma ELISA analysis of NC/99 head (left) and stem (right) nanoparticle binding antibody (1:750 dilution for head, 1:2250 dilution for stem) at day 12 post-immunization with NC/99 HA trimer in alum. Plasma from naïve and day 12 OVA immunized mice shown as controls. Plasma from NC/99 HA immunized mice incubated on ferritin only wells also shown. Each datapoint represents a mouse plasma. n = 6 control mice, 6 Gα13^ΔB^ mice, 2 naïve WT mice, 2 OVA immunized WT mice, 4 NC/99 HA immunized WT mice on ferritin-coated wells; three independent experiments. (B) Example flow cytometry gating of head and stem nanoparticle binding on CD138high, B220low PCs from popliteal LNs day 12 post-immunization with NC/99 HA. OVA immunized popliteal LN shown as control. Quantification of PC staining shown below. Each datapoint represents a LN. n = 3 control mice, 3 Gα13^ΔB^ mice; two independent experiments. Statistical analysis was performed using two-tailed unpaired student’s t-test. All graphs display mean ± s.d.

## Supplementary Material

Supplementary Information and Supplementary Figures

Supplemental Movie Legends

Movie S1

Movie S2

Movie S3

Movie S4

Movie S5

Movie S6

Movie S7

Movie S8

Movie S9

Movie S10

Movie S11

Movie S12

Movie S13

Movie S14

Movie S15

Movie S16

Movie S17

## Figures and Tables

**Figure 1.**
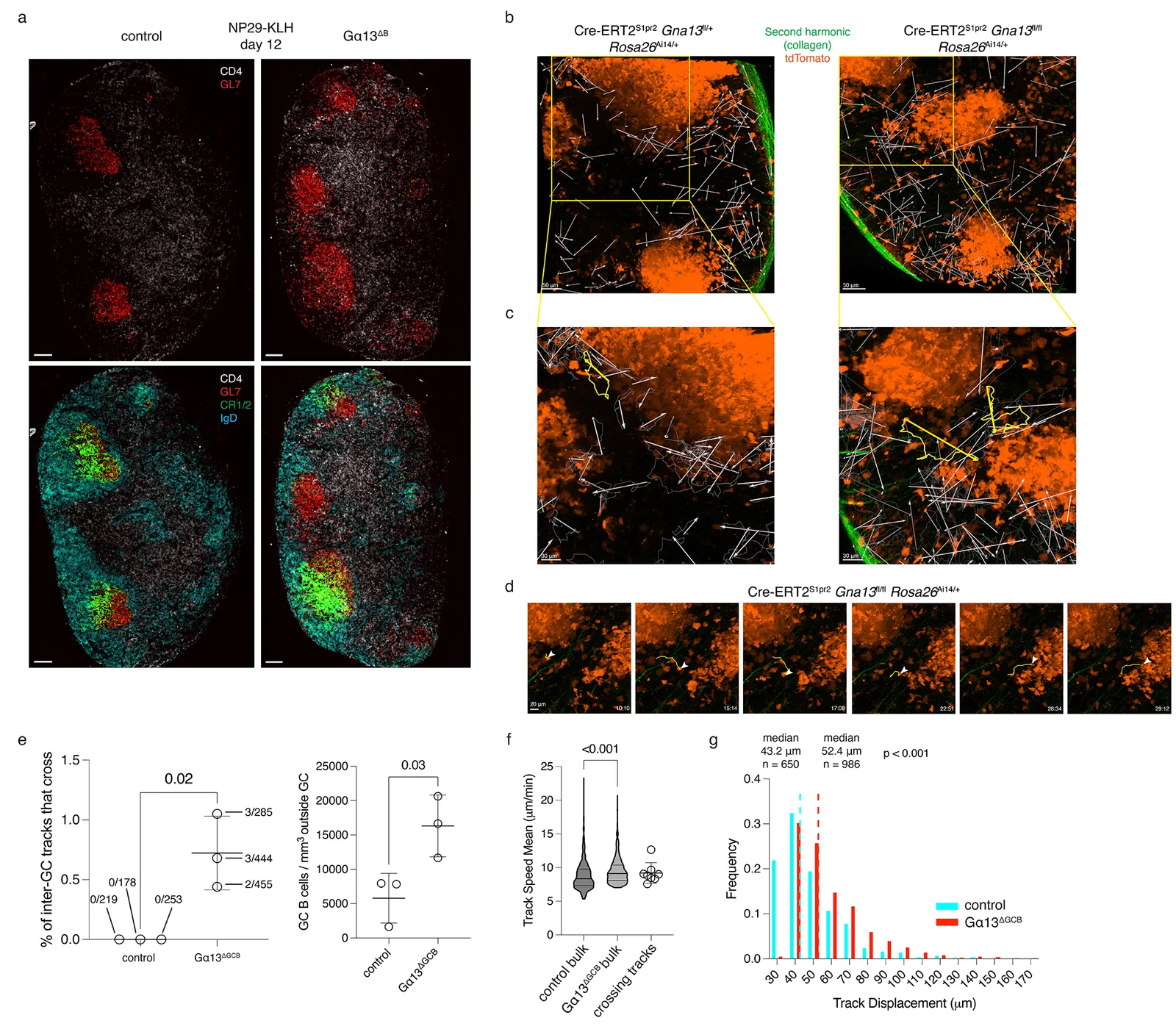
When lacking a confinement cue, GC B cells can migrate between GCs. (A) IF sections of control (*Gna13*^fl/+^ Cre^Cd23^, left) or Gα13^ΔB^ (*Gna13*^fl/fl^ Cre^Cd23^, right) popliteal LNs 12 days post footpad NP29-KLH in alum immunization. Sections stained for CD4, GL7, CR1/2, and IgD. Image without IgD and CR1/2 channels shown in top row. Sections representative of 3 mice. Micrographs of additional LN slices in Extended Data Figure 1. Scale bar 100 μm. (B) Overview two-photon stacks of explanted popliteal LNs at day 19 post-immunization (tamoxifen gavage on day 15 and 17) with NP-KLH in alum from control reporter (*Gna13*^fl/+^ Cre-ERT2^S1pr2^ *Rosa26*^Ai14/+^) and inducible Gα13^ΔGCB^ reporter (*Gna13*^fl/fl^ Cre-ERT2^S1pr2^ *Rosa26*^Ai14/+^) mice. Displacement vectors in gray show the start and end positions of Ai14+ GC B cells tracked outside the main GC aggregates. Additional GC B cell tracking movies from three independent experiments in Movies S1–S6. Scale bar 50 μm. (C) Insets from yellow boxes on overview stacks in (B) highlighting in yellow a track that exits a GC but turns back into the GC in the control LN (left) and two tracks that exit the upper GC and traffic down toward another GC before fading into the dense central GC mass in the Gα13^ΔGCB^ LN (right). Other tracks shown in thin gray lines. Scale bar 30 μm. (D) Time course snapshots of one of the highlighted inter-GC crossing tracks in the Gα13^ΔGCB^ LN in (B). Time in min:sec indicated on bottom right. Trailing track in yellow displays the last 350 sec. Cell body indicated with white arrowhead. Scale bar 20 μm. (E) Quantification of the number of inter-GC crossing tracks out of total tracks outside the GCs (left) and density of Ai14+ GC B cells outside main GC aggregates (right). Raw fraction values indicated for each data point for crossing tracks (left). Each symbol represents a LN. Statistical analysis was performed using two-tailed unpaired student’s t-test. Graphs display mean ± s.d. (F) Track speed means for tracks outside the GCs from control and Gα13^ΔGCB^ LNs. Track data shown as violin plots for “bulk” columns and as individual datapoints for the inter-GC crossing tracks. Violin plot lines indicate median and quartiles. Two-tailed Kolmogorov-Smirnov test used to compare distributions of track speed means between control and Gα13^ΔGCB^. Graph displays mean ± s.d. for crossing tracks. (G) Histogram of net displacement distances of tracks from (F). Histogram bin centers indicated on x-axis. Median displacement values indicated with dashed lines and number of tracks analyzed indicated on right. Two-tailed Kolmogorov-Smirnov test used to compare distributions of displacements between control and Gα13^ΔGCB^. In (E-G), n = 3 control, 3 Gα13^ΔGCB^ mice; three independent experiments.

**Figure 2.**
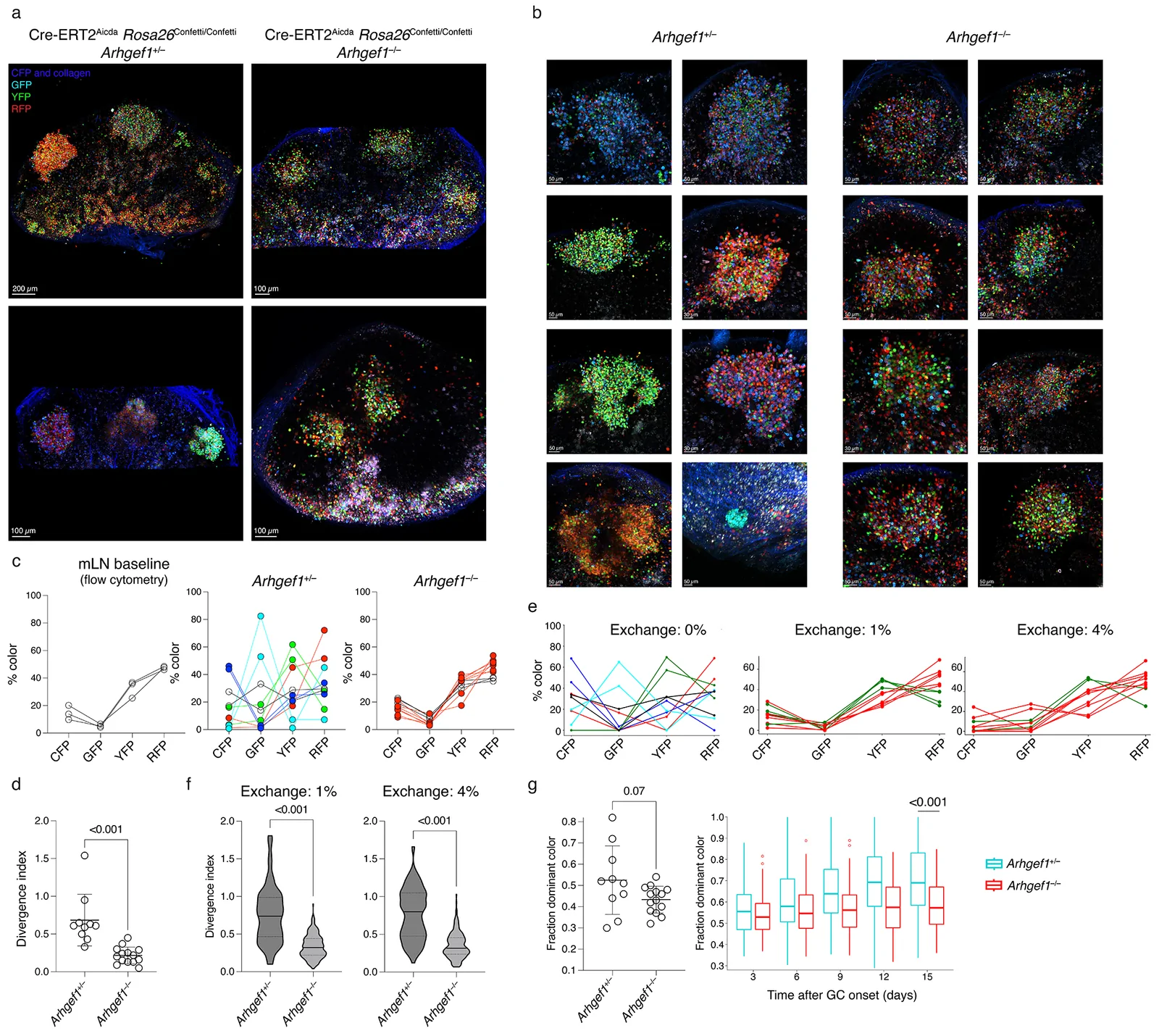
De-confined GCs become homogenized in their reporter color palette in Confetti fate-mapping mice. (A) Example overview tiled stacks from multiphoton imaging of 200 μm slices of unfixed agarose embedded popliteal LNs day 20 after CGG in alum immunization (10 mg tamoxifen gavage on day 5 and 7) from *Arhgef1*^+/−^ or *Arhgef1*^−/−^ mice crossed to the Cre-ERT2^Aicda^ *Rosa26*^Confetti/Confetti^ reporter background. Scale bars 200 μm (top left) or 100 μm. Additional images in Supplementary Fig. 3. (B) Close-up images of additional GCs from *Arhgef1*^+/−^ or *Arhgef1*^−/−^ LNs. Scale bar lengths indicated in figure. Additional images in Supplementary Fig. 3. (C) Baseline Confetti color representation (percent of Confetti reporter+ GC B cells) quantified by flow cytometry of mLNs from unimmunized *Arhgef1*^+/−^ Cre-ERT2^Aicda^ *Rosa26*^Confetti/Confetti^ mice (left). Lines connect color percents from the same mLN. Quantification of Confetti reporter colors from representative GCs from *Arhgef1*^+/−^ (middle) or *Arhgef1*^−/−^ (right) LNs. Lines connect color percents from the same GC. Circles and lines are colored by the dominant color (blue for CFP, cyan for GFP, green for YFP, and red for RFP), unless the highest represented color is <40%, in which case the circles and lines were left uncolored. (D) Divergence of observed color representation from the baseline mLN representation for the imaged GCs in (C). Statistical analysis was performed using two-tailed unpaired student’s t-test. Graph displays mean ± s.d. (E) Stochastic simulation modelling of GCs given the baseline color distribution from mLN flow cytometry data and allowed to evolve over 15 days post-GC onset. Final color distributions for 10 representative GCs shown for 0%, 1%, and 4% exchange per hr of GC B cells. Lines are colored as in (C). (F) Divergence index calculated as in (D) for simulated GCs at 15 days post-GC onset. *Arhgef1*^+/−^ GCs were simulated with 0% exchange and compared to either 1% exchange (left) or 4% exchange (right) per hr conditions for *Arhgef1*^−/−^. Statistical significance assessed using two-tailed Kolmogorov-Smirnov test. 120 and 240 GCs simulated for *Arhgef1*^+/−^ and *Arhgef1*^−/−^, respectively, for both exchange conditions. Violin plot lines indicate median and quartiles. (G) Fraction of the most represented color in imaging-quantified GCs (left) from (D). Statistical analysis was performed using two-tailed unpaired student’s t-test. For comparison, fraction of the most represented color for simulated GCs shown over time for *Arhgef1*^+/−^ and *Arhgef1*^−/−^ GCs using the 1% exchange per hr condition (right). Statistical significance at day 15 assessed using two-tailed Kolmogorov-Smirnov test. 80 GCs simulated per timepoint. Graph displays mean ± s.d. For boxplots, horizontal lines indicate median and quartiles, with Tukey whiskers and outliers outside box. In (D, G), n = 4 *Arhgef1*^+/−^, 4 *Arhgef1*^−/−^ mice; four independent experiments.

**Figure 3.**
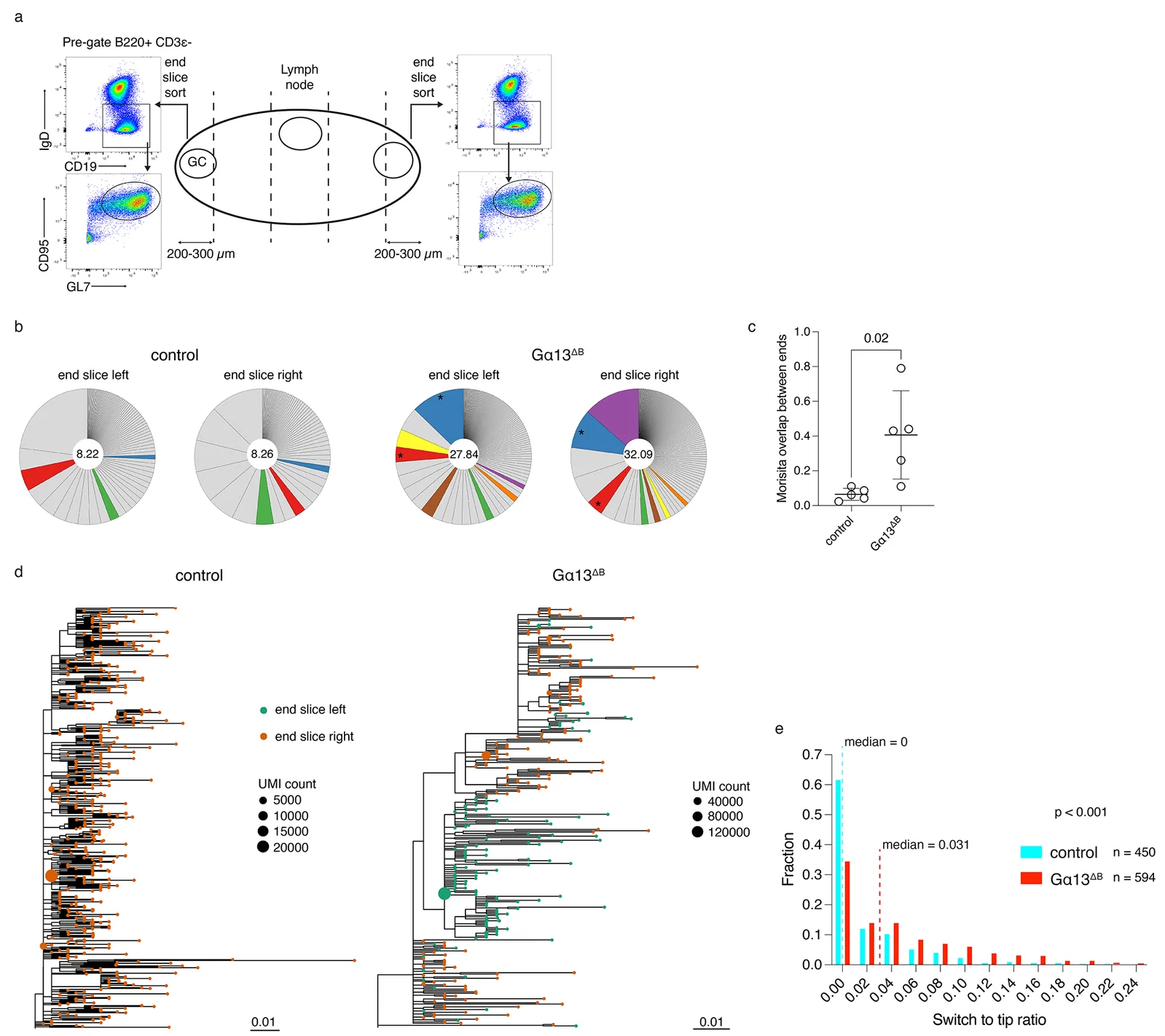
GC-dispersed LNs display elevated clonal overlap between opposite poles. (A) Example sort gating of GC B cells from opposite ends of a popliteal LN at day 12 post-immunization with NP-KLH in alum. Agarose embedded LNs were vibratome sectioned at 300 μm intervals, with the first and last slices processed for heavy chain sequencing. (B) Pie charts depicting the overlapping clones (defined here as identical CDR3 amino acid sequence and IGHV, D and J gene usage) between end slices for an example LN from control (*Gna13*^fl/+^ Cre^Cd23^) and Gα13^ΔB^ (*Gna13*^fl/fl^ Cre^Cd23^) mice. Each slice is a unique clone, and proportions are weighted by the number of UMI barcodes. Slices representing identical clones between endpieces are filled with the same color. Center number in pie indicates percentage of clones shared (UMI weighted). Asterisks indicate shared clones that rank among the largest 6 clones in both end slices. Additional LNs in Supplementary Fig. 5. (C) Quantification of clonal sharing in (B) using Morisita’s overlap measure of repertoire similarity for LN end slices from control or Gα13^ΔB^ mice. Each datapoint represents a pair of LN end slices. From each slice, ~10-30×10^3^ cells were sorted, sequenced to ~30-40×10^6^ read pairs per sample, resulting in 5-10×10^3^ unique CDR3 amino acid sequences with 20-60 sequences accounting for 50% of the population. Statistical analysis was performed using two-tailed unpaired student’s t-test. Graph displays mean ± s.d. n = 3 control, 3 Gα13^ΔB^ mice; three independent experiments. (D) BCR sequence lineage trees (largest clone) shown for an example LN from control and Gα13^ΔB^ mice. Clonal clustering based on full VDJ sequence similarities, CDR3 length, and V and J gene usage used to construct trees. Tree tips (observed sequences) colored by LN endpiece origin and dot size scaled by UMI count. Scale bar of branch lengths indicates number of substitutions per nucleotide. Additional trees in Supplementary Fig. 6. (E) Quantification of switch to tip ratio, the number of switches (flips between endpiece origin along tree topology) normalized to the number of unique members (tips) of tree, among all lineage trees from LNs in (C). Histogram bin centers indicated on x-axis. Median ratio indicated with dotted lines and number of clonotype families analyzed indicated on the right. Two-tailed Kolmogorov-Smirnov test used to compare distributions.

**Figure 4.**
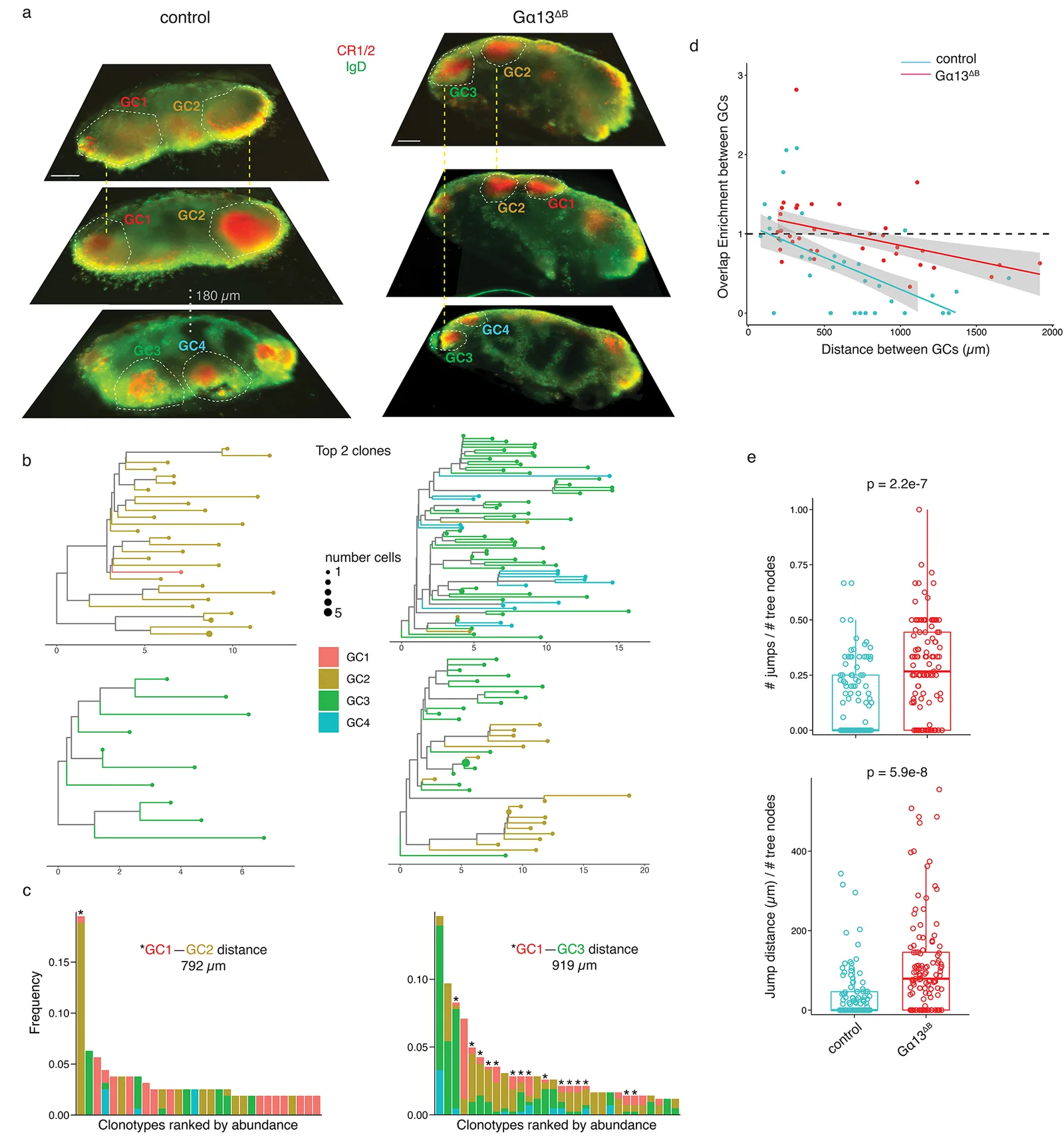
Zipcode spatial single-cell sequencing reveals increased inter-GC ‘hopping’ among clonal families in de-confined LNs. (A) Example vibratome slices of unfixed, live popliteal LNs from control (*Gna13*^fl/fl^, left) or Gα13^ΔB^ (*Gna13*^fl/fl^ Cre^Mb1^, right) mice at day 12 post NP-KLH in alum immunization. Serial 180 μm sections were stained for CR1/2 (red) and IgD (green) before photo-uncaging based zipcoding of GC regions (white dashed borders) with 365 nm light. Yellow dotted line connects GCs registered across consecutive slices. Gray dotted line indicates an instance where the subsequent slice was not consecutive (a 180 μm or one slice gap intervening in this case). Additional sections in Fig. S8. Number of successfully clonotyped paired heavy and light chain GC B cells with confident zipcode calls from these example sets of sections are 542 cells and 901 cells for control and Gα13^ΔB^, respectively. Scale bar 200 μm. (B) Phylogenetic trees of the top 2 largest clonotypes from the control (left) and Gα13^ΔB^ (right) zipcoded sections, with each member and its upstream node color coded by originating GCs from (A). Branching points represent inferred somatic hypermutation events, and x-axis scale of branch lengths is Levenshtein edit distance. Tip size indicates number of cells. (C) Histograms of the largest 30 clonotype families, with stacked bars indicating the fraction of total cells originating from each zipcoded GC in (A). One GC pair each is noted for the control (left) and Gα13^ΔB^ (right) sections to compare two pairs that are similar distance apart. Clonotype families containing cells from both GCs in the pair are highlighted with an asterisk above the stacked bars. (D) Pooled data of the overlap enrichment between all possible pairs of GCs across 3 independent experiments plotted against the shortest distance separating each pair. GCs were paired only from consecutive sections within the same LN. Each dot represents a GC pair. Best-fit linear regression shown with solid line and 95% confidence interval bands shaded in grey. (E) Pooled data of number of jumps for each clonotype family normalized to number of tree nodes for all clonotypes (upper). Summed distance of inter-GC ‘jumps’ within a clonotype family normalized to number of tree nodes (lower). Each datapoint represents a clonotype family. Box plot horizontal lines indicate median and quartiles, with Tukey whiskers outside box. Statistical analysis was performed using two-tailed unpaired student’s t-test. In (D-E), n = 3 control, 3 Gα13^ΔB^ mice; three independent experiments.

**Figure 5.**
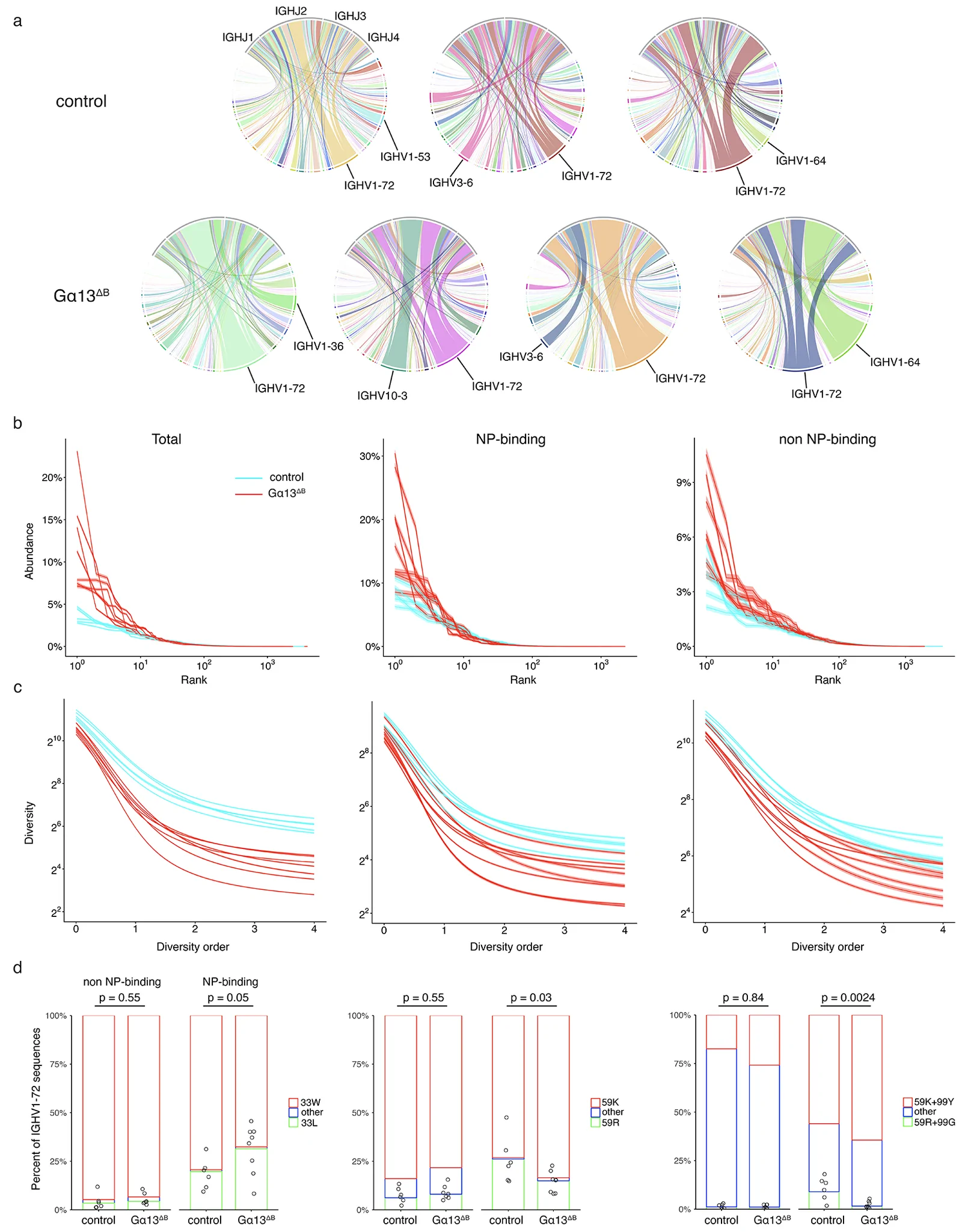
LNs with de-confined GCs are overall less diverse and more skewed towards dominant clones. (A) Chord diagrams showing the V-J gene segment pairs in the total bulk heavy chain GC repertoire at day 12 post NP-KLH in alum footpad immunization from control (*Gna13*^fl/+^ Cre^Mb1^, top) or Gα13^ΔB^ (*Gna13*^fl/fl^ Cre^Mb1^, bottom) popliteal LNs. IGHJ1–4 are shown in the top gray segments, and unique IGHV genes are shown in different colors in the lower segments. The top two represented IGHV genes from each LN are labeled. Chord widths represent the pairing frequency. Each plot represents an individual LN. (B) Rank abundance curves of repertoires from individual LNs displaying frequencies (# of unique UMIs per clonotype family / # total unique UMIs) of size-ordered clonotype families from total (left), NP+ sorted (middle), or NP- sorted (right) GC B cells. Shaded error bands represent the 95% confidence interval about the mean from 100 bootstrapping iterations, where each LN was uniformly resampled to the same number of sequences. For NP- LN samples, 55-90×10^3^ cells were sequenced per sample with 1.5-2×10^6^ read pairs per sample, resulting in 10-12×10^3^ unique sequences and 2-3×10^3^ clones after grouping, with 30-60 clones representing 50% of the population. For NP+ LN samples, 30-70×10^3^ cells were sequenced with 3-4×10^6^ read pairs per sample, resulting in 6-10×10^3^ unique sequences and 1-1.5×10^3^ clones after grouping, with 3-15 clones representing 50% of the population. (C) Hill diversity curves for LNs in (B) displaying diversity scores for diversity orders ranging from 0 to 4. Repertoires were uniformly resampled as in (B), and 95% confidence intervals about the mean from 100 bootstrapping iterations are plotted. (D) Fraction of unique V_H_1-72 sequences, weighted by UMI counts per sequence, with W, L, or another amino acid (other) at the 33^rd^ (IMGT numbering) position (left). Similar graphs shown for V_H_1-72 sequences with K59R substitution (middle) and K59R substitution with Y99G V-D junctional variant (right). Overlayed datapoints represent the W33L, K59R, or K59R+Y99G fractions for individual LNs. ‘Other’ category for K59R+Y99G righthand graph refers to sequences with a different amino acid at either position. Statistical analysis was performed using two-tailed unpaired student’s t-test. In (B-D), n = 4 control, 5 Gα13^ΔB^ mice; three independent experiments.

**Figure 6.**
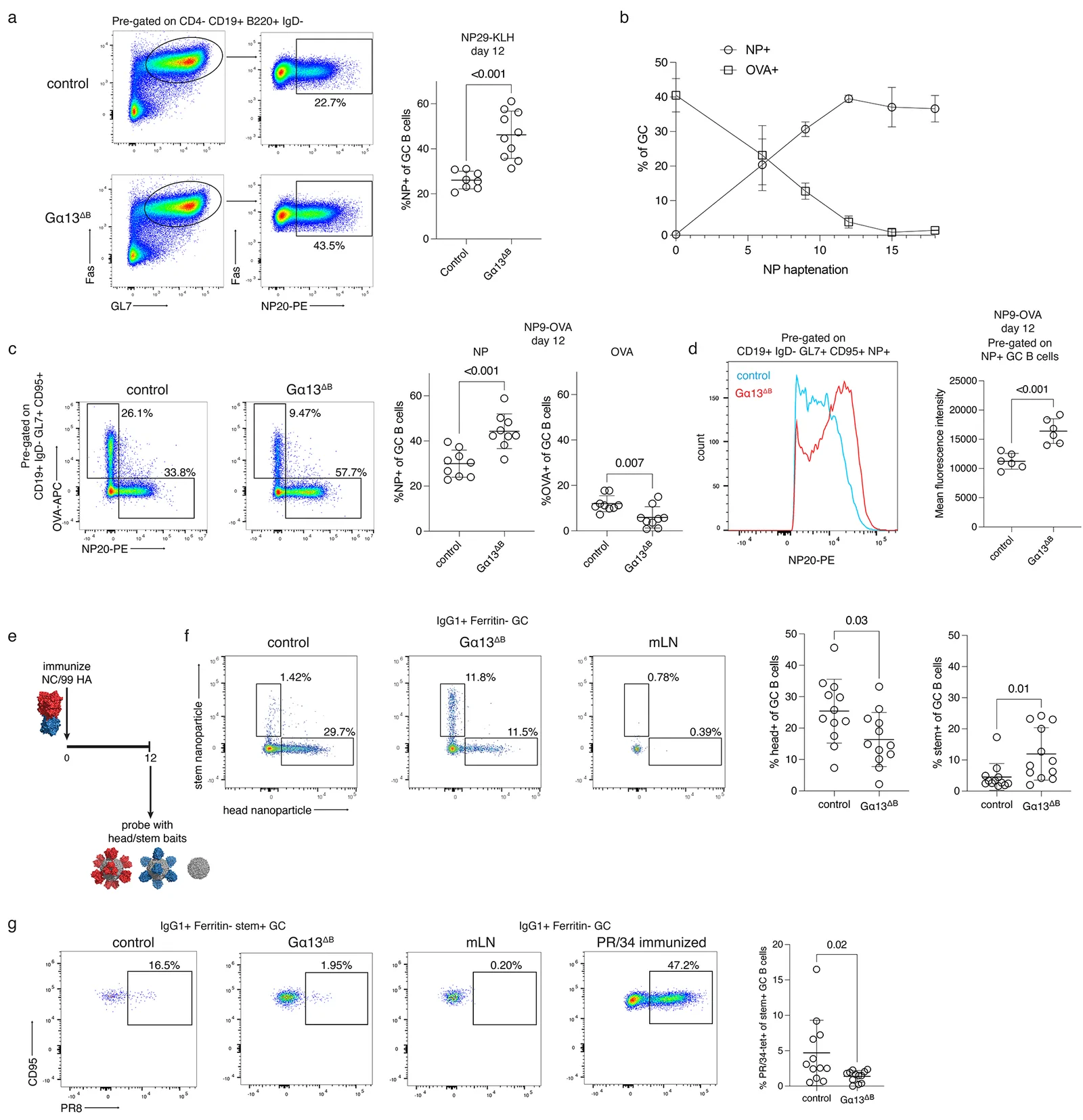
LNs with dispersed GCs suppress sub-dominant GC B cells while shifting the immunodominance hierarchy for complex antigens. (A) Example flow cytometry plots of GC population and NP+ GC fractions from control (*Gna13*^fl/+^ Cre^Mb1^) or Gα13^ΔB^ (*Gna13*^fl/fl^ Cre^Mb1^) popliteal LNs at day 12 post footpad NP-KLH in alum immunization. Quantification shown to the right. n = 4 control, 5 Gα13^ΔB^ mice; three independent experiments. (B) Titration of NP haptenation on OVA protein carrier showing the fraction of NP+ (circles) or OVA+ (squares) GC B cells for increasing NP conjugation levels (day 14 post footpad immunization in alum). Each data point is the mean and standard deviation of 4 mice; two independent experiments. (C) Example flow cytometry plots of NP+ and OVA+ GC fractions from control (*Gna13*^fl/+^ Cre^Cd23^) or Gα13^ΔB^ (*Gna13*^fl/fl^ Cre^Cd23^) popliteal LNs at day 12 post footpad NP9-OVA in alum immunization. Quantification shown to the right. n = 5 control, 5 Gα13^ΔB^ mice; three independent experiments. (D) Example histogram of NP-PE staining intensity on NP+ GC B cells from day 12 NP9-OVA in alum popliteal LNs from control (*Gna13*^fl/+^ Cre^Cd23^) or Gα13^ΔB^ (*Gna13*^fl/fl^ Cre^Cd23^) mice. Mean fluorescence intensity quantification shown to the right. n = 3 control, 3 Gα13^ΔB^ mice; two independent experiments. (E) Schematic of NC/99 HA trimer in alum immunization experiment and ferritin nanoparticle bait probes. Head and stem domains of NC/99 HA protein in red and blue, respectively, and ferritin scaffold in gray. (F) Example flow cytometry plots of head (AF488) and stem (AF647) nanoparticle binding GC B cells for control and Gα13^ΔB^ mice, with mLN negative control. Pre-gated on IgG1+ GC B cells negative for ferritin-AF594 background probe. Quantification shown to the right. n = 6 control, 6 Gα13^ΔB^ mice; three independent experiments. (G) Example flow cytometry plots of PR/34 HA-biotin streptavidin tetramer binding to NC/99 stem+ GC B cells, with mLN and PR/34 footpad immunized popliteal LN (no stem+ pre-gate) as negative and positive controls, respectively. Quantification shown to the right. n = 6 control, 6 Gα13^ΔB^ mice; three independent experiments. Each symbol represents a LN in (A, C-D, F-G). Statistical analyses were performed using two-tailed unpaired student’s t-tests. All graphs display mean ± s.d.

## Data Availability

RNA-seq and spatial scRNA-seq data generated in this study have been deposited in NCBI’s Gene Expression Omnibus under accession number GSE314976, GSE314849, GSE314607, and GSE314606. Additional spatial scRNA-seq metadata and processed data are available at https://doi.org/10.5281/zenodo.22179887 ^87^. All other data are available in the main text or supplementary materials.

## Code availability

The custom GC simulation code and modelling results data are available at doi.org/10.5281/zenodo.21515363 ^88^. Custom code for scRNA-seq analysis are available at https://github.com/ken7hu/Spatial-confinement-promotes-germinal-center-diversity-and-tempers-immunodominance.
