# Supplementary material for: Spatial confinement promotes germinal center diversity and tempers immunodominance
Source: Nat Immunol. Author manuscript; Available in PMC 2026 Oct 30. (PMC13720956; doi:10.1038/s41590-026-02698-x)
Supplement: Supplementary file 1 [file NIHMS2214944-supplement-Supplementary_Information_and_Supplementary_Figures.pdf]

Materials for

Spatial confinement promotes germinal center diversity and tempers immunodominance

Chen *et al*.

The PDF file includes:

Supplementary Figures 1 to 9

Supplementary Tables 1, 2

Supplementary Methods

Germinal Center Simulation Model

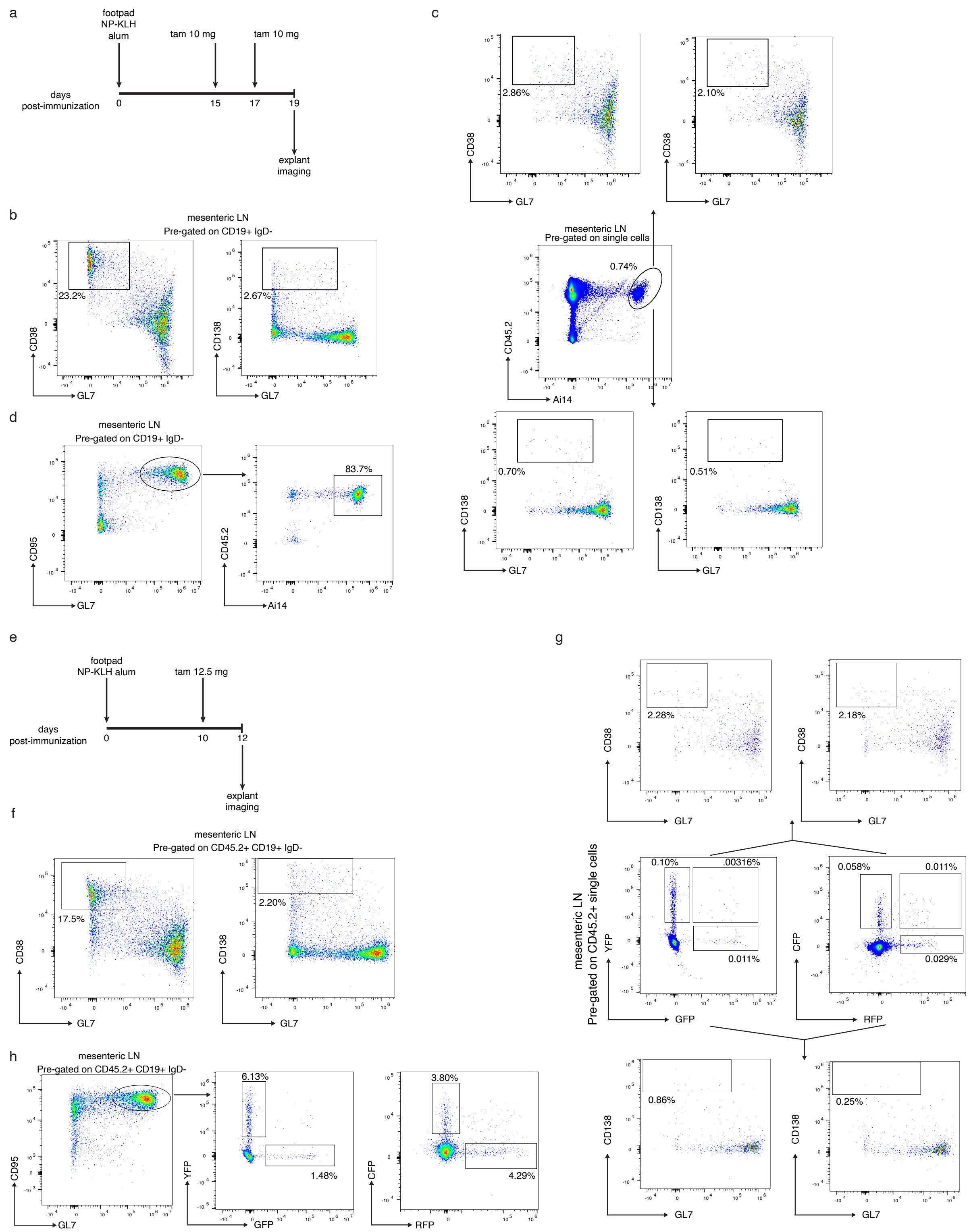

Supplementary Figure 1. Phenotype of reporter positive cells with Cre-ERT2^{S1pr2} or Cre-ERT2^{Aicda} activity.

(a) Schematic of experiment for Cre-ERT2^{S1pr2} *Rosa26*$^{Ai14/+}$ imaging (Fig. 1). Popliteal LNs were reserved for multiphoton imaging and mLNs used for flow cytometry.

(b) Example flow cytometry plot of memory B cell (left) and plasma cell (right) populations from mLN of a mouse treated as in (a). Pre-gated on CD19+ IgD- cells.

(c) Flow cytometry plots of memory B cells (top) and plasma cells (bottom) among Ai14-high cells (pre-gated on single cells) from mLNs of two representative mice treated as in (a). Memory B cell and plasma cell gates were set using the total mLN populations in (b).

(d) Representative flow cytometry plot of Ai14 reporter expression in GC B cells from mLN of a mouse treated as in (a).

(e) Schematic of experiment for *Arhgef1*$^{+/-}$ or *Arhgef1*$^{-/-}$ Cre-ERT2^{Aicda} *Rosa26*$^{Confetti/Confetti}$ imaging (Extended Data Fig. 2). Popliteal LNs were reserved for multiphoton imaging and mLNs used for flow cytometry.

(f) Representative flow cytometry plot of memory B cell (left) and plasma cell (right) populations from mLN of a mouse treated as in (e). Pre-gated on CD45.2+ CD19+ IgD- cells.

(g) Flow cytometry plots of memory B cells (top) and plasma cells (bottom) among Confetti reporter+ (YFP, GFP, CFP, or RFP) cells (pre-gated on CD45.2+ single cells) from mLNs of two representative mice treated as in (e). Memory B cell and plasma cell gates were set using the total mLN populations in (f).

(h) Representative flow cytometry plot of Confetti reporter expression in GC B cells from mLN of a mouse treated as in (e).

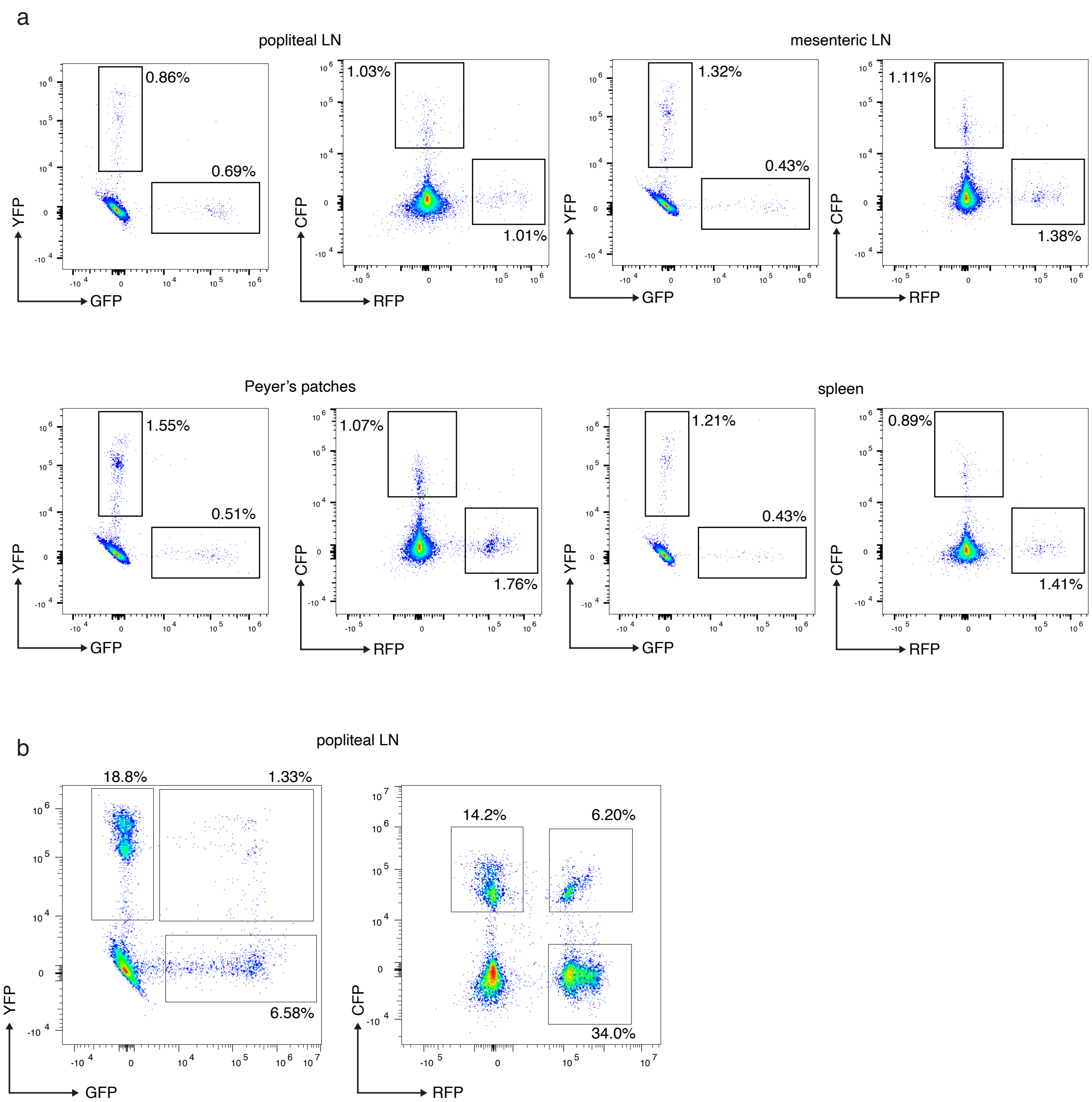

Supplementary Figure 2. Confetti reporter efficiency with Cre-ERT2^{Aicda} versus Cre-ERT2^{S1pr2}.

(a) Example flow cytometry plots of Confetti reporter expression in popliteal LN, mLN, Peyer's patches, and spleen at day 12 after NP-KLH in alum footpad and intraperitoneal immunization (10 mg tamoxifen gavage on day 5 and 7) from an *Arhgef1*$^{+/+}$ Cre-ERT2^{S1pr2} *Rosa26*$^{Confetti/Confetti}$ mouse. Pre-gated on CD19+ IgD- GL7+ CD95+ cells.

(b) Example flow cytometry plot of Confetti reporter expression in a popliteal LN at day 20 after CGG in alum immunization (10 mg tamoxifen gavage on day 5 and 7) from an *Arhgef1*$^{+/-}$ Cre-ERT2^{Aicda} *Rosa26*$^{Confetti/Confetti}$ mouse. Pre-gated as in (a).

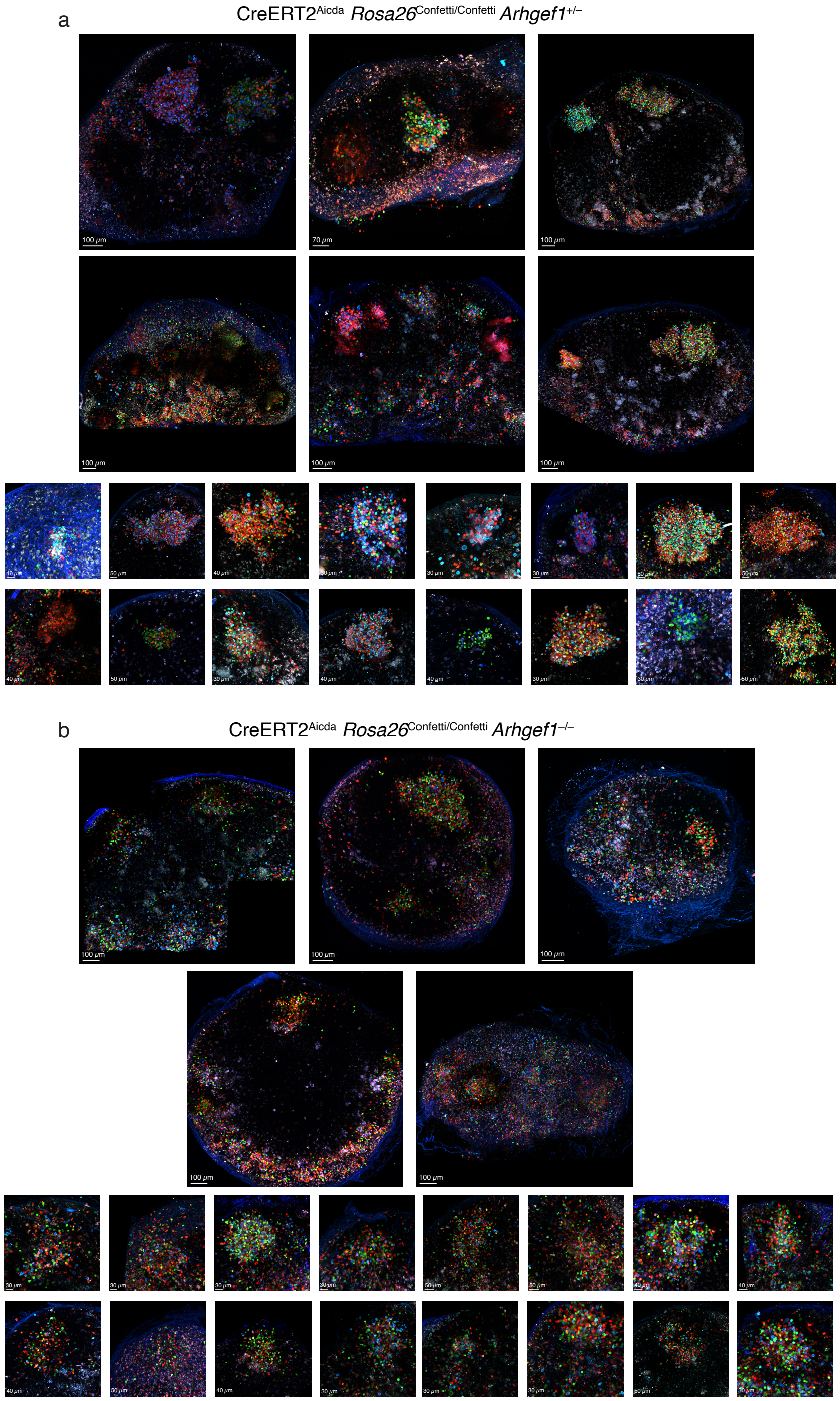

Supplementary Figure 3. Additional thick sections from Confetti reporter mice with normal or de-confined GCs.

(a) Overview tiled stacks as in Fig. 2a, b from *Arhgef1*$^{+/–}$ Confetti reporter mice, with close-up images of additional GCs below. Data pooled from 7 LNs. Scale bar lengths indicated in figure.

(b) Overview tiled stacks and close-up images of GCs from *Arhgef1*$^{–/–}$ Confetti reporter mice. Data pooled from 6 LNs. Scale bar lengths indicated in figure.

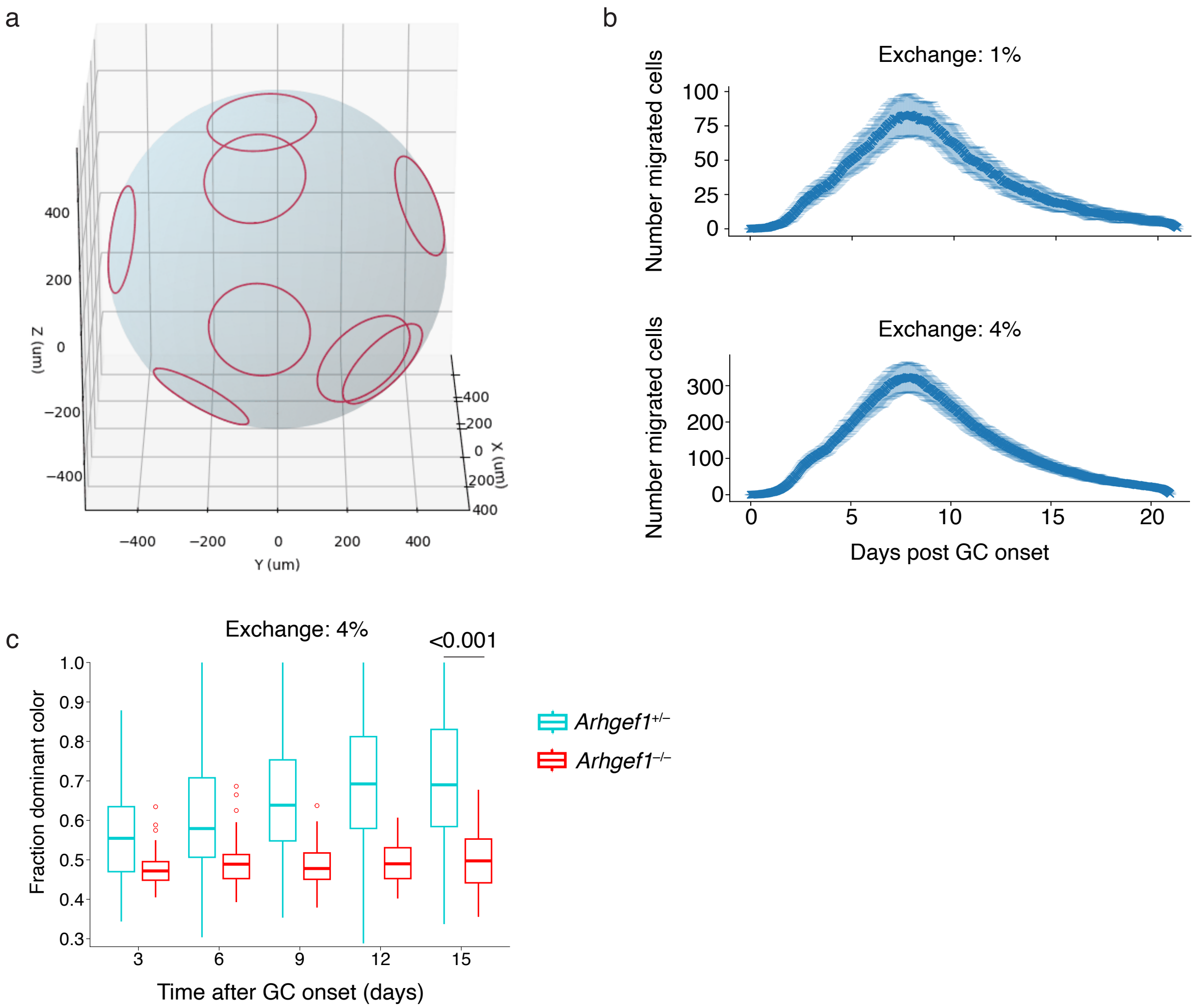

Supplementary Figure 4. Computational modelling of GC exchange in Confetti reporter mice.

(a) Schematic of modelled GCs (radius 160 μm) on a LN sphere (radius 500 μm). GC centers were randomly positioned on the sphere, with a minimum distance of 150 μm between GC borders. B cells migrate at 10 μm/min to a target GC, which is selected randomly.

(b) Mean and standard deviation of number of migrated cells out of GCs over time, for 1% and 4% exchange per hr. GC onset, which is several days post-immunization, is at day 0 in the model. In the model, GCs contract around day 20 post-onset.

(c) Fraction of the most represented color for simulated GCs shown over time for GCs with 4% exchange per hr (*Arhgef1*$^{-/-}$) compared to no exchange (*Arhgef1*$^{+/-}$), displayed as in Fig. 2g. The same *Arhgef1*$^{+/-}$ simulated GCs from Fig. 2g are displayed here for visual clarity. Statistical significance at day 15 assessed using Kolmogorov-Smirnov test. 80 GCs simulated per timepoint.

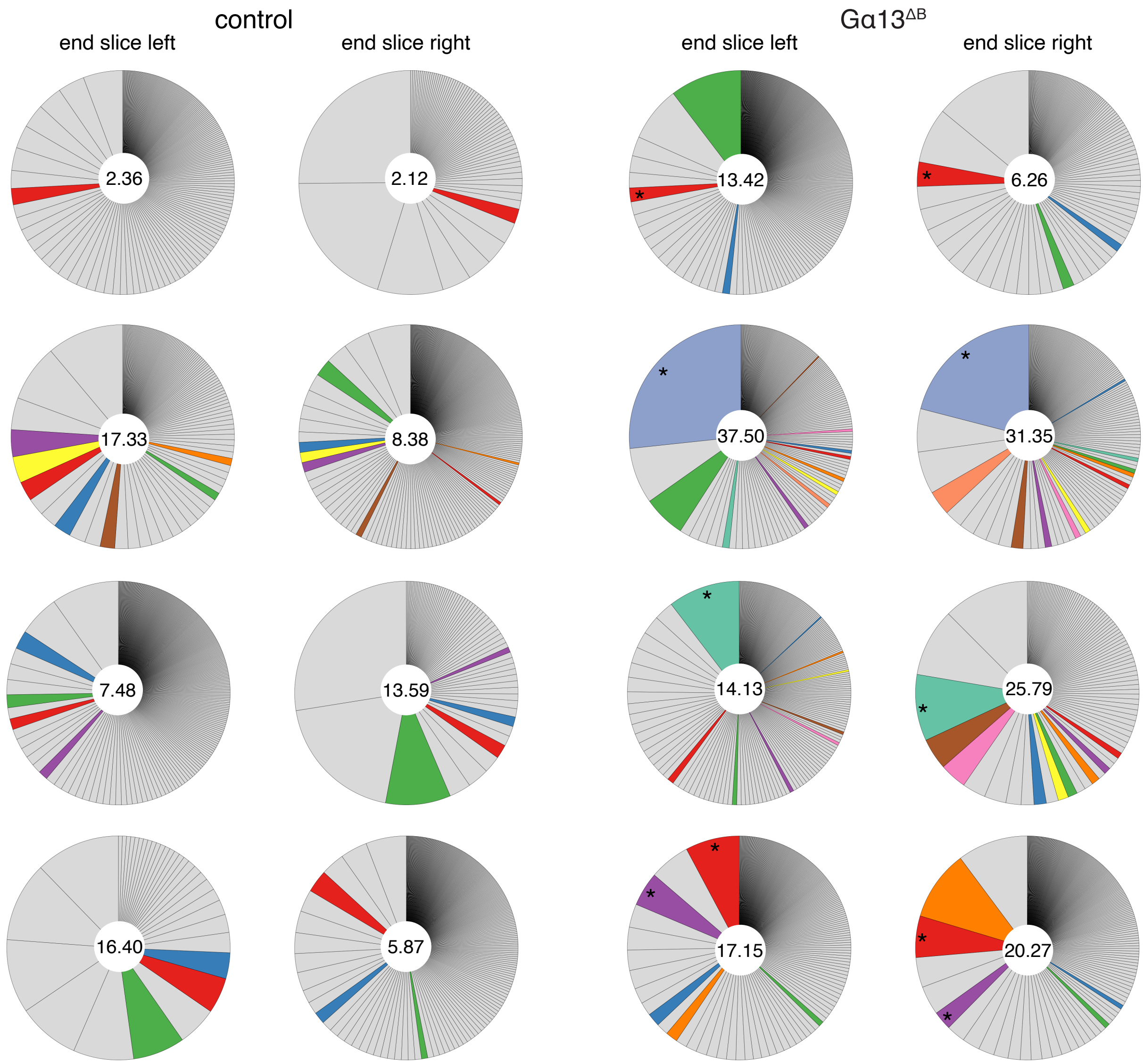

Supplementary Figure 5. Additional clonal pie charts from WT and Gα13$^{\Delta B}$ LN endpieces.

Additional pie charts displayed as in Fig. 3b. Each row is a separate LN. Paired endpieces from control (left) and Gα13$^{\Delta B}$ (right) mice shown.

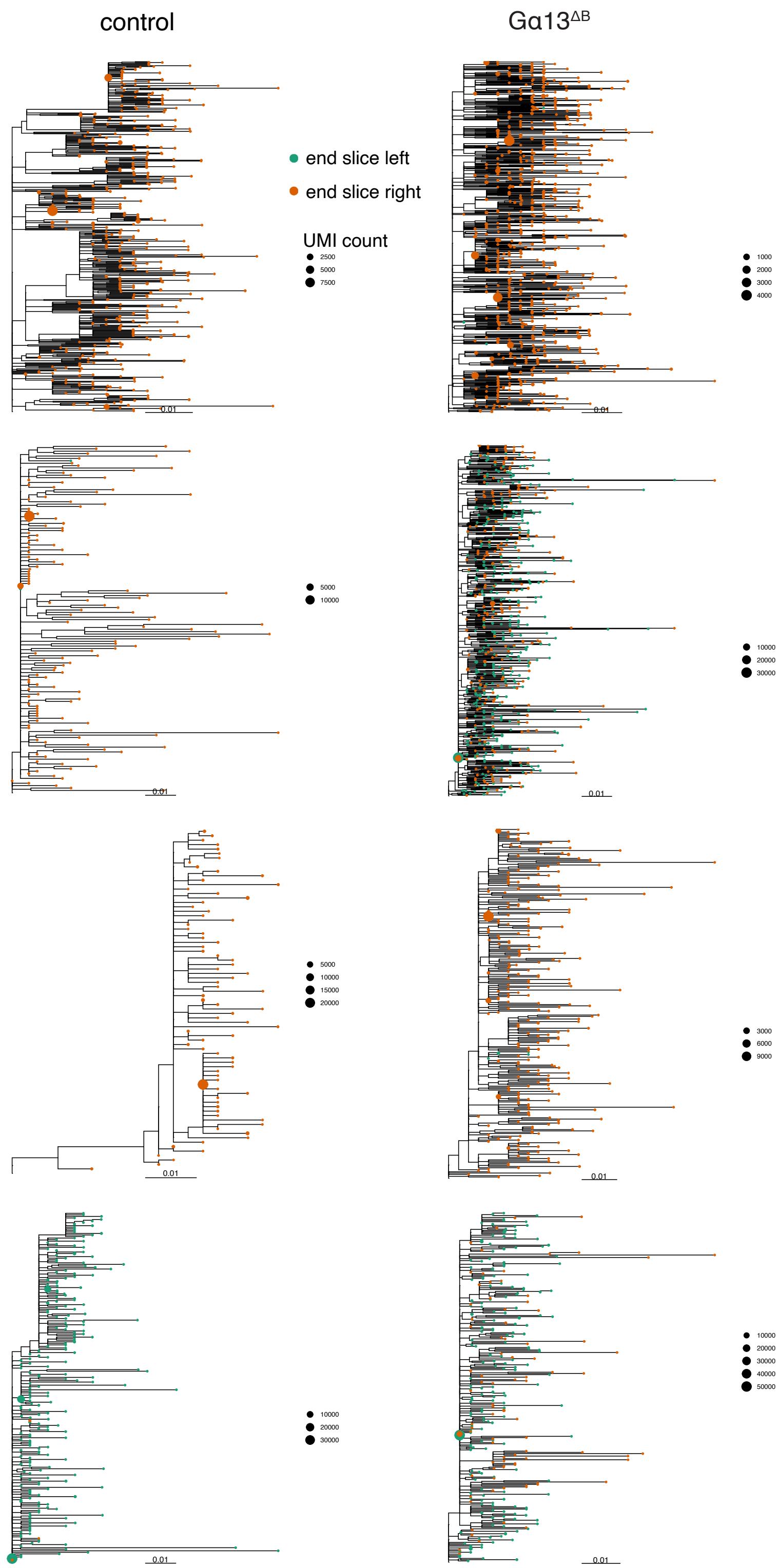

Supplementary Figure 6. Additional lineage trees from WT and Gα13$^{\Delta B}$ LN endpieces.

Additional trees displayed as in Fig. 3d. Each row is a separate LN. Largest clonotype from combined left and right end slices of each LN shown for control (left) and Gα13$^{\Delta B}$ (right) mice.

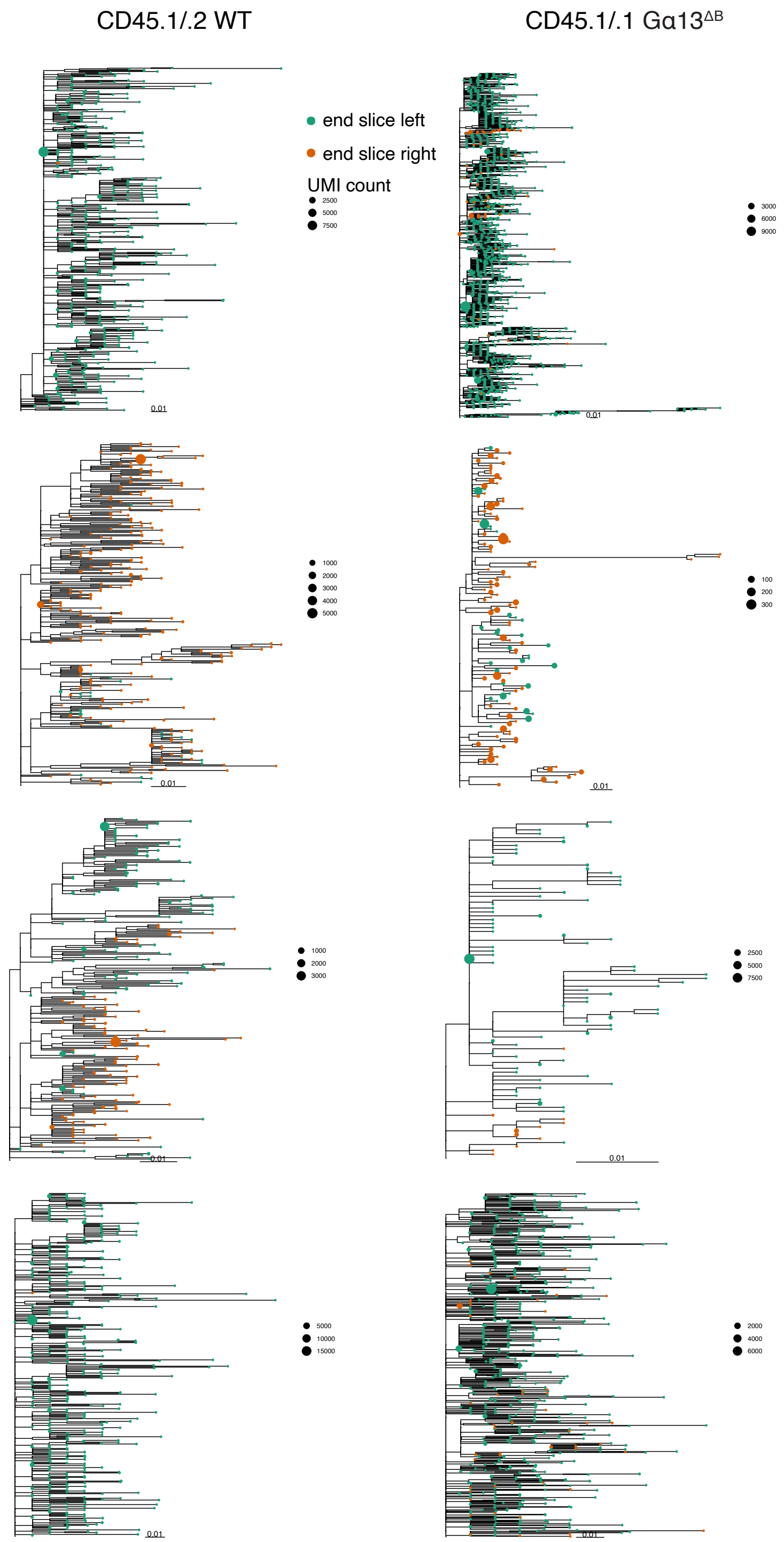

Supplementary Figure 7. Additional sections from micro-dissected WT:Gα13$^{\Delta B}$ mixed chimeras.

Additional trees displayed as in Extended Data Fig. 3c. Each row is a separate LN section. Largest clonotype from combined left and right halves of each section shown for control (left) and Gα13$^{\Delta B}$ (right) genotypes.

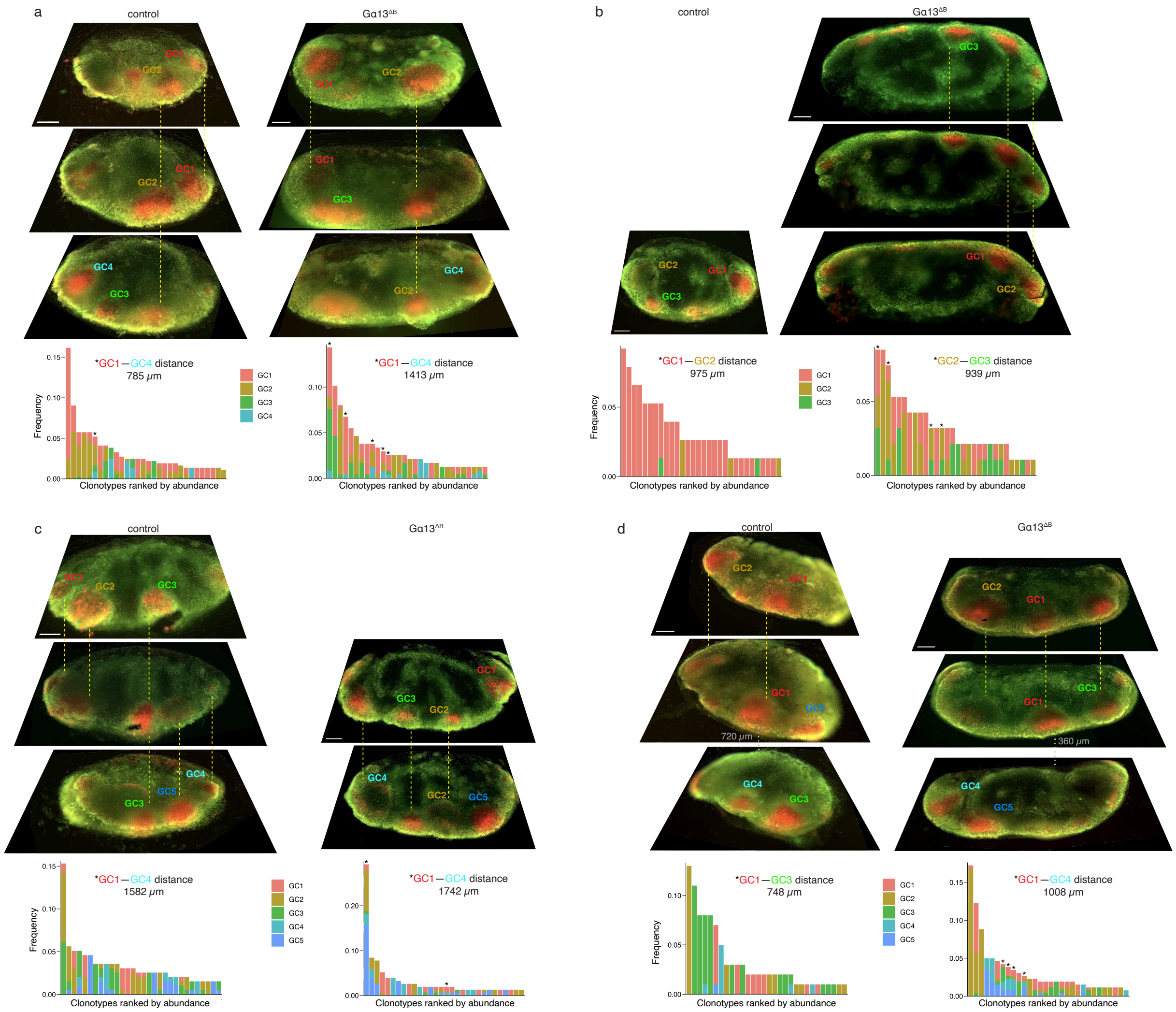

Supplementary Figure 8. Additional LN sections from single-cell ZipSeq.

(a-d) Pairs of section batches from control (left) or Gα13$^{\Delta B}$ LNs (right) processed for ZipSeq, displayed as in Fig. 4a, c. GC text labels are color coded according to histograms below. Dashed yellow lines connect GCs from consecutive slices and a gray dotted line is used for slices that are nearby each other but not consecutive (gap distance indicated). A pair of GCs and their shortest separating distance is noted for each set of sections to highlight two similar pairs between control and Gα13$^{\Delta B}$ mice. Asterisks were placed on top of clonotype bars containing cells from both GCs in the pair. Sections in (a, b) are from *Gna13*$^{fl/+}$ Cre^{Mb1} and *Gna13*$^{fl/fl}$ Cre^{Mb1} mice, and sections in (c, d) are from *Gna13*$^{fl/fl}$ and *Gna13*$^{fl/fl}$ Cre^{Mb1} mice. Number of successfully clonotyped paired heavy and light chain GC B cells with confident zipcode calls from these sets of sections are (a) 791 cells and 710 cells, (b) 174 cells and 251 cells, (c) 540 cells and 452 cells, and (d) 255 and 576 cells, for control and Gα13$^{\Delta B}$, respectively. Scale bars 200 μm.

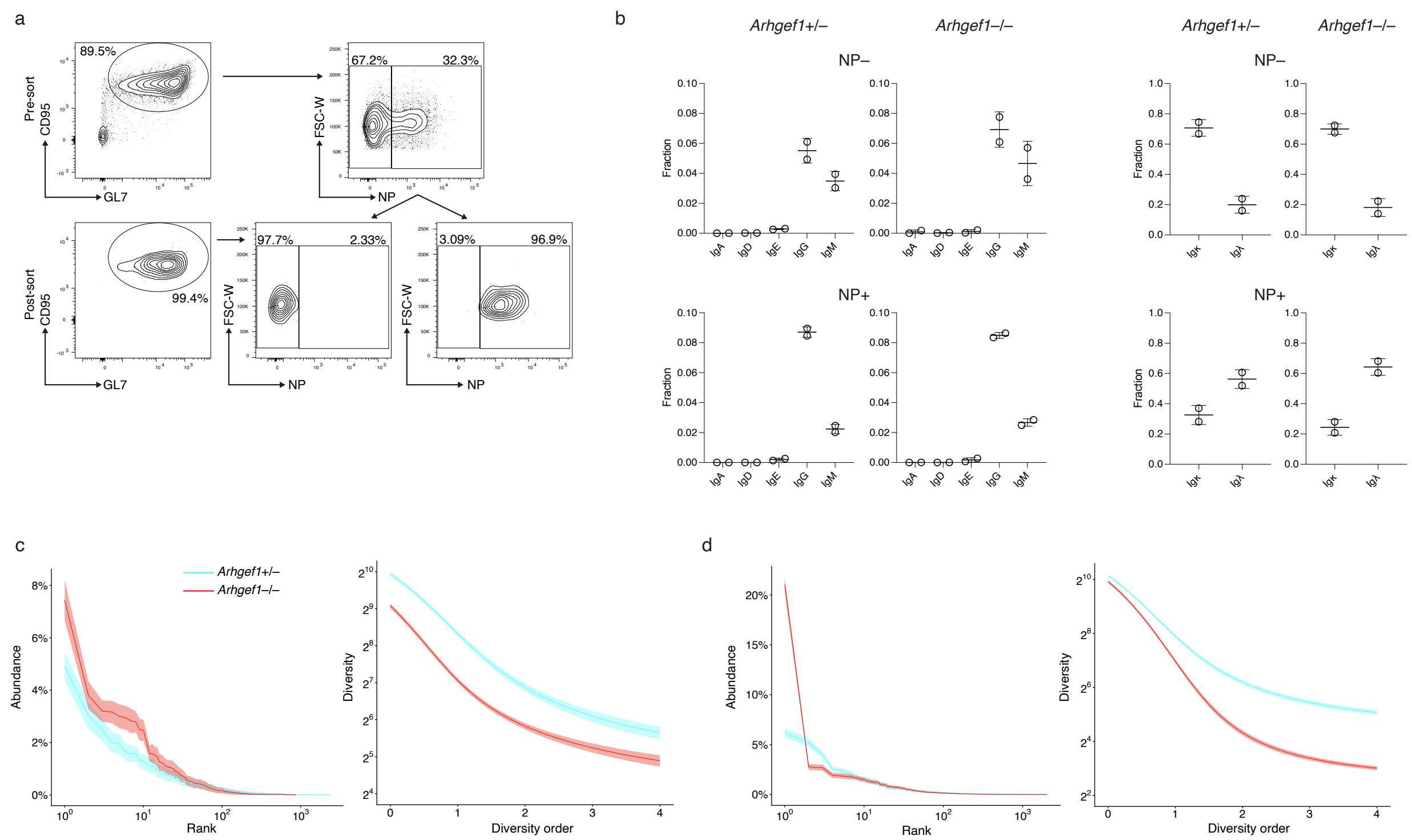

Supplementary Figure 9. Loss of downstream Arhgef1 phenocopies reduction in LN GC diversity.

(a) Example sort gating of NP+ and NP- GC B cells from *Arhgef1*$^{+/-}$ popliteal LNs immunized with NP-KLH in alum 12 days prior. Similar sort gating used for GC B cells from *Gna13*$^{fl/+}$ Cre^{Mb1} and *Gna13*$^{fl/fl}$ Cre^{Mb1} mice in Fig. 5.

(b) Breakdown of heavy and light chain isotypes from bulk sequencing of NP+ and NP- sorted GC B cells from *Arhgef1*$^{+/-}$ and *Arhgef1*$^{-/-}$ popliteal LNs day 12 post NP-KLH in alum. Fractions were determined using the number of UMIs corresponding to each isotype, with light chain isotypes being represented the most due to biased amplification in library preparation. Each datapoint represents a LN. $n = 2$ *Arhgef1*$^{+/-}$ mice, 2 *Arhgef1*$^{-/-}$ mice; two independent experiments.

(c) Rank abundance and Hill diversity curves as in Fig. 5b, c for total LN GC B cells from a pair of mice with low library preparation yield. Shaded error bands represent 95% confidence interval from 100 bootstrapping iterations.

(d) Rank abundance and Hill diversity curves as in Fig. 5b, c for total LN GC B cells from a pair of mice with higher library preparation yield. Shaded error bands represent 95% confidence interval from 100 bootstrapping iterations. For NP- LN samples, 40-50$\times 10^3$ cells were sequenced with ~1$\times 10^6$ read pairs per sample (heavy and light chain), yielding 5-7$\times 10^3$ unique heavy chain sequences and ~1000 clonal groups, with 40-50 clones representing 50% of the population. For NP+ LN samples, 25-40$\times 10^3$ cells were sequenced with ~1$\times 10^6$ read pairs per sample, yielding 2-5$\times 10^3$ unique heavy chain sequences and 300-500 clonal groups, with 10-20 clones representing 50% of the population.

Supplementary Table 1. Barcodes used for single-cell ZipSeq.

ZC1-6 sequence design	GTAGCAACCACAGATCGCACCCGAGAATTCCANNNNNNNNNNCCCATATAAGAAATTT/Cy5/
Partial Read 2 small RNA REV	CACCCGAGAATTC*C*A
TruSeq small RNA REV primer with i7	CAAGCAGAAGACGGCATACGAGNNNNNNNNNNGTGACTGGAGTTCCTTGGCACCCGAGAATTCCA
SI-PCR FWD Primer with i5	AATGATACGGCGACCACCGAGATCTACACNNNNNNNNNNACACTCTTTCCCTACACGACGCTC
Anchor sequence	Same as in Hu et al., 2020 *Nature Methods*
Caged overhang	Same as in Hu et al., 2020 *Nature Methods*

* phosphorothioate bond

Supplementary Table 2. Zipcode and GC correspondence. Zipcode (ZC) number refers to barcode sequences (ZC1-ZC6) listed in GEO accession number GSE314976. GC number (GC1, GC2, etc.) refers to GC labels in the micrographs of the referenced figures (Figure 4 and Supplementary Figure 8). For GCs composed of two ZCs from different slices, 'top', 'middle', and 'bottom' refer to which slice in the referenced figure panels the ZC originates from.

Sample name within GSE314976	Figure reference	GC	ZC
D1_WT_1	Fig. S8a	GC1	ZC1 (top) + ZC3 (middle)
D1_WT_1	Fig. S8a	GC2	ZC2 (top) + ZC4 (middle)
D1_WT_1	Fig. S8a	GC3	ZC5
D1_WT_1	Fig. S8a	GC4	ZC6
D1_KO_1	Fig. S8a	GC1	ZC1 (top) + ZC4 (middle)
D1_KO_1	Fig. S8a	GC2	ZC2 (top) + ZC5 (bottom)
D1_KO_1	Fig. S8a	GC3	ZC3
D1_KO_1	Fig. S8a	GC4	ZC6
D1_WT_2	Fig. S8b	GC1	ZC4
D1_WT_2	Fig. S8b	GC2	ZC5
D1_WT_2	Fig. S8b	GC3	ZC6
D1_KO_2	Fig. S8b	GC1	ZC4
D1_KO_2	Fig. S8b	GC2	ZC5
D1_KO_2	Fig. S8b	GC3	ZC6

D2_WT	Fig. S8d	GC1	ZC1 (top) + ZC5 (middle)
D2_WT	Fig. S8d	GC2	ZC2
D2_WT	Fig. S8d	GC3	ZC3
D2_WT	Fig. S8d	GC4	ZC4
D2_WT	Fig. S8d	GC5	ZC6
D2_KO	Fig. S8d	GC1	ZC1 (top) + ZC3 (middle)
D2_KO	Fig. S8d	GC2	ZC2
D2_KO	Fig. S8d	GC3	ZC4
D2_KO	Fig. S8d	GC4	ZC5
D2_KO	Fig. S8d	GC5	ZC6
D3_WT_1	Fig. 4a	GC1	ZC1 (top) + ZC4 (middle)
D3_WT_1	Fig. 4a	GC2	ZC2 (top) + ZC3 (middle)
D3_WT_1	Fig. 4a	GC3	ZC5
D3_WT_1	Fig. 4a	GC4	ZC6
D3_KO_1	Fig. 4a	GC1	ZC1
D3_KO_1	Fig. 4a	GC2	ZC2 (middle) + ZC4 (top)
D3_KO_1	Fig. 4a	GC3	ZC3 (top) + ZC5 (bottom)
D3_KO_1	Fig. 4a	GC4	ZC6
D3_WT_2	Fig. S8c	GC1	ZC1
D3_WT_2	Fig. S8c	GC2	ZC2
D3_WT_2	Fig. S8c	GC3	ZC3 (top) + ZC6 (bottom)
D3_WT_2	Fig. S8c	GC4	ZC4
D3_WT_2	Fig. S8c	GC5	ZC5
D3_KO_2	Fig. S8c	GC1	ZC1
D3_KO_2	Fig. S8c	GC2	ZC2 (top) + ZC5 (bottom)
D3_KO_2	Fig. S8c	GC3	ZC3
D3_KO_2	Fig. S8c	GC4	ZC4
D3_KO_2	Fig. S8c	GC5	ZC6

Supplementary Methods

Live-cell tracking analysis

For analysis of Cre-ERT2^{S1pr2} *Rosa26*$^{Ai14/+}$ movies, the main GC masses were first masked to focus on tracks outside the GCs. Cells inside the GCs were too dense for single-cell tracking. GC boundaries were determined using the surface function in Imaris, with surface grain size 25 μm, smoothing setting set to enabled, and intensity threshold based on absolute intensity without background subtraction. The intensity threshold was set for each movie such that the main GC aggregates were included but not sparser cells outside. The spots function was then used in Imaris to identify individual cells at every frame in the movie using XY diameter of 9 μm and Z diameter of 15 μm to account of point-spread function spreading in the Z-axis. Imaris-based background subtraction using the input cell diameters was enabled and a quality filter set for each movie to include a majority of objects with GC cell morphology while excluding occasional cell blebs or specks. Spots were then filtered to only include those ≥0 μm from the automatically determined GC borders. Tracks were constructed on these filtered spots using the autoregressive motion algorithm with max step size 20 μm and max frame gap of 3 frames. To exclude less viable Ai14+ cells or occasional Ai14+ non-motile non-GC cells with dendritic stroma-type morphology, tracks were filtered using a minimum threshold on the track speed means and track net displacement. The thresholds were automatically determined by Imaris for each movie using a search algorithm on the 2D histograms. Displacement vectors showing net start and end positions of filtered tracks were then displayed and manually searched for vectors that start and end at or within several μms of GC borders. Due to Ai14 density in the main GC aggregate, single-cell tracks could not start or end inside the GC for many frames. Before vector scoring, automatically determined GC borders were manually re-analyzed. Due to variation in the thickness of LN above certain regions of the movie and fluorescence intensity drop-offs in deeper regions of the movie, the GC boundary was manually redrawn in areas or at timepoints where auto-thresholding was unreliable. For quantification of track properties, all tracks outside the GCs passing the filters above were used for analysis of track speed means and net displacement as determined by Imaris.

Analysis of Cre-ERT2^{Aicda} *Rosa26*$^{Confetti/Confetti}$ movies was carried out similarly to Ai14 explant movies. However, because reporter labelling efficiency was lower in *Rosa26*$^{Confetti/Confetti}$ compared to Ai14, all GC cells were first tracked before inter-GC crossing assessment. Spots labelling Confetti reporter positive GC B cells was performed as described above. RFP channel was used for quantification due to the lower light scattering for longer emission wavelength fluorophores. Tracking was done in Imaris with autoregressive motion algorithm, 15 μm max step size, max gap of 2 frames. Track speed mean and net displacement filters were used as above to gate out non-motile macrophages and less viable cells. Since intensity thresholding could not be used in the same way as in Ai14 to profile GC contours, GC borders were annotated in 3D manually to demarcate regions of high Confetti positive cell density. GC borders were drawn once in the middle of the movie to use for filtering out tracks inside the GCs using a shortest track distance to GC border filter. GC borders were then drawn every 5-10

frames throughout the movie and displacement vectors searched for crossing tracks as done above. RFP channel tracks outside the GCs passing the above filters were then used for quantification of track speed means and net displacement distances. Since not every time lapse was the same duration, only speeds and displacements from 2 hr long movies were pooled. For both Ai14 and Confetti reporter explant movies, mLNs and sub-draining inguinal LNs were harvested in parallel to analyze reporter labelling efficiency. YFP and GFP from Confetti mice were resolved on flow cytometry using spectral unmixing on Cytek Aurora using YFP and GFP single color controls from separate YFP and GFP reporter mice.

Cell morphology and localization analysis in explant imaging

To quantify cell localization, spot objects and tracks were generated for imaged cells using the parameters described above. Cells were further filtered using a combination of track speed, fluorescence intensity in each channel, and estimated cell diameter to exclude artifacts such as auto-fluorescent macrophages. A distance-based filter was applied where any spot >30 µm from the nearest GC surface was defined as being outside the GC. Cell density outside the GC was calculated by determining the total imaged tissue volume using the second-harmonic LN capsule boundary and subtracting the cumulative GC volume. Because cells could be counted inside GCs in Confetti mice, the number of RFP+ cells inside GC aggregates was quantified and, in combination with RFP+ tracks detected as described above, used to estimate the fraction of GC B cells crossing to another GC per hour.

Cell volumes for both GC B cells and naïve B cells were quantified using the surface function in Imaris. Surfaces were generated using intensity-based thresholding followed by manual inspection to ensure accurate segmentation and exclude merged or overlapping cell surfaces. To minimize segmentation artifacts, only the YFP channel was used for surface generation on Confetti cells due to clearer boundary definition and fewer auto-fluorescent objects. Cells outside the GCs were used for volume and sphericity measurements.

Color distribution quantification in Confetti thick sections

For quantification of 2-photon imaged sections of Cre-ERT2^{Aicda} *Rosa26*$^{Confetti/Confetti}$ mice, images were first cropped in 3D in Imaris to focus on the GC of interest. The surfaces function in Imaris was then used to gate on each of the 4 Confetti colors to quantify their representation. A combination of filters was used to obtain color-specific gating. In general, each color was gated using surface smoothing enabled, surface grain size set to 0.8-1.0 µm, background subtraction via local contrast using a cell diameter of 5-8 µm for RFP, YFP and CFP and 3.5-5 µm for nuclear localized GFP, a minimum intensity threshold determined automatically by a 2D histogram search function in Imaris, a minimum voxel count of 100-250 (each voxel 0.509 x 0.509 x 2.50 μm^3), and maximum thresholds on the mean intensity of the surface in the other 3 channels, e.g. CFP, GFP, and YFP max thresholds for RFP surface gating. The minimum intensity threshold helped account for spillover between channels, e.g. exclusion of YFP+ cells in the GFP channel. The voxel and maximum mean intensity thresholds gated out bright specks or second harmonic fibers that were not cells and auto-fluorescent macrophages. The maximum

mean intensity thresholds were determined for each channel and GC using dynamic visualization of excluded objects in Imaris as the threshold was moved. Less frequent double positive cells were observed, such as CFP+ RFP+ or YFP+ RFP+, and max mean intensity thresholds largely included double positive cells in both channels but gated out macrophages bright in every channel. The number of separated surfaces was then quantified in each of the 4 channels to estimate color distribution for each GC. The divergence index for each GC was calculated using the formula $\frac{\sum_1^4 absolute(observed-baseline)}{100}$, where, for each of the 4 colors, "observed" is the percent color of all Confetti positive cells in the imaged GC and "baseline" is the average percent color of all Confetti positive cells in mLNs from 3 mice quantified by flow cytometry.

GC size measurement in LN cross-sections

GC areas in IF cryosections were measured using Fiji. All stitched images were adjusted to consistent brightness and contrast settings prior to analysis. For each GC, boundaries were manually traced based on GL7 and CD21/35 signal three independent times, from which the average area was taken. For consistency, GCs were manually tracked across serial sections, and the largest cross-section was used for analysis. Circularity and roundness were calculated in Fiji using the formulas: Circularity = $\frac{4\pi\times Area}{Perimeter^2}$; Roundness = $\frac{4\times Area}{\pi\times Major\ Axis^2}$.

Bulk BCR repertoire analysis

For *Arhgef1* and some *Gna13* whole LN samples, data was analyzed in the Immcantation suite. UMI-barcoded paired-end sequencing reads were first preprocessed with pRESTO. Reads with mean Phred quality scores less than 20 were removed. PCR primers were masked using Read 1 mouse constant region primers and Read 2 template switch sequences provided by NEB, and reads were annotated with UMI barcodes using the 5'RACE template switch sequences as proxy primers and match error rate to the switch sequences set to 20%. A single consensus sequence was generated for each UMI barcode with removal of UMI read groups having mismatch rates from consensus >0.1 and groups with reads that do not share common primers. Paired-end UMI consensus sequences were assembled into full length Ig sequences followed by additional annotation of the constant region by aligning sequences to isotype-specific internal constant region sequences provided by NEB (max error rate 0.3, match restricted to 100 nucleotides at end of sequence). Duplicate nucleotide sequences sharing the same constant region were then collapsed, generating the UMI count per sequence, and sequences with >20 interior N-valued nucleotides were removed. The preprocessed reads were then aligned to the mouse germline V(D)J genes with the Change-O-IgBLAST pipeline. Data was filtered to include only productive sequences before calculation of UMI count weighted isotype fractions. Data was then filtered for only heavy chain sequences. For clonal clustering, which was performed within individual LNs, the set of distances between each sequence and its closest non-identical neighbor was calculated by grouping sequences with the same V and J gene assignments and CDR3 length followed by finding the minimum junction region mismatch within the group using the single nucleotide Hamming distance metric in SHazaM. This distribution was used to identify

a threshold separating clonal groups within a LN using a density-based method. Hierarchical clonal clustering was then performed using the SCOPer package and the threshold determined above. The clonal relative abundance distributions and clonal Hill diversity curves were then calculated using Alakazam with summed UMI counts as weights for clonal families. To correct for variations in sequencing depth, uniform resampling of the input sequences and recalculation of the clone size distribution and diversity with each resampling realization was performed 100 times.

For other *Gna13* whole LN samples, data was first pre-processed using Cogent Immune Profiler (Takara). Briefly, raw paired-end fastq files were first split by matching reads to different constant chains. Short reads <30 bp were excluded and reads ambiguously matched to multiple constant domains were also removed. The TRUST4 tool was then used to align reads to mouse IMGT reference gene sequences, perform de novo assembly on V, D, J, and C genes, and report consensus contigs of BCR sequences annotated with number of UMI read groups per sequence. Cogent Immune Profiler pre-processing was run with the specification that only UMI groups with at least 2 supporting reads were allowed to contribute to consensus contigs. Following pre-processing, IgG, IgM and IgE sequences were filtered on productive heavy chain reads. Consensus contigs were re-aligned to mouse germline V(D)J genes with the Change-O-IgBLAST pipeline to facilitate integration with the Immcantation framework. Re-alignment results were merged with TRUST4 outputs using sequence identifiers. Sequences with at least 20 Ns or 15 continuous Ns in the V region were then filtered out. Consolidated tables of contigs from each sample were then passed through the clonal clustering process described above, starting with calculation of distance-to-nearest distributions within LNs and visualization of an appropriate clonal clustering threshold based on the distribution. Upon inspection, a Hamming distance threshold of 0.1 was sufficient and used for hierarchical clonal clustering within LNs. From there, UMI count weighted clonal groups were used to estimate relative clonal abundance distributions and Hill diversity curves via bootstrapping in Alakazam as described above. Chord diagrams of V-J segment pairings were visualized for NP+ and NP- pooled GC B cell samples from individual LNs using Cogent Immune Viewer (Takara). For somatic hypermutation analysis, sequences were filtered on IGHV1-72 clones and translated to amino acid strings using the Biostrings package. The minority of IGHV1-72 sequences not starting with glutamine were removed from analysis, and the UMI weighted fractions of sequences with different amino acids at specific positions were calculated.

For LN end slice samples, pre-processing was performed with Cogent IP as described above. IgG, IgM, and IgE consensus contigs were collated for each section and filtered for productive heavy chain sequences. The relative frequency of each consensus contig was calculated using contributing UMI counts, which was passed into analysis with the Immunarch package. Pairwise repertoire overlap was calculated in Immunarch using Morisita's overlap index, with clonal grouping based on identical CDR3 amino acid sequence and V, D and J gene assignment. The Morisita index focuses on dominant clones and was used partly for robustness to variation in sequencing depth between samples. For pie charts of overlapping clones between end slices, shared clones that contained >1000 UMI counts in both slices were color coded. Only the

number of clones covering 70% of the UMI weighted population were displayed in each pie chart. To build lineage trees using the full-length VDJ sequencing data, data from paired end slices was first pooled. Hierarchical clonal clustering was performed within LNs as described above, with thresholds determined using the distance-to-nearest distribution. The germline sequence of the unmutated ancestor of each clonal cluster was reconstructed using the Dowser package in Immcantation, with D and junction regions masked during reconstruction. Clonal clusters were then further processed in Dowser to add left versus right end slice annotations to sequences, mask gap positions, mask ragged ends, collapse and merge UMI counts and annotations of any remaining duplicate sequences (only sequences with identical left or right origin were collapsed), and filter out clonal clusters with less than 3 sequences. B cell phylogenetic trees were then built using the pml program from the phangorn package. The findSwitches function in the Immcantation Docker container was used to enumerate the number of tips per tree and switches from left to right or vice versa along the topology of each tree, and this was used to calculate a switch to tip ratio for trees with at least 20 tips.

For microdissected LN section halves, each sample, representing a knockout or control genotype in a section half, was pre-processed in Cogent IP as described above. IgG and IgM (no IgE for some samples due to lower cell numbers) consensus contigs were collated for each sample, filtered for productive heavy chains, and Morisita's overlap index between corresponding genotypes across section halves was calculated in Immunarch as described for LN end slices. For lineage tree analysis, identical genotypes from each half of a section were grouped together before clonal clustering and tree building. Hierarchical clonal clustering and tree construction were performed as described for LN end slices, with tree annotation using Dowser for left versus right section origin. The switch to tip ratio for trees was also computed as described above.

ZipSeq analyses

Raw bcl files were processed using CellRanger bcl2fastq to separate cDNA, VDJ, and zipcode libraries. cDNA libraries were processed using standard CellRanger count function (v4.0.0) using the reference genome mm10. VDJ libraries were processed using CellRanger v4.0.0 against the reference genome vdj_GRCm38_alts_ensembl-5.0.0. Zipcode libraries were similarly processed using CellRanger with our barcode reference sequences. Before further removal of cells, diversity and λ light chain fractions were analyzed to maximize GC sampling representation. For Simpson's diversity index, the clonotype designations on quality filtered contigs from CellRanger v4.0.0, which groups productively rearranged cells with the same CDR3 amino acid sequence, were used. After excluding cells without an annotated clonotype, the Simpson index, which is less sensitive to rarer species, was calculated using the formula $D = \frac{\sum n_i(n_i-1)}{N(N-1)}$ where n_i is the number of cells in clonotype i and N the total number of cells. The reciprocal was taken so that smaller values correspond to less diversity (more domination from certain clonotypes). To calculate λ light chain fractions, quality filtered contigs from CellRanger vdj were imported into Immcantation to generate AIRR rearrangement data from 10X V(D)J fasta files with re-assigned V, D, and J genes using Change-O and IgBLAST. Cells were then

filtered on VDJ or VJ productive cells and cells with only one heavy and light chain or one light chain were used for calculation of λ cells / λ+κ cells.

For gene expression analysis, cells with a high mitochondrial read count percentage (above 10%) and low nGenes (<500) were filtered out. Individual Seurat objects from each sample were then merged and transformed using SCTransform while regressing out mitochondrial gene percentage. Principal components analysis was performed and the top 15 of these principal components were used to inform the dimensional reduction done by Seurat built-in UMAP function (using the Python implementation of umap-learn package). Meanwhile, nearest neighbor clustering using these principal components was performed using Seurat's built-in FindClusters function at a specified resolution of 0.6. Small clusters of *Cd3e*+ cells and *Lyz2*+ *Ccl5*+ cells, likely myeloid cells, were removed from analysis. To call zipcode identities, zipcode counts were CLR normalized and the ratios of these normalized counts used to gate cells as belonging to one identity or another with ambiguous cells (between gates) filtered from analysis. For differential gene expression analysis, we used Seurat's built-in FindMarkers function that implements testing based on the Wilcoxon rank-sum test along with a Bonferroni correction to adjust p values. The testing was restricted to genes expressed in at least 10% of cells. Genes with $p < 0.05$ and average $\log_2$ fold change > 0.2 were deemed significant. For calling clonotypes and constructing lineages, we used the enclone module, which groups cells into clonotypes based on germline allele assignments, CDR3 length, CDR3 nucleotide identity, shared somatic hypermutations, and light chain constant regions. For each sample, the CellRanger output files all_contig_annotations.json and vdj_reference/fasta/regions.fa were used as input. Clonotype designations were then integrated into the metadata of the Seurat object generated above. Phylogenetic trees were also calculated using enclone in the Newick format.

For spatial clonal sharing analysis, cells with only light chains were filtered out and a minor population of *sdc1*+ cells, likely pre-plasma cells, were also removed. After clonotype grouping, integrated zipcode and VDJ Seurat objects were used to build clonotype phylogenetic trees and histograms of the clonotype zipcode distributions, with cells with ambiguous constant chain removed. Zipcodes corresponding to the same GCs across serial sections were combined according to Supplementary Table 2. For overlap enrichment versus inter-GC distance analysis, overlap index was calculated using the formula

$$Overlap\ index = (f_{GCn\ AND\ GCm})/(f_{GCn} \times f_{GCm})$$

where, for a set of clonotypes from a batch of serial sections, f is the fraction of clonotypes in GC_n, GC_m, or both. Clonotypes with at least 3 cells were included in the calculation. The index indicates the enrichment of the observed overlap over the theoretical overlap if clonotypes were randomly scattered across GCs. For instance, if half of all considered clonotypes were found in GC1 and half of all considered clonotypes were found in GC2, and GCs were independent of each other, we would expect ½ * ½ = ¼ of all clonotypes to be found in both GC1 and GC2 by chance. Therefore, if we observe ¼ of all clonotypes have both GC1 and GC2 assignments, the overlap index would be ¼ / ¼ = 1. Most GC pairs have overlap index <1, indicating less overlap

than expected by chance, due to biological bias against GC clonal sharing. One extreme would be every clonotype in GC1 is also found in GC2. This would result in ½ / ¼ = 2, representing greater overlap than expected by chance. Another extreme would be no clonotypes are shared between GC1 and GC2, resulting in overlap index 0 / ¼ = 0. Inter-GC distance was measured in Fiji by finding the shortest border-border distance between photo-uncaging regions. If two GCs were on separate consecutive slices, the slice thickness (180 μm) and minimum projected horizontal distance was used to calculate the shortest hypotenuse between the GCs. Only GC pairs on the same or consecutive slices were analyzed and overlap enrichments for all possible GC pairs were then plotted against separating distance.

To calculate jump distance/node or number for jumps/node, Newick trees were loaded from enclone for each complete clonotype (clonotypes with both heavy and light chain) with at least 3 cells. We then assigned each tree leaf (individual cells) their GC designation. We then used the parsimony function from phangorn and the ‘fitch’ method to calculate the minimum number of jumps between GCs necessary to achieve the resulting tree. The number of jumps was then normalized to the number of nodes, or branchpoints, in each clonotype tree. For total jump distance, we then weighted each jump by the inter-GC distance calculated above and summed these values. In certain instances, two jumping models with equal likelihoods and parsimony were found, in which case we took an even average of the distances from either possibility. The summed distances were then normalized to number of nodes. For filtering of early jump events, the GC assignments of inferred common ancestor nodes were first determined using Fitch parsimony to minimize the number of jumps needed to achieve the observed tree pattern. A difference in assigned GC identity between a parent node and a child node was denoted as a GC-to-GC jump. Each jump event was assigned a distance from the root as defined by the enclone computed Levenshtein distance at the parent node. We then filtered out any jumps that occurred within a distance of 1 from the inferred tree root. For analysis of SHM positions, we tabulated all pairs of ZipSeq GC B cells that were neighbors on the clonotype lineage tree, had different GC origins, shared at least one SHM, and had a common ancestor within 2 inferred nodes upstream. The fraction of pairs that shared at least one SHM in a framework region or at least two SHMs in CDR regions was then calculated.

1 Germinal Centre Simulation Model

The germinal center (GC) model assumptions underlying the GC simulations are explained here and include the used parameter values. It follows [1] with additional features [2, 3, 4], and adding the specificities of the DisseD model from [5]. Used acronyms are: DZ for dark zone, LZ for light zone, Tfh for T follicular helper cell, FDC for follicular dendritic cell, BC for B cell, BCR for B cell receptor.

1.0.1 Space representation

All reactions take place on a three-dimensional discretized space with a rectangular lattice with lattice constant of $\Delta x = 5\mu m$. Every lattice node can be occupied by a single cell only.

1.0.2 Shape space for antibodies

Antibodies are represented on a four ($d = 4$) dimensional shape space [6]. The shape space is restricted to a size of 10 positions per dimension, thus, only considering antibodies with a minimum affinity to the antigen. The optimal clone Φ^* is positioned in the center of the shape space. A position on the shape space Φ is attributed to each BC.

The 1-Norm with respect to the optimal clone $||\Phi - \Phi^*||_1 = \sum_{i=1}^{d} |\Phi_i - \Phi_i^*|$, i.e. the minimum number of mutations required to reach the optimal clone, is used as a measure for the antigen binding probability. The binding probability is calculated from the Gaussian distribution with width $\Gamma = 2.8$ [7]:

$$b\left(\Phi, \Phi^*\right) \quad = \quad \exp\left(-\frac{||\Phi - \Phi^*||_1^2}{\Gamma^2}\right) \quad . \tag{1}$$

1.0.3 B cell phenotypes

Three BC phenotypes are distinguished: DZ BCs, LZ BCs, and output cells. The different phenotypes characterize the cell properties and are not meant as localization within the GC zones. DZ BCs divide, mutate and migrate. LZ BCs also migrate and undergo the different stages of the selection process. Output cells only migrate.

1.0.4 Founder cells

The model starts with 150 Tfh, 200 FDCs, 300 stromal cells, and no BC. Tfh are randomly distributed on the lattice and occupy a single node each. Stromal cells are restricted to the DZ (see section *Chemokine distribution* for their function). FDCs are restricted to the upper half of the reaction sphere, occupy one node by their soma and have 6 dendrites of $40\mu m$ length each. The presence of dendrites is represented as a lattice-node property and, thus, visible to BCs. The dendrites are treated as transparent for BC or Tfh migration such that they do not inhibit cell motility.

BCs enter the GC reaction with a probability per time step corresponding to a rate of 2 cells per hour (for an estimation of this value refer to [4]). New BCs are randomly positioned on free lattice nodes. The shape space position of each new BC is randomly chosen but restricted to positions with a distance of at least 3 from the optimal clone.

1.0.5 Antigen-presentation by FDCs

Each FDC is loaded with 10000 antigen portions distributed onto the lattice-nodes occupied by FDC-soma or FDC-dendrite unless stated otherwise. One antigen portion corresponds to the number of antigen molecules taken up by a BC upon successful contact with an FDC.

1.0.6 DZ B cell division

The average cell cycle duration of 9.5 hours of DZ BCs is varied for each BC according to a Gaussian distribution. This is needed to get desynchronization of BC division. The cell cycle is decomposed into four phases (G1, S, G2, M) in order to localize mitotic events if this is needed.

Each founder BC divides a number of times before differentiating to the LZ phenotype for the first time. Six divisions was the number of divisions found in response to the extreme stimulus with

anti-DEC205-OVA [8, 1]. Each selected BC divides a number of times determined by the interaction with Tfh (see below, LZ B cell selection). The parameters of the interaction with Tfh are tuned such that the mean number of divisions is in the range of two [9]. This value is required in order to maintain a DZ to LZ ratio in the range of two [8, 1].

A division requires free space on one of the Moore neighbors of the dividing cell. Otherwise the division is postponed until a free Moore neighbor is available.

At every division the encoded antibody mutates with probability 0.7 corresponding to a mutation rate of 1 mutation per 1000 base pair per divisions [10]. Each mutation corresponds to a shift in the shape space to a von Neumann neighbor in a random direction. Upon selection by Tfh cells the mutation probability is individually reduced from $m_{\max}$ down to $m_{\min} = 0$ in an affinity-dependent way following

$$m(b) = m_{\max} - (m_{\max} - m_{\min})b = \frac{1-b}{2} \tag{2}$$

with b from Equation 1. Thus, after recycling, DZ BCs can acquire reduced mutation probabilities. This mechanism is motivated by the observation that BCR internalization enhances the activation of the kinase Akt [11]. which, in turn, suppresses activation induced cytosine deaminase (AID) ([12]). AID is required for somatic hypermutation, such that this provides an affinity-dependent downregulation of the mutation frequency ([13]). BC division of BCs that previously acquired antigen and have been selected by Tfh cells distribute the retained antigen asymmetrically to the daughters ([14]). The model assumes asymmetric division in 72% of the cases, which is supported by experimental observations (see [14] and Figure S1 in [1]). If division is asymmetric, one daughter gets all the retained antigen while the other gets none, which approximates the value of 88% found in ([14]. Mutation is suppressed in cells retaining antigen.

After the required number of divisions the BC differentiates with a rate of in 1/6 minutes to the LZ phenotype. All BCs that kept the antigen up to this time, differentiate to output cells, up-regulate CXCR4, and leave the GC in direction of the T zone.

1.0.7 LZ B cell selection

At the time $t = 0$ of differentiation from the DZ to the LZ phenotype, BCs are in state *unselected* and BC-specific factors are initialized to

$$\begin{aligned} F(t=0) &= F_0 = 1 \\ C(t=0) &= C_0 = 0 \\ R(t=0) &= R_0 = 0 \end{aligned} \tag{3}$$

These factors are associated with FoxO1 (F), c-Myc (C), and mTOR (R) in the model. In BCs not in contact to Tfh, FoxO1, mTOR and c-Myc evolve according to

$$\frac{dF}{dt} = -\alpha_{\mathrm{F}} H_{\mathrm{F}}(B)\, F \tag{4}$$

$$\frac{dR}{dt} = \beta_{\mathrm{R}} H_{\mathrm{R}}(B) \tag{5}$$

$$\frac{dC}{dt} = -\gamma_{\mathrm{C}} C \quad , \tag{6}$$

with $\beta_{\mathrm{R}} = 1/(8\,\text{hours})$ the mTOR production rate modulated by the BCR-dependent Hill-function $H_{\mathrm{R}}(B)$ with K-value of 20 and Hill-coefficient 2. $\alpha_{\mathrm{F}} = 2/\text{hours}$ is the FoxO1 reduction rate modulated by the BCR-dependent Hill-function $H_{\mathrm{F}}(B)$ between 0 and 1 with K-value of 20 and Hill-coefficient 1. $\gamma_{\mathrm{C}} = \ln(2)/(3\,\text{hours})$ is the c-Myc degradation rate. R and C have different dynamics in state *Tfh-contact.*

LZ BCs can be in the states *unselected, FDC-contact, FDC-selected, Tfh-contact, selected, apoptotic.*

1.1 Unselected

LZ BCs migrate and search for contact with FDCs loaded with antigen in order to collect antigen for 0.7 hours. If an FDC soma or dendrite is present at the position of the BC, the BC attempts

to establish contact to the epitope with highest affinity to the BCR (default setting). Alternatively, the BC may attempt to establish contact to the epitope of highest availability at this site. In both settings, binding is affinity dependent and happens with the probability b in Eq. (1). If the available number of antigen portions at the specific FDC site drops below 20 the binding probability b is linearly reduced with the number of available portions. If successful, the BC switches to the state *FDC-contact*; otherwise the BC continues to migrate. Further binding-attempts are prohibited for 1.2 minutes. At the end of the antigen collection period, BCs switch to the state *FDC-selected*. If a LZ BC fails to collect any antigen until this time it switches to the state *apoptotic*.

1.2 FDC-contact

LZ BCs remain immobile (bound) for 3 minutes [15] and then return to the state *unselected* or *FDC-selected*, depending on from which state contact to FDC was established. The counter for the number of successful antigen uptake events p and the degree of BCR-signaling B are increased by one unit and the FDC reduces its locally available antigen portions by one. Also, BC-sepcific factors mentiond before depend on the amount of presented pMHC p and the degree of BCR-signaling B.

1.3 FDC-selected

BCs search for contact with Tfh or FDC. If they meet a Tfh they switch to the state *Tfh-contact*. If they meet a FDC they switch to the state *FDC-contact* with probability b in Eq. (1). If Tfhs and FDCs are neighbors of a BC, the BC binds the Tfh.

1.4 Tfh-contact

LZ BCs remain immobile for a time determined by a Hill-function in dependence on the amount of presented pMHC p. This hill function has values between 0 and 0.2, K-value 15 and a Hill-coefficient of 2. During this time, the bound Tfh, which may also be bound to other BCs, polarizes to the BC with highest number of successful antigen uptakes. Polarization is determined from scratch in every time step and inertia from repolarization or intracellular organelles are ignored. If more than one BC shares the same highest pMHC presentation p, polarization is chosen randomly among those BCs. Only the BC to which the Tfh is polarized receives Tfh signals C and accumulate those.

Tfh signals are represented as mTOR levels as well as c-Myc levels, which are modulated by FoxO1, according to

$$\frac{dC}{dt} = \alpha_{\mathrm{C}} \left(1 - H_{\mathrm{C}}(F)\right) T(p) H_{\mathrm{I}}(t) \delta_{\mathrm{TB}} - \gamma_{\mathrm{C}} C \tag{7}$$

$$\frac{dR}{dt} = \alpha_{\mathrm{R}} H_{\mathrm{R}}\left(B\right) H_{\mathrm{I}}(t) T(p) \delta_{\mathrm{TB}} + \beta_{\mathrm{R}} H_{\mathrm{R}}\left(B\right) \quad . \tag{8}$$

$T(p)$ is the pMHC-dependent intensity of Tfh signaling to the interacting BC associated with CD40-signals, which is described by a Hill-function with values between zero and three, K-value 15 and a Hill-coefficient of 2. Signaling intensity is further modulated by the degree of ICOSL upregulation $H_{\mathrm{I}}(t)$. $H_{\mathrm{I}}(t)$ is a Hill-function in time with values between zero and one, determined by a characteristic ICOSL-upregulation time of four hours in mice [16] and Hill-coefficient two. α_{C} and γ_{C} are the growth and degradation rates of c-Myc. $\beta_{\mathrm{R}} = 1/(8\,\text{hours})$ tunes the level of mTOR upregulation when BCs are not in contact to Tfh (see Eq. (5)), while $\alpha_{\mathrm{R}} = 1/(\text{hours})$ determines mTOR upregulation during productive contact with Tfh. $H_{\mathrm{R}}(B)$ is a Hill-function describing the dependence of mTOR regulation on signals downstream of BCR. $H_{\mathrm{C}}(F)$ is a Hill-function controlling the suppression of c-Myc signals by FoxO1 with values between zero and one, K-value of 0.5 and Hill-coefficient of 1.

After the binding time, the BC detaches and returns to the state *FDC-selected*. It continues to search for and bind FDC or Tfhs, where binding twice the same Tfh in a sequence is excluded. The total period of signal acquisition in the LZ is finished when the LZ passage time gets longer than the number of antigen uptake events times 0.5 hours. When this limit is reached during a running contact, the contact is kept active until the end of the contact time. Then, it switches to the state *apoptotic* if either $R < 1$ or if the received Tfh-signals are $C < 0.3$. It switches to the state *selected* otherwise.

1.5 Selected

LZ BCs keep the LZ phenotype for 6 hours and desensitize for CXCL13, thus, perform a random walk. During that time they re-enter cell cycle and progress through the cell cycle phases. Then they recycle back to the DZ phenotype with a rate of 1/6 minutes and memorize the amount of collected antigen as well as the cell cycle phase they have achieved by this time.

The number of divisions $P(C)$ the recycled BCs will do is derived from the C, which reflects the c-Myc level, according to

$$P(C) = \frac{\gamma(\alpha, \beta C)}{\Gamma(\alpha)} \cdot r + 1 \,. \tag{9}$$

where r is a rescaling factor, α and β are the shape and scale parameters of the incomplete gamma function given by

$$\gamma(\alpha, \beta C) = \int_{\beta C}^{\infty} t^{\alpha-1} e^{-t} dt \tag{10}$$

To fit the experimental observations we put r to 31, α to 5 and β to 0.4. Reproducing an average number of two divisions per dark zone passage and a maximum number of divisions >= 10 for the observed c-Myc levels.

1.6 Apoptotic

LZ BCs remain on the lattice for 6 hours before they are deleted. They continue to be sensitive to CXCL13 during this time.

1.6.1 Chemokine distribution

Two chemokines CXCL12 and CXCL13 are considered. CXCL13 is produced by FDCs in the LZ with 10nMol per hour and FDC while CXCL12 is produced by stromal cells in the DZ with 400nMol per hour and stromal cell. As both cell types are assumed to be immobile, chemokine distributions were pre-calculated once and the resulting steady state distributions were used in all simulations.

1.6.2 Chemotaxis

DZ and LZ BCs regulate their sensitivity to CXCL13 and CXCL12, respectively. This is true in all BC states unless stated otherwise. All BCs move with a target speed of $7.5 \mu m/min$. This leads to a slightly lower observable average speed of $\approx 6 \mu m/min$.

BCs have a polarity vector that determines their preferential direction of migration. The polarity vector $\vec{p}$ is reset every 1.5 minutes into a new direction using the chemokine distribution c as

$$\vec{p} = \vec{p}_{\rm rand} + \frac{\alpha}{1 + \exp\left\{\kappa \left(K_{1/2} - \Delta x |\vec{\nabla} c|\right)\right\}} \frac{\vec{\nabla} c}{|\vec{\nabla} c|} \quad , \tag{11}$$

where $\vec{p}_{\rm rand}$ is a random polarity vector and the turning angle is sampled from the measured turning angle distribution ([17] Fig. S1B). $\alpha = 3$ determines the relative weight of the chemotaxis and random walk, $K_{1/2} = 2 \cdot 10^{11}$ Mol determines the gradient of half maximum chemotaxis weight, and $\kappa = 10^{10}$/Mol determines the steepness of the weight increase.

BCs de- and re-sensitize for their respective chemokine depending on the local chemokine concentration: The desensitization threshold is set to 4.5nMol and 0.08nMol for CXCL12 and CXCL13, respectively, which avoids cell clustering in the center of the zones. The resensitization threshold is set at 2/3 and 3/4 of the desensitization threshold for CXCL12 and CXCL13, respectively.

BCs can only migrate if the target node is free. If occupied and the neighbor cell is to migrate in the opposite direction (negative scalar product of the polarity vectors) both cells are exchanged with a probability of 0.5. This exchange algorithm avoids lattice artifacts leading to cell clusters.

Tfh do random walk with a preferential directionality to the LZ: The polarity vector $\vec{p}$ of Tfh is determined from a mixture of random walk $\vec{r}$ and the direction of the LZ $\vec{n}$ by

$$\vec{p} = (1 - \alpha')\vec{r} + \alpha' \vec{n} \,, \tag{12}$$

where $\alpha' = 0.1$ is the weight of chemotaxis. This weight leads to a dominance of random walk with a tendency to accumulate in the LZ as found in experiment. TCs migrate with an average speed of $10\mu m/min$ and repolarize every 1.7 minutes [18].

Output cell motility is derived from plasma cell motility data to 3μm per minute with a persistence time of 0.75 minutes [17].

1.7 Intra-GC transmigration

B cells may transmigrate every 54 s, probability of which depends on the assumed per-cell exchange rate. Only B cells that are not in state *apoptotic*, *FDC-contact* or *Tfh-contact* may transmigrate. Transmigration is restricted to GCs within the same lymph node (LN), with each LN comprising eight GCs. Spatially, GCs within a LN are arranged on a spherical shell of radius 500 µm, with a minimum inter-GC distance of 150 µm between GC borders (GC radius $r_{\text{GC}} = 160\,\mu\text{m}$). The destination GC for each transmigrating B cell is selected uniformly at random, and migration is assumed to proceed along the shortest path between GC borders at a speed of $10\,\mu\text{m}\,\text{min}^{-1}$ [19]. Cellular state variables are assumed to remain unchanged during transmigration. Upon arrival, transmigrated B cells are placed uniformly at random on border grid points of the corresponding target zone.

1.8 Brainbow experiment

In Brainbow experiments [20], each GC B cell is assigned one of four colours (or left unlabelled) using empirically estimated staining probabilities. Labels are maintained throughout the simulation, including during transmigration, and are passed on to daughter cells upon cell division.

1.9 Code availability

Simulation code and statistical analysis code is deposited on zotero along with the simulation outputs `doi.org/10.5281/zenodo.21515363`.

References

[1] Meyer-Hermann M, Mohr E, Pelletier N, Zhang Y, Victora GD, Toellner KM. A theory of germinal center B cell selection, division, and exit. Cell reports. 2012;2(1):162-74.

[2] Meyer-Hermann M. Overcoming the dichotomy of quantity and quality in antibody responses. The Journal of Immunology. 2014;193(11):5414-9.

[3] Binder SC, Meyer-Hermann M. Implications of intravital imaging of murine germinal centers on the control of B cell selection and division. Frontiers in immunology. 2016;7:593.

[4] Meyer-Hermann M. Injection of antibodies against immunodominant epitopes tunes germinal centers to generate broadly neutralizing antibodies. Cell reports. 2019;29(5):1066-73.

[5] Meyer-Hermann M. A molecular theory of germinal center B cell selection and division. Cell Reports. 2021;36(8).

[6] Perelson AS, Oster GF. Theoretical studies of clonal selection: minimal antibody repertoire size and reliability of self-non-self discrimination. Journal of theoretical biology. 1979;81(4):645-70.

[7] Meyer-Hermann M, Deutsch A, Or-Guil M. Recycling probability and dynamical properties of germinal center reactions. Journal of Theoretical Biology. 2001;210(3):265-85.

[8] Victora GD, Schwickert TA, Fooksman DR, Kamphorst AO, Meyer-Hermann M, Dustin ML, et al. Germinal center dynamics revealed by multiphoton microscopy with a photoactivatable fluorescent reporter. Cell. 2010;143(4):592-605.

[9] Gitlin AD, Shulman Z, Nussenzweig MC. Clonal selection in the germinal centre by regulated proliferation and hypermutation. Nature. 2014;509(7502):637-40.

[10] Pae J, Schwan N, Ottino-Loffler B, DeWitt WS, Garg A, Bortolatto J, et al. Transient Silencing of Hypermutation Preserves B Cell Affinity during Clonal Bursting.

[11] Chaturvedi A, Martz R, Dorward D, Waisberg M, Pierce SK. Endocytosed BCRs sequentially regulate MAPK and Akt signaling pathways from intracellular compartments. Nature immunology. 2011;12(11):1119-26.

[12] Omori SA, Cato MH, Anzelon-Mills A, Puri KD, Shapiro-Shelef M, Calame K, et al. Regulation of class-switch recombination and plasma cell differentiation by phosphatidylinositol 3-kinase signaling. Immunity. 2006;25(4):545-57.

[13] Dustin ML, Meyer-Hermann M. Antigen feast or famine. Science. 2012;335(6067):408-9.

[14] Thaunat O, Granja AG, Barral P, Filby A, Montaner B, Collinson L, et al. Asymmetric segregation of polarized antigen on B cell division shapes presentation capacity. Science. 2012;335(6067):475-9.

[15] Schwickert TA, Lindquist RL, Shakhar G, Livshits G, Skokos D, Kosco-Vilbois MH, et al. In vivo imaging of germinal centres reveals a dynamic open structure. Nature. 2007;446(7131):83-7.

[16] Papa I, Saliba D, Ponzoni M, Bustamante S, Canete PF, Gonzalez-Figueroa P, et al. TFH-derived dopamine accelerates productive synapses in germinal centres. Nature. 2017;547(7663):318-23.

[17] Allen CD, Okada T, Tang HL, Cyster JG. Imaging of germinal center selection events during affinity maturation. Science. 2007;315(5811):528-31.

[18] Miller MJ, Wei SH, Parker I, Cahalan MD. Two-photon imaging of lymphocyte motility and antigen response in intact lymph node. Science. 2002;296(5574):1869-73.

[19] Meyer-Hermann ME, Maini PK. Interpreting Two-Photon Imaging Data of Lymphocyte Motility;71(6):061912. Available from: `https://link.aps.org/doi/10.1103/PhysRevE.71.061912`.

[20] Meyer-Hermann M, Binder SC, Mesin L, Victora GD. Computer Simulation of Multi-Color Brainbow Staining and Clonal Evolution of B Cells in Germinal Centers;9:2020. Available from: `https://www.frontiersin.org/article/10.3389/fimmu.2018.02020/full`.
