# Supplementary material for: Spatial confinement promotes germinal center diversity and tempers immunodominance
Source: Nat Immunol. Author manuscript; Available in PMC 2026 Oct 30. (PMC13720956; doi:10.1038/s41590-026-02698-x)
Supplement: Supplementary file 2 [file NIHMS2214944-supplement-Supplemental_Movie_Legends.docx]

**Supplemental Movie Legends**

**Movie S1.**

Multiphoton imaging of *Gna13*^fl/+^ Cre-ERT2^S1pr2^ *Rosa26*^Ai14/+^ popliteal LN, related to Fig. 1. A 65 μm stack was obtained at 38 second intervals for 45 min. Ai14+ cells are in orange and second harmonic in green. Gray spheres and trailing gray lines (past 350 seconds of track) mark cells tracked outside or near the GC borders, with static or low displacement cells filtered from tracking. 50 μm scale bar and time in min:sec indicated on bottom.

**Movie S2**

Multiphoton imaging (65 μm stack) of another *Gna13*^fl/+^ Cre-ERT2^S1pr2^ *Rosa26*^Ai14/+^ mouse, displayed as in Movie S1. Stacks were acquired for 45 min at 38 second intervals. 50 μm scale bar and time in min:sec indicated on bottom.

**Movie S3**

Multiphoton imaging (65 μm stack) of another *Gna13*^fl/+^ Cre-ERT2^S1pr2^ *Rosa26*^Ai14/+^ mouse, displayed as in Movie S1. Stacks were acquired for 45 min at 38 second intervals. 50 μm scale bar and time in min:sec indicated on bottom.

**Movie S4**

Multiphoton imaging (65 μm stack) of a *Gna13*^fl/fl^ Cre-ERT2^S1pr2^ *Rosa26*^Ai14/+^ mouse, displayed as in Movie S1. Stacks were acquired for 45 min at 38 second intervals. Tracks of cells scored as trafficking from one GC to another highlighted in yellow. 50 μm scale bar and time in min:sec indicated on bottom.

**Movie S5**

Multiphoton imaging (65 μm stack) of another *Gna13*^fl/fl^ Cre-ERT2^S1pr2^ *Rosa26*^Ai14/+^ mouse, displayed as in Movie S4. Stacks were acquired for 45 min at 38 second intervals. 40 μm scale bar and time in min:sec indicated on bottom.

**Movie S6**

Multiphoton imaging (65 μm stack) of another *Gna13*^fl/fl^ Cre-ERT2^S1pr2^ *Rosa26*^Ai14/+^ mouse, displayed as in Movie S4. Stacks were acquired for 45 min at 38 second intervals. 50 μm scale bar and time in min:sec indicated on bottom.

**Movie S7.**

Multiphoton imaging of *Arhgef1*^+/-^ Cre-ERT2^Aicda^ *Rosa26*^Confetti/Confetti^ popliteal LN, related to Extended Data Figure 2A-F. A 60 μm stack was obtained at 35 second intervals for 117 min. RFP, YFP, GFP, and CFP are in red, green, cyan, and blue, respectively, and second harmonic in blue as well. Macrophages autofluorescent in multiple channels are also visible. Both RFP and YFP channels tracked in this movie but only RFP used for quantification in Extended Data Figure 2D-F. Gray spheres and trailing gray lines track cells as in Movie S1. White dashed borders highlight initial GC regions. 50 μm scale bar and time in min:sec indicated on bottom.

**Movie S8.**

Multiphoton imaging (60 μm stack) of another *Arhgef1*^+/-^ Cre-ERT2^Aicda^ *Rosa26*^Confetti/Confetti^ mouse, displayed as in Movie S7. Stacks were acquired for 117 min at 35 second intervals. Only RFP channel used for tracking, with some YFP+ cells tracked due to YFP+ RFP+ double positives or fluorescence spillover. 50 μm scale bar and time in min:sec indicated on bottom.

**Movie S9.**

Multiphoton imaging (65 μm stack) of another *Arhgef1*^+/-^ Cre-ERT2^Aicda^ *Rosa26*^Confetti/Confetti^ mouse, displayed as in Movie S7. Stacks were acquired for 45 min at 38 second intervals. Only RFP channel used for tracking. 50 μm scale bar and time in min:sec indicated on bottom.

**Movie S10.**

Multiphoton imaging (60 μm stack) of an *Arhgef1*^-/-^ Cre-ERT2^Aicda^ *Rosa26*^Confetti/Confetti^ mouse, displayed as in Movie S7. Stacks were acquired for 117 min at 35 second intervals. Only RFP channel used for tracking. Tracks of cells scored as crossing GCs highlighted in yellow. 50 μm scale bar and time in min:sec indicated on bottom.

**Movie S11.**

Multiphoton imaging (60 μm stack) of another *Arhgef1*^-/-^ Cre-ERT2^Aicda^ *Rosa26*^Confetti/Confetti^ mouse, displayed as in Movie S7. Stacks were acquired for 117 min at 35 second intervals. Only RFP channel used for tracking. 50 μm scale bar and time in min:sec indicated on bottom.

**Movie S12.**

Multiphoton imaging (65 μm stack) of another *Arhgef1*^-/-^ Cre-ERT2^Aicda^ *Rosa26*^Confetti/Confetti^ mouse, displayed as in Movie S7. Stacks were acquired for 45 min at 38 second intervals. Only RFP channel used for tracking. 50 μm scale bar and time in min:sec indicated on bottom.

**Movie S13.**

Multiphoton imaging (60 μm stack) of a *Arhgef1*^+/–^ Cre-ERT2^Aicda^ *Rosa26*^Confetti/Confetti^ popliteal LN, related to Extended Data Figure 2G, H. Stacks were acquired for 117 min at 35 second intervals. Cells were not tracked but movie shown here as comparison to Movie S15 and S16. Dye labelled naïve lymphocytes transferred 24 hours prior to imaging shown in gray and Confetti reporter colors are as in Movie S7. 50 μm scale bar and time in min:sec indicated on bottom.

**Movie S14.**

Multiphoton imaging (60 μm stack) of another *Arhgef1*^+/–^ Cre-ERT2^Aicda^ *Rosa26*^Confetti/Confetti^ mouse, displayed as in Movie S13. Stacks were acquired for 63 min at 35 second intervals. 50 μm scale bar and time in min:sec indicated on bottom.

**Movie S15.**

Multiphoton imaging (60 μm stack) of a *Arhgef1*^–/–^ Cre-ERT2^Aicda^ *Rosa26*^Confetti/Confetti^ popliteal LN. Stacks were acquired for 117 min at 35 second intervals. Side by side display shows stacks without (left) and with (right) transferred naïve lymphocytes in gray. Movie shown without transferred naïve lymphocytes for visual clarity of Confetti reporter+ cells outside GCs. Cells were not tracked but cells outside GCs were used to compare naïve and GC B cell size and morphology. 50 μm scale bar and time in min:sec indicated on bottom.

**Movie S16.**

Multiphoton imaging (60 μm stack) of another *Arhgef1*^–/–^ Cre-ERT2^Aicda^ *Rosa26*^Confetti/Confetti^ mouse, displayed as in Movie S15. Stacks were acquired for 117 min at 35 second intervals. 50 μm scale bar and time in min:sec indicated on bottom.

**Movie S17.**

Multiphoton imaging (55 μm stack) of a Cre-ERT2^S1pr2^ *Rosa26*^Ai14/+^ popliteal LN (day 10 post NP-KLH footpad immunization, day 9 gavage with 10 mg tamoxifen), related to Extended Data Figure 2H. Stacks were acquired for 37 min at 32 second intervals. Ai14+ cells are in orange and second harmonic in green. CellTracker Deep Red and CellTrace Violet labelled purified transferred naïve B cells (18 hours post transfer) are in magenta and cyan, respectively. 50 μm scale bar and time in min:sec indicated on bottom.
